# *Cis*-eQTL-based trans-ethnic meta-analysis reveals novel genes associated with breast cancer risk

**DOI:** 10.1371/journal.pgen.1006690

**Published:** 2017-03-31

**Authors:** Joshua D. Hoffman, Rebecca E. Graff, Nima C. Emami, Caroline G. Tai, Michael N. Passarelli, Donglei Hu, Scott Huntsman, Dexter Hadley, Lancelote Leong, Arunabha Majumdar, Noah Zaitlen, Elad Ziv, John S. Witte

**Affiliations:** 1 Department of Epidemiology and Biostatistics, University of California San Francisco, San Francisco, CA, United States of America; 2 Program in Biological and Medical Informatics, University of California San Francisco, San Francisco, CA, United States of America; 3 Department of Epidemiology, Geisel School of Medicine at Dartmouth, Hanover, NH, United States of America; 4 Department of Medicine, University of California San Francisco, San Francisco, CA, United States of America; 5 Department of Pediatrics, University of California San Francisco, San Francisco, CA, United States of America; 6 Department of Urology, University of California San Francisco, San Francisco, CA, United States of America; 7 Institute for Human Genetics, University of California San Francisco, San Francisco, CA, United States of America; Case Western Reserve University School of Medicine, UNITED STATES

## Abstract

Breast cancer is the most common solid organ malignancy and the most frequent cause of cancer death among women worldwide. Previous research has yielded insights into its genetic etiology, but there remains a gap in the understanding of genetic factors that contribute to risk, and particularly in the biological mechanisms by which genetic variation modulates risk. The National Cancer Institute’s “Up for a Challenge” (U4C) competition provided an opportunity to further elucidate the genetic basis of the disease. Our group leveraged the seven datasets made available by the U4C organizers and data from the publicly available UK Biobank cohort to examine associations between imputed gene expression and breast cancer risk. In particular, we used reference datasets describing the breast tissue and whole blood transcriptomes to impute expression levels in breast cancer cases and controls. In trans-ethnic meta-analyses of U4C and UK Biobank data, we found significant associations between breast cancer risk and the expression of *RCCD1* (joint *p*-value: 3.6x10^-06^) and *DHODH* (*p*-value: 7.1x10^-06^) in breast tissue, as well as a suggestive association for *ANKLE1* (*p*-value: 9.3x10^-05^). Expression of *RCCD1* in whole blood was also suggestively associated with disease risk (*p*-value: 1.2x10^-05^), as were expression of *ACAP1* (*p*-value: 1.9x10^-05^) and *LRRC25* (*p*-value: 5.2x10^-05^). While genome-wide association studies (GWAS) have implicated *RCCD1* and *ANKLE1* in breast cancer risk, they have not identified the remaining three genes. Among the genetic variants that contributed to the predicted expression of the five genes, we found 23 nominally (*p*-value < 0.05) associated with breast cancer risk, among which 15 are not in high linkage disequilibrium with risk variants previously identified by GWAS. In summary, we used a transcriptome-based approach to investigate the genetic underpinnings of breast carcinogenesis. This approach provided an avenue for deciphering the functional relevance of genes and genetic variants involved in breast cancer.

## Author summary

There is a clear genetic basis of breast cancer, and previous work has identified numerous genetic variants that increase risk of this common disease. However, much of the underlying genetic variation in breast cancer remains unexplained. To address this void, as part of the National Cancer Institute’s “Up for a Challenge” (U4C) competition, we undertook a large-scale study of genetically regulated gene expression and breast cancer. Specifically, we estimated gene expression levels based on germline genetics for subjects in the seven breast cancer studies provided by U4C and for subjects in the UK Biobank. We then evaluated associations between gene expression and breast cancer and detected three novel and two known breast cancer genes. These genes exhibit potential biological mechanisms for impacting breast carcinogenesis. Our work highlights the value of leveraging different sources of data to more thoroughly study the genetic basis of complex diseases.

## Introduction

Breast cancer is the most common solid organ malignancy and the most frequent cause of cancer death among women worldwide [1]. Family history is among the strongest known risk factors for breast cancer. Individuals with a first-degree relative affected by the disease have a roughly two-fold increased risk of developing breast cancer themselves, and a more extensive family history or having relatives diagnosed at an earlier age confers yet greater risk [2–4]. A recent twin study estimated the heritability of breast cancer to be 31% [5], but the combination of rare variants (e.g., in *BRCA1*, *BRCA2*) identified from linkage studies (summarized in [6]) and common single nucleotide polymorphisms (SNPs) at roughly 100 loci identified from genome-wide association studies (GWAS; summarized in [7]) explain only one-third of the excess familial risk of disease [8]. Thus, a substantial gap remains in the understanding of the genetic factors that contribute to breast cancer risk.

The National Cancer Institute’s Up for a Challenge (U4C) competition offered a unique opportunity to further elucidate the genetic basis of breast cancer. Seven ethnically diverse GWAS datasets were made available in dbGaP and participants were challenged to use innovative approaches to identify novel loci, genes, and/or genomic features involved in breast cancer susceptibility. Our group leveraged the U4C genotype data along with gene expression datasets to search for evidence of additional genes involved in breast cancer susceptibility.

Recently, methods have been developed to leverage genotypic data toward imputing gene expression that can then be evaluated in association studies [9]. These methods are based on strong evidence that expression quantitative trait loci (eQTLs), which contribute to regulating gene expression levels, account for a substantial portion of the risk of various disease phenotypes [10–12]. A reference dataset with genotype and gene expression data is used to derive a set of SNPs that optimally predicts the expression of each gene. These SNPs can then be used to impute genetically regulated gene expression in datasets without measured expression data, and these imputed values can then be tested for associations with a phenotype of interest. Evaluating gene expression with respect to breast cancer risk has the potential to offer insights distinct from those available from traditional GWAS. First, associations with gene expression have clear functional interpretations. In contrast, the functional relevance of SNPs discovered by GWAS usually remains unclear. Second, association testing for genes substantially reduces the multiple testing burden relative to single variant approaches. Third, association testing for gene expression allows for rational combination of multiple SNPs, which may help to enhance weak signals.

We conducted a transcriptome-wide association study of gene expression and breast cancer risk by applying an innovative method called PrediXcan [9] that uses eQTL reference transcriptome datasets to impute genetically regulated expression. We used reference expression data from breast tissue and whole blood to identify the SNPs that predict gene expression. We then used the U4C datasets combined with data from the UK Biobank to search for genes for which predicted expression is associated with susceptibility to breast cancer. The approach provided an avenue for deciphering the functional relevance of both genes and SNPs involved in breast cancer development.

## Results

### Transcriptome-wide imputation in U4C and UK Biobank data

After splitting the GWAS of Breast Cancer in the African Diaspora (African Diaspora), Breast and Prostate Cancer Cohort Consortium GWAS (BPC3), and Multiethnic Cohort GWAS in African Americans, Latinos, and Japanese (MEC) datasets into sub-populations, and excluding the Nurses’ Health Study (NHS2) sub-population from the BPC3 (because it was already included in the Cancer Genetic Markers of Susceptibility Breast Cancer GWAS [CGEMS] dataset), we imputed gene expression into 14 separate discovery studies with a total of 12,079 breast cancer cases and 11,442 controls. In addition, we used 3,370 cases and 19,717 controls from the publicly available UK Biobank cohort study as a replication population [13]. Additional details of the study populations, genotyping, and quality control (QC) process are provided in **Table 1** and the Materials and Methods section.

**Table 1:** Characteristics of the Up for a Challenge datasets (discovery) and the UK Biobank (replication).

Dataset (Source Dataset)	Race / Ethnicity	# Cases^a^	# Controls^a^	Genotyping Platform
*Discovery*				
AABC (AABC)	African	2,755	2,461	Illumina Human1M-Duo BeadChip
African (African Diaspora)	African	699	606	Illumina HumanOmni2.5-Quad
African American / Barbadian (African Diaspora)	African	934	1,400	Illumina HumanOmni2.5-Quad
CGEMS (CGEMS)	European	1,125	1,126	Illumina HumanHap550
CPSII (BPC3)	European	289	292	HumanHap550; HumanHap 660
EPIC (BPC3)	European	501	491	HumanHap550; HumanHap 660
Latina Admixture (Latina Admixture)	Latina	800	365	Affymetrix GWAS SNP Array 6.0
MEC–European (BPC3)	European	85	98	HumanHap550; HumanHap 660
MEC–Japanese (MEC)	East Asian	885	822	Human660W; Human-1M
MEC–Latina (MEC)	Latina	520	544	Human660W; Human-1M
NHS2 (BPC3)	European	71	372	HumanHap550; HumanHap 660
PBCS (BPC3)	European	532	495	HumanHap550; HumanHap 660
PLCO (BPC3)	European	252	337	HumanHap550; HumanHap 660
Shanghai (Shanghai)	East Asian	2,631	2,033	Affymetrix GWAS SNP Array 6.0
*Replication*				
UK Biobank	European	3,370	19,717	UK BiLEVE Axiom; UK Biobank Axiom

Abbreviations: AABC: African American Breast Cancer GWAS; African Diaspora: GWAS of Breast Cancer in the African Diaspora; BPC3: Breast and Prostate Cancer Cohort Consortium GWAS; CGEMS: Cancer Genetic Markers of Susceptibility Breast Cancer GWAS; CPSII: Cancer Prevention Study II; EPIC: European Prospective Investigation into Cancer and Nutrition; GWAS: genome-wide association study; Latina Admixture: San Francisco Bay Area Latina Breast Cancer Study; MEC: Multiethnic Cohort GWAS in African Americans, Latinos, and Japanese; NHS2: Nurses' Health Study 2; PBCS: Polish Breast Cancer Study; PLCO: Prostate, Lung, Colorectal, and Ovarian Cancer Screening Trial; Shanghai: Shanghai Breast Cancer Genetics Study; SNP: single nucleotide polymorphism

^a^ After all quality control steps

The developers of PrediXcan previously determined the *cis*-eQTL SNPs relevant to the estimation of gene expression in 44 distinct tissue types. The weights that should be applied to each SNP to impute transcript levels in other datasets are maintained in the publicly available database PredictDB. For our study, we elected to use the weights developed based on gene expression in breast tissue and, separately, in whole blood. We used the former for its direct relevance to breast cancer (developed based on n = 173 samples) and the latter because the weights were developed based on the largest number of samples among all tissues (n = 922).

Weights for the prediction of breast tissue expression were available for 4,473 genes based on 102,762 unique SNPs. The mean expected correlation between imputed transcript levels and true gene expression across all transcripts was 0.097. Regarding the prediction of whole blood expression, weights were available for 9,791 genes based on 249,696 unique SNPs. The mean expected correlation between imputed transcript levels and true gene expression across all transcripts was 0.145.

### Transcriptome-wide associations with breast cancer risk

A meta-analysis of the U4C discovery datasets yielded 280 transcripts with imputed breast tissue levels nominally (*p*-value < 0.05) associated with breast cancer risk (**S1A Table**). We evaluated all of these genes for associations in the UK Biobank data. Our genomic inflation factor was 1.07 (**λ**_1000_ = 1.01). All genes with a *p*-value < 0.10 in this replication cohort and effect estimates in the same direction as the discovery effect were included in a combined meta-analysis of discovery and replication. **Table 2** describes the three genes for which the combined meta-analysis showed evidence of an association with breast cancer. Decreased expression levels of *RCCD1* (*p*-value: 3.6x10^-06^) and *DHODH* (*p*-value: 7.1x10^-06^) showed significant associations with breast cancer risk based on a Bonferroni-corrected significance threshold (0.05 / 4,473 = 1.1x10^-05^), and higher expression levels of *ANKLE1* demonstrated a suggestive association (*p*-value: 9.3x10^-05^). The *DHODH* association was largely driven by the discovery dataset (*p*-value: 2.4x10^-05^) with little contribution from replication (*p*-value: 0.056). Estimates from each of the discovery datasets and the replication dataset are presented in **S1 Fig** for each of the three genes. While *RCCD1* and *ANKLE1* have been implicated by GWAS of breast cancer risk, *DHODH* has not been previously identified.

**Table 2:** Effect estimates and standard errors for gene expression suggestively (*p*-value < 1.0x10^-04^) associated with breast cancer risk in a meta-analysis of the Up for a Challenge and UK Biobank datasets.

		# SNPs in	Imputation	U4C		UK Biobank		Meta-analysis	
Gene	Location^a^	Prediction	Quality^b^	Beta (SE)	*p*-value	Beta (SE)	*p*-value	Beta (SE)	*p*-value
*Breast Tissue Gene Expression*									
*RCCD1*	15q26.1	24	0.16	-0.11 (0.038)	5.8x10^-03^	-0.24 (0.057)	2.6x10^-05^	-0.15 (0.032)	3.6x10^-06^
*DHODH*	16q22.2	7	0.026	-0.52 (0.12)	2.4x10^-05^	-0.29 (0.15)	0.056	-0.43 (0.095)	7.1x10^-06^
*ANKLE1*	19p13.11	6	0.081	0.19 (0.093)	0.044	0.43 (0.12)	1.9x10^-04^	0.28 (0.072)	9.3x10^-05^
*Whole Blood Gene Expression*									
*RCCD1*	15q26.1	20	0.35	-0.074 (0.026)	4.7x10^-03^	-0.14 (0.039)	2.7x10^-04^	-0.095 (0.022)	1.2x10^-05^
*ACAP1*	17p13.1	19	0.39	0.098 (0.037)	7.9x10^-03^	0.11 (0.033)	7.9x10^-04^	0.11 (0.025)	1.9x10^-05^
*LRRC25*	19p13.11	33	0.35	0.086 (0.029)	2.7x10^-03^	0.094 (0.034)	6.5x10^-03^	0.089 (0.022)	5.2x10^-05^

Abbreviations: SE: standard error; SNP: single nucleotide polymorphism; U4C: Up for a Challenge

^a^ According to human reference genome GRCh37/hg19

^b^ r^2^ estimate derived from 10 fold cross-validation of true gene expression and predicted gene expression

The imputed expression of genes based on whole blood yielded no statistically significant associations with breast cancer risk after multiple testing correction (Bonferroni significance threshold = 0.05 / 9,791 = 5.1x10^-06^) (**S1B Table**). Our genomic inflation factor was 1.06 (**λ**_1000_ = 1.01). However, **Table 2** shows results for three genes that showed suggestive evidence of an association (*p*-value < 1.0x10^-04^). Notably, decreased expression levels of *RCCD1* in whole blood (as in breast tissue) were suggestively associated with breast cancer risk (*p*-value: 1.2x10^-05^). Furthermore, we found that higher expression levels of *ACAP1* (*p*-value: 1.9x10^-05^) and *LRRC25* (*p*-value: 5.2x10^-05^) were suggestively associated with an increased risk of breast cancer. Estimates from each of the discovery datasets and the replication dataset are presented in **S2 Fig** for each of the three genes. Neither *ACAP1* nor *LRRC25* have previously been implicated by GWAS of breast cancer risk.

The volcano plots in **S3 Fig** depict the U4C and UK Biobank meta-analysis summary statistics for 4,469 breast tissue transcripts and 9,768 whole blood transcripts. Outliers with beta estimates outside three standard deviations from the mean were excluded from the plots–four for breast tissue and 23 for whole blood. The x-axis gives the beta effect sizes reflecting the fold change in gene expression between cases and controls, and the y-axis plots the corresponding -log_10_(*p*-value). **S3 Fig** is thus illustrative of the differential expression between cases and controls for genes across the transcriptome. For breast tissue expression (**S3A Fig**), we saw few genes beyond those noted above showing any evidence of association. In contrast, the distribution of *p*-values for whole blood expression (**S3B Fig**) was slightly broader, albeit with a more stringent threshold for statistical significance. However, among those genes significantly or suggestively associated with breast cancer risk, the magnitudes of the effect sizes were larger for breast tissue expression (|Beta| ≥ 0.15) than for whole blood expression (|Beta| ≤ 0.11; **Table 2**). For the 2,840 genes that overlapped, the correlation of the betas for the breast tissue and whole blood analyses was significant (r^2^ = 0.32; *p*-value: 2.2x10^-16^).

We tested for heterogeneity of the associations across studies in the meta-analysis of the U4C datasets alone, and in the meta-analysis combined with the UK data. These analyses did not indicate any statistically significant heterogeneity (*p*-values > 0.10). Furthermore, we did not detect heterogeneity within ancestry groups (*p*-values > 0.15), except for *ANKLE1* in the European only meta-analysis (*p*-value: 0.022). Upon restricting the analysis to women with ER negative breast cancer, however, we no longer found significant heterogeneity (*p*-value: 0.32).

### Single variants that predict expression and breast cancer risk

**Table 2** indicates the number of SNPs identified by PredictDB for optimal prediction of the genetically regulated expression of each of the genes showing suggestive associations with breast cancer risk. PrediXcan uses an elastic net method to determine the best set of SNPs for predicting gene expression. Because elastic net allows for highly correlated variables in prediction models, some of the SNPs are in high linkage disequilibrium (LD). We evaluated associations between each of the SNPs and breast cancer risk (**S2 Table**); those achieving nominal (*p*-value < 0.05) significance in a meta-analysis of the U4C and UK Biobank data are displayed in **Table 3**. The tables also indicate the proportion of total weight attributed to each SNP in the gene prediction models. The sum of the relative weights for all SNPs contributing to the prediction of any given gene always equals to one, and the SNP ranking remains static. Raw weights used for gene expression prediction can be found within the GTEx and DGN PredictDB databases.

**Table 3:** SNPs nominally (*p*-value < 0.05) associated with breast cancer risk that contribute to expression of genes suggestively associated with breast cancer risk.

		Proportion		U4C		UK Biobank			Meta-analysis	
SNP	Alleles^a^	of Weight^b^	EAF^c^	OR (95% CI)	*p*-value	EAF^c^	OR (95% CI)	*p*-value	OR (95% CI)	*p*-value
RCCD1 *at 15q26.1 (Breast Tissue)*										
rs3826033^d^	G / A	0.13	0.32	0.92 (0.88, 0.98)	4.1x10^-03^	0.13	0.86 (0.79,0.93)	2.3x10^-04^	0.90 (0.86,0.94)	9.5x10^-06^
rs2290202^d^	G / T	0.24	0.3	0.93 (0.89, 0.98)	5.3x10^-03^	0.13	0.86 (0.79,0.93)	1.9x10^-04^	0.91 (0.88,0.95)	1.7x10^-05^
rs4347602	A / C	0.025	0.72	0.94 (0.90,0.98)	6.5x10^-03^	0.77	0.96 (0.90,1.02)	0.16	0.94 (0.91,0.98)	2.4x10^-03^
rs11207^d^	C / T	0.030	0.35	0.97 (0.93, 1.02)	0.21	0.24	0.93 (0.87,0.98)	0.015	0.96 (0.93,0.99)	0.016
DHODH *at 16q22.2 (Breast Tissue)*										
rs3213422	C / A	0.56	0.42	0.92 (0.88,0.96)	2.8x10^-05^	0.48	0.95 (0.90,1.00)	0.039	0.93 (0.90,0.96)	4.5x10^-06^
rs2240243	G / A	0.055	0.47	0.93 (0.89,0.97)	2.7x10^-04^	0.34	0.98 (0.93,1.04)	0.53	0.95 (0.92,0.98)	1.0x10^-03^
rs12708928	C / A	0.019	0.47	0.93 (0.89,0.96)	2.5x10^-04^	0.34	0.99 (0.93,1.04)	0.59	0.95 (0.92,0.98)	1.2x10^-03^
ANKLE1 *at* 19p13.11 *(Breast Tissue)*										
rs34084277^d^	A / G	0.23	0.19	1.09 (1.02,1.15)	7.1x10^-03^	0.19	1.11 (1.04,1.18)	2.0x10^-03^	1.10 (1.05,1.14)	4.7x10^-05^
rs8170^d^	G / A	0.26	0.19	1.08 (1.02,1.15)	7.2x10^-03^	0.19	1.11 (1.04,1.18)	2.6x10^-03^	1.09 (1.05,1.14)	6.3x10^-05^
RCCD1 *at 15q26.1 (Whole Blood)*										
rs3826033^d^	G / A	0.33	0.32	0.92 (0.88,0.98)	4.1x10^-03^	0.13	0.86 (0.79,0.93)	2.3x10^-04^	0.90 (0.86,0.94)	9.5x10^-06^
rs2290202^d^	G / T	0.29	0.3	0.93 (0.89,0.98)	5.3x10^-03^	0.13	0.86 (0.79,0.93)	1.9x10^-04^	0.91 (0.88,0.95)	1.7x10^-05^
rs7180016^d^	G / A	0.012	0.49	0.97 (0.93,1.01)	0.13	0.16	0.90 (0.84,0.97)	5.7x10^-03^	0.95 (0.92,0.99)	7.3x10^-03^
rs11073961	A / G	0.049	0.35	0.97 (0.93,1.01)	0.21	0.27	0.92 (0.87,0.98)	7.5x10^-03^	0.95 (0.93,0.99)	9.9x10^-03^
rs11207^d^	C / T	0.0092	0.35	0.97 (0.93,1.02)	0.21	0.24	0.93 (0.87,0.98)	0.015	0.96 (0.93,0.99)	0.016
rs2285937^d^	A / G	0.0064	0.46	0.98 (0.94,1.02)	0.31	0.16	0.90 (0.84,0.97)	4.9x10^-03^	0.96 (0.93,0.99)	0.023
rs3809583	A / G	0.0035	0.36	0.97 (0.93,1.01)	0.12	0.32	0.96 (0.91,1.01)	0.15	0.96 (0.93,1.00)	0.035
ACAP1 *at 17p13.1 (Whole Blood)*										
rs35776863	A / G	0.49	0.85	1.08 (1.00,1.16)	0.045	0.77	1.11 (1.04,1.18)	0.15	1.10 (1.04,1.15)	1.4x10^-04^
rs9892383	C / T	0.030	0.76	1.04 (0.98,1.09)	0.17	0.73	1.10 (1.03,1.18)	0.76	1.06 (1.02,1.11)	3.6x10^-03^
rs5412	G / A	0.060	0.12	1.04 (0.97,1.12)	0.26	0.17	1.09 (1.02,1.17)	0.12	1.07 (1.02,1.12)	8.0x10^-03^
rs4791423	A / C	0.0068	0.45	1.04 (1.00,1.09)	0.033	0.34	1.03 (0.98,1.09)	0.55	1.04 (1.01,1.08)	0.018
rs35721044	T / C	0.031	0.84	1.11 (1.02,1.22)	0.012	0.76	1.03 (0.97,1.10)	0.16	1.06 (1.01,1.12)	0.019
LRRC25 *at 19p13.11 (Whole Blood)*										
rs11668719	C / T	0.25	0.5	1.06 (1.01,1.11)	0.011	0.54	1.10 (1.05,1.16)	1.87x10^-04^	1.08 (1.04,1.12)	1.2x10^-05^
rs7257932^d^	A / G	0.091	0.55	1.05 (1.01,1.10)	0.011	0.67	1.08 (1.02,1.14)	7.01x10^-03^	1.06 (1.03,1.10)	2.5x10^-04^
rs13344313	A / G	0.16	0.68	1.06 (1.02,1.11)	6.6x10^-03^	0.71	1.04 (0.98,1.10)	0.20	1.05 (1.02,1.09)	3.2x10^-03^
rs3795026	C / T	<0.001	0.54	1.04 (1.00,1.08)	0.051	0.68	1.05 (0.99,1.11)	0.12	1.04 (1.01,1.08)	0.013
rs7251067	A / G	0.031	0.85	1.00 (0.95,1.06)	0.94	0.86	1.14 (1.06,1.23)	6.70x10^-04^	1.05 (1.00,1.10)	0.041

Abbreviations: CI: confidence interval; EAF: effect allele frequency; OR: odds ratio; SNP: single nucleotide polymorphism; U4C: Up for a Challenge

^a^ Reference allele / effect allele

^b^ Proportion of total weight attributed to SNP in gene prediction model

^c^ Effect allele frequency in controls

^d^ Previously implicated in breast cancer or in high linkage disequilibrium (r^2^ > 0.5 in 1000 Genomes Phase 3 populations) with known risk variants

**Fig 1:**
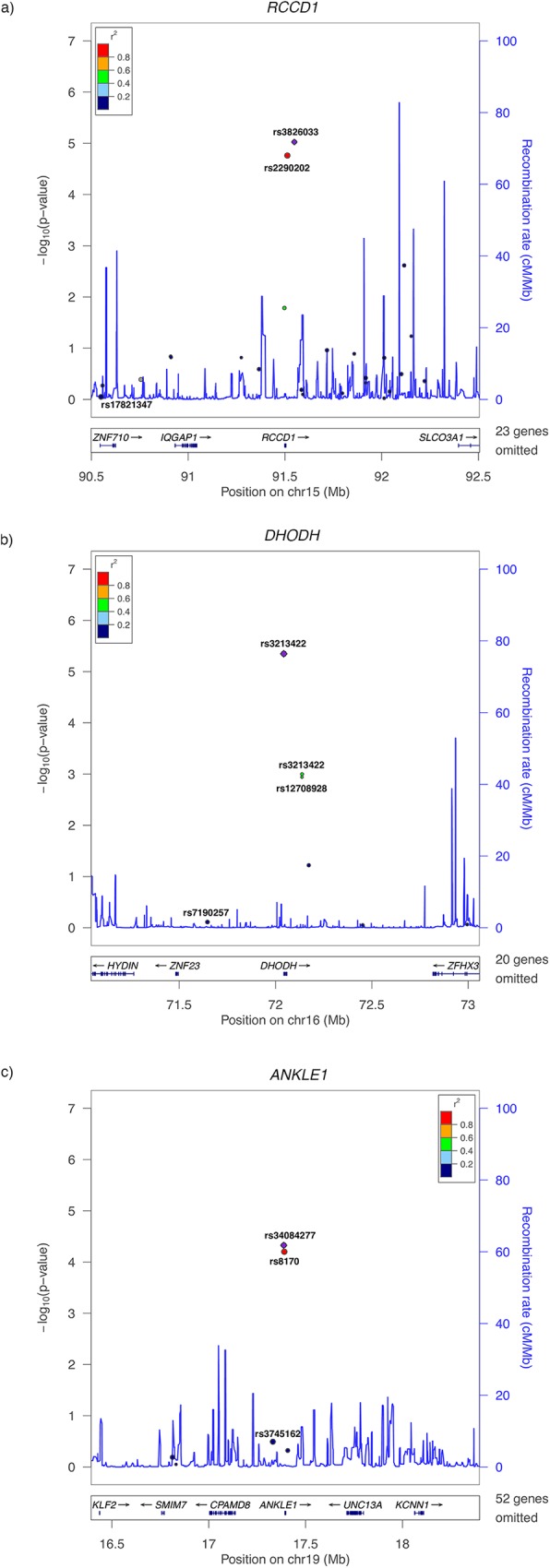
LocusZoom plots of SNPs contributing to the breast tissue expression of (A) *RCCD1* at 15q26.1, (B) *DHODH* at 16q22.2, and (C) *ANKLE1* at 19p13.11. The x-axis displays the location of the modeled eQTL SNPs relative to the genes of interest discovered in analyses breast tissue expression. The y-axis indicates the strength of association between the SNPs and breast cancer risk. Each point is sized based on the relative contribution of the variant to gene expression.

**Fig 1** displays the location of eQTL SNPs for the genes for which breast tissue expression levels were associated with breast cancer risk. The y-axis indicates the strength of association between the SNPs and breast cancer risk and each point is sized based on the relative contribution of the variant to gene expression. Among the 24 SNPs predicting expression of *RCCD1*, rs3826033 showed the strongest association with breast cancer risk (joint *p*-value: 9.5x10^-06^). It contributed 13% of the weight for predicting *RCCD1* expression, third only to rs2290202 (24%) and rs17821347 (16%). rs2290202 was also strongly associated with breast cancer risk (*p*-value: 1.7x10^-05^). It should be noted that rs3826033 and rs2290202 are in high LD (r^2^ = 0.97 in 1000 Genomes Phase 3 European populations), and both SNPs are within close proximity of *RCCD1* relative to the other eQTL SNPs. In contrast, rs17821347 is furthest away from *RCCD1* among SNPs predicting *RCCD1* expression and showed no evidence of an association with breast cancer risk (*p*-value: 0.89). Among the remaining *RCCD1* eQTLs, only rs4347602 showed a nominal association (*p*-value: 2.4x10^-03^); it has not previously been identified by GWAS.

All three nominal associations that we identified for SNPs predicting *DHODH* expression in breast tissue have not been implicated by GWAS. rs3213422 showed the strongest signal (*p*-value: 4.5x10^-06^) and also contributed the majority of the weight (56%) among the seven SNPs predicting of *DHODH* expression. Both rs2240243 and rs12708928 (r^2^ = 1.0) are in moderate LD with rs3213422 (r^2^ = 0.50 for both variants) and also showed evidence of associations with breast cancer risk (*p*-values: 1.0x10^-03^ and 1.3x10^-03^ respectively). After rs3213422, the second most weight was contributed by rs7190257 (16%), which showed no evidence of association (*p*-value: 0.77).

We identified two SNPs out of six total eQTL SNPs predicting *ANKLE1* expression in breast tissue that were associated with breast cancer; both have been previously associated with breast cancer risk [14–19]. The SNPs, rs34084277 (*p*-value: 4.7x10^-05^) and rs8170 (*p*-value: 6.3x10^-05^), are in perfect LD (r^2^ = 1.0) and both contributed substantial weight to the prediction of *ANKLE1* expression (23% and 26% respectively). Notably, rs3745162 also contributed substantial weight (24%), but showed no evidence of an association with breast cancer risk (*p*-value: 0.32).

**Fig 2:**
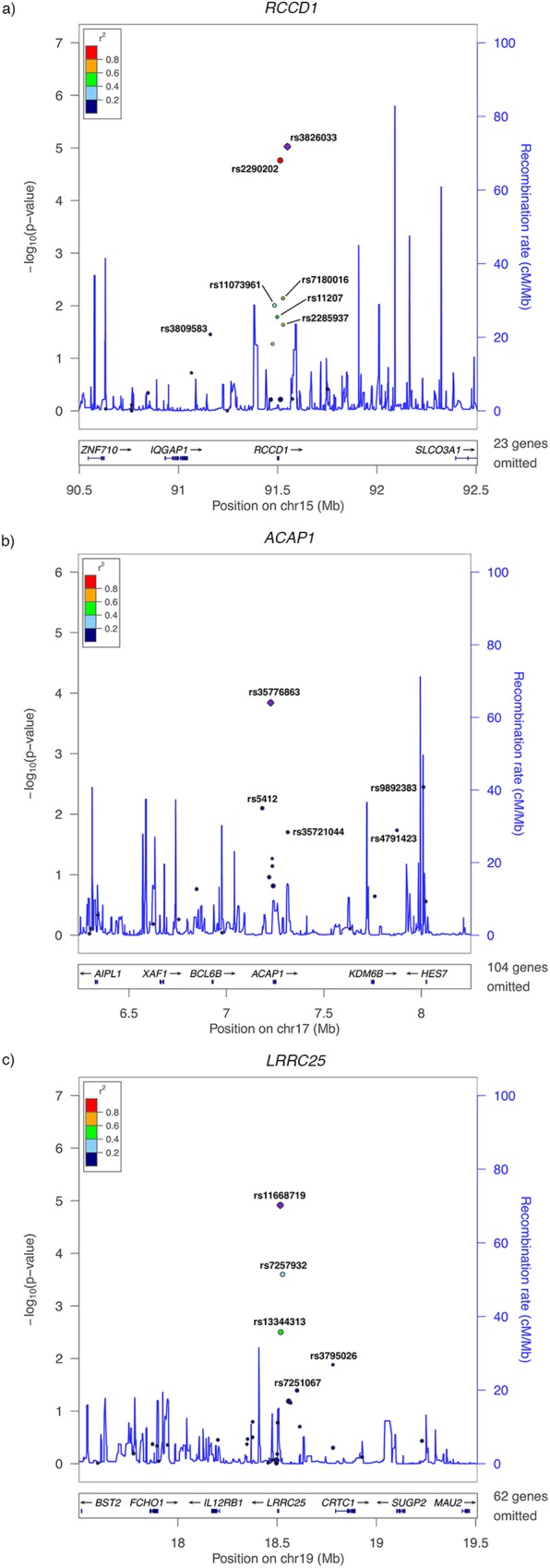
LocusZoom plots of SNPs contributing to the whole blood expression of (A) *RCCD1* at 15q26.1, (B) *ACAP1* at 17p13.1, and (C) *LRRC25* at 19p13.11. The x-axis displays the location of the modeled eQTL SNPs relative to the genes of interest discovered in analyses of whole blood expression. The y-axis indicates the strength of association between the SNPs and breast cancer risk. Each point is sized based on the relative contribution of the variant to gene expression.

**Fig 2** depicts the genes for which whole blood expression levels were associated with breast cancer risk. Among the 20 *RCCD1* eQTL SNPs, rs3826033 (p-value: 4.1x10^-03^) and rs2290202 (p-value: 5.3x10^-03^) contributed the most weight to prediction (33% and 29% respectively) and were the most strongly associated with breast cancer risk. The other SNPs showing evidence of an association were rs7180016 (*p*-value: 7.3x10^-03^), rs11073961 (*p*-value: 9.9x10^-03^), rs11207 (*p*-value: 0.016), rs2285937 (*p*-value: 0.023), and rs3809583 (*p*-value: 0.035). rs3826033, rs2290202, and rs11207 were included in the both the breast tissue and the whole blood prediction models for *RCCD1* expression. Only rs11073961 and rs3809583 have not been previously implicated in breast cancer GWAS.

Among the 19 *ACAP1* whole blood eQTL SNPs, five were nominally associated with breast cancer risk. Most noteworthy was rs35776863, which not only had the strongest association with breast cancer risk (*p*-value: 1.4x10^-04^), but also contributed nearly half of the weight for predicting *ACAP1* expression (49%). The other SNPs showing evidence of an association were rs9892383 (*p*-value: 3.6x10^-03^), rs5412 (*p*-value: 8.0x10^-03^), rs4791423 (*p*-value: 0.018), and rs35721044 (*p*-value: 0.019). None of these SNPs have been previously implicated in breast cancer GWAS.

Out of 33 *LRRC25* whole blood eQTL SNPs, five showed evidence of an association with breast cancer risk. Again, the SNP that contributed the most weight (25%), rs11668719, also showed the strongest association signal with disease risk (*p*-value: 1.2x10^-05^). The next two strongest signals were for SNPs in moderate LD with rs11668719, namely rs7257932 (r^2^ = 0.39; *p*-value: 2.5x10^-04^), which is the only SNP predicting *LRRC25* expression previously implicated in breast cancer GWAS, and rs13344313 (r^2^ = 0.43; *p*-value: 3.2x10^-03^). Also suggestively associated with breast cancer risk, albeit contributing less than 0.1% of the weight for predicting *LRRC25* expression, was rs3795026 (*p*-value: 0.013). The last SNP nominally associated with breast cancer risk was rs7251067 (*p*-value: 0.041).

## Discussion

In this transcriptome-wide association study, we identified five genes for which genetically regulated expression levels may be associated with breast cancer risk. We also found 23 unique SNPs contributing to the expression levels of these five genes that were associated with disease. Out of the 23 SNPs, seven in breast cancer genes identified by GWAS and one in a breast cancer gene previously unidentified by GWAS have been previously implicated in breast cancer or are in high LD (r^2^ > 0.50 in 1000 Genomes Phase 3 populations) with known risk variants. The remaining SNPs have not been previously associated with breast cancer risk.

We found that lower predicted expression of *RCCD1* (i.e., RCC1 domain containing 1) in both breast tissue and whole blood was associated with increased breast cancer risk. This finding supports limited existing evidence for the role of *RCCD1* in breast cancer. A 2014 GWAS of East Asian women reported a genome-wide significant association for rs2290203, which is 5,712 bp downstream of *RCCD1* on 15q26.1 [20]. The authors then replicated the association in a European population. They also showed a correlation between rs2290203 and expression of *RCCD1* [20], which supported a previous eQTL analysis of human monocytes that indicated that rs2290203 is a *cis*-eQTL for *RCCD1* [21]. A more recent study identified an association between rs8037137, another 15q26.1 SNP in moderate LD with rs2290203 (r^2^ = 0.59 in 1000 Genomes Phase 3 European populations), and both breast and ovarian cancer [7]. The effect alleles of both rs2290203 and rs8037137 decrease *RCCD1* expression [7,20], aligning with our finding that lower *RCCD1* expression is associated with increased breast cancer risk. Neither rs2290203 nor rs8037137 was among the SNPs included in PredictDB for the prediction of *RCCD1* expression. However, these SNPs are in LD with *RCCD1* eQTL SNPs that were included in the prediction models, namely rs2290202 (r^2^ = 0.59 for rs2290203, r^2^ = 0.99 for rs8037137) and rs3826033 (r^2^ = 0.57, r^2^ = 0.96). The PrediXcan breast tissue model explains approximately 30% of the variance in *RCCD1* expression, and rs2290202 and rs3826033 account for approximately 37% of that variation. The histone demethylase complex formed by RCCD1 protein with KDM8 is important for chromosomal stability and fidelity during mitosis division [22]. It is thus plausible that lower expression of *RCCD1* could lead to errors in cell division that could potentially increase the risk of breast cancer. Future studies should evaluate the specific mechanisms whereby reduced *RCCD1* expression could be associated with breast cancer risk.

*ANKLE1* (i.e., ankyrin repeat and LEM domain containing 1) has been previously implicated in breast cancer. Both *cis*-eQTLs for *ANKLE1*, rs8170 and rs34084277, among several other SNPs in the 19p13.11 region, have been identified as breast cancer risk variants in several GWAS[8,14–19,23–25]. Little experimental evidence exists regarding associations between over- or under-expression of *ANKLE1* and cancer risk. In our study, we found that higher expression levels of *ANKLE1* were associated with an increased risk of breast cancer. Variants in the two SNPs positively associated with *ANKLE1* expression in our study were also positively associated with breast cancer risk in previous work by Antoniou *et al*. [14]. With regard to the genotypic association with breast cancer risk, the effect estimates corresponding to the same risk allele were similar. Specifically, for rs8170, the A allele was positively associated with breast cancer in the previous study (OR = 1.28 among *BRCA1* carriers) and our study (OR = 1.08). Although the direction of effect was not previously reported for rs34084277, this variant is in almost perfect LD with rs8170 and shares the same direction of effect in our study (OR = 1.09). *ANKLE1* is an endonuclease involved in DNA damage repair pathways [26]. Its overexpression could therefore perturb the delicate balance required for DNA damage repair. That SNPs in the 19p13.11 locus have also been implicated in ovarian cancer [27,28] implies that *ANKLE1* may also be involved in hormonally-mediated carcinogenic pathways.

To the best of our knowledge, *DHODH*, *ACAP1*, and *LRRC25* have not been implicated in GWAS of breast cancer risk. Even though the imputation quality of *DHODH* (i.e., dihydroorotate dehydrogenase [quinone]), was lowest among the genes of interest in our study, we still identified a statistically significant association between decreased expression levels of *DHODH* in breast tissue and breast cancer risk. The existing literature regarding the directionality of association for *DHODH* and breast cancer is potentially inconsistent; deletion of the 16q22.2 locus has been associated with both better prognosis [29] and increased risk of metastasis [30]. Still, *DHODH* inhibition has been leveraged in the treatment of breast cancer. In particular, a *DHODH* inhibitor called brequinar has been shown to have modest activity in patients with advanced breast cancer [31]. It is thus difficult to reconcile our findings regarding disease risk with those of existing studies of disease progression.

*ACAP1* (i.e., ArfGAP with coiled-coil, ankyrin repeat and PH domains 1) has not been implicated in breast cancer risk, but it has been shown to potentially play a role in disease progression. Its protein product activates the Arf6 protein [32], the expression of which has been shown to be higher in highly invasive breast cancer than in weakly invasive or noninvasive breast cancer and normal mammary epithelial cells [33]. *ACAP1* also interacts with the third cytoplasmic loop of *SLC2A4*/*GLUT4*. *SLC2A4* encodes a protein that functions as an insulin-regulated facilitative glucose transporter; inhibition of this gene affects cell proliferation and cell viability, suggesting a potential biological hypothesis for how *ACAP1* may be involved with breast cancer [34].

*LRRC25* (i.e., leucine rich repeat containing 25) is more than one megabase away from *ANKLE1* at 19p13.11. It is located in a leukocyte-receptor cluster and may be involved in the activation of hematopoietic cells, which play a critical role in innate and acquired immunity [35]. If *LRRC25* overexpression results in an elevated inflammatory response, then it could also increase the risk of breast cancer. In a study of the *cis*-eQTL activity of known cancer loci, the 19p13.11 breast cancer risk SNP rs4808801 was most significantly associated with the expression of *LRRC25* (p-value: 3.2 x 10^-03^) [36]. rs4808801 is in high LD (r^2^ = 0.88 in 1000 Genomes Phase 3 European populations) with the eQTL rs7257932 that we used to impute *LRRC25*.

It is our understanding that ours is the first study to use PrediXcan to impute eQTLs transcriptome-wide toward evaluating associations with cancer. It is important, however, that it be interpreted in the context of some limitations. The weights housed in PredictDB were largely developed based on Caucasian samples. However, no SNPs that were monomorphic in any of the 14 U4C ancestral populations were included in our analysis. Still, whether or not the weights are valid for application in non-Caucasian populations is unclear and requires further study. Furthermore, true gene expression was unmeasured. Rather, our study evaluated estimated genetically regulated gene expression, sometimes with low imputation quality. The mean expected correlation of imputed genetically regulated gene expression and true gene expression is 0.097 for breast tissue and 0.145 for whole blood. For most genes, we would not expect the correlation to approach one given that gene expression is regulated by factors other than germline genetics, but because PrediXcan was only recently developed, an appropriate threshold for usable imputation quality is not yet definitive. In the release of PredictDB used here (dated 8/18/16), the authors only included genes that had a false discovery rate ≤ 5% based on the elastic net models used to generate the SNP weights. With respect to our results, imputation quality seemed related to the number of SNPs included in the gene expression prediction model. It is interesting, however, that we were still able to detect signal for the genes in our study for which expression was predicted by the smallest number of SNPs (*ANKLE1* and *DHODH*). The imputation quality and included genes will likely change as updated versions of PrediXcan and PredictDB become available. How sensitive findings are to PrediXcan updates is an important consideration given that prediction is dependent on the reference panel.

In summary, by employing a transcriptome-wide approach, we identified novel associations for gene expression with breast cancer risk that have not surfaced from traditional GWAS designs. The approach also allowed for the development of new hypotheses regarding biological mechanisms at play in breast carcinogenesis. Future research focusing on the downstream effects of imputed gene expression, such as gene-gene interactions and gene co-expression networks, may further advance the characterization of breast cancer etiology.

## Materials and methods

### Study populations and genotyping

Discovery analyses used all seven dbGaP datasets provided for the purposes of U4C: African American Breast Cancer GWAS (AABC); African Diaspora; CGEMS [37,38]; BPC3 [19,39]; **San Francisco Bay Area Latina Breast Cancer Study** (Latina Admixture); MEC; and Shanghai Breast Cancer Genetics Study (Shanghai). All of the U4C datasets provided case-control status, age, and principal components of race/ethnicity. Genotyping platforms varied by study as outlined in **Table 1**. Imputed genotypic data were also made available for U4C, but we elected to impute each dataset to the same reference panel as described later on.

We used the publicly available UK Biobank as a replication population. The UK Biobank is a cohort of 500,000 persons aged 40 to 69 recruited from across the United Kingdom between 2006 and 2010. Its protocol has been previously described [13]. In brief, every participant was evaluated at baseline in-person visits during which assessment center staff introduced a touch-screen questionnaire, conducted a brief interview, gathered physical measurements, and collected both blood and urine samples.

In an interim data release, UK Biobank has made typed genotypic data available for 152,736 individuals whose blood samples passed QC. Affymetrix genotyped 102,754 of these individuals' samples with the UK Biobank Axiom array [40] and 49,982 with the UK BiLEVE array [41]. The former array is an updated version of the latter; it includes additional novel markers that replace a small fraction of the markers used for genome-wide coverage. In all, the two arrays share over 95% of their marker content, and 806,466 SNPs that passed QC in at least one batch [41].

In addition to the typed data, UK Biobank has released imputed data for 152,249 samples that were not identified as outliers. Imputation was conducted based on a consolidation of the UK10K haplotype and the 1000 Genomes Phase 3 reference panels [42]. It resulted in a dataset of 73,355,667 SNPs, short indels, and large structural variants.

From among the individuals in the UK Biobank with imputed data available, we identified 3,370 European ancestry women diagnosed with breast cancer according to ICD-9 (174) and ICD-10 (C50) codes. Because non-breast cancers are unlikely to metastasize to breast tissue [43], we assumed that all first diagnoses of cancers in the breast were primary malignancies and included women with prior non-breast cancer diagnoses. Of the 3,370 breast cancers included in the analysis, 171 (5.1%) had a previous diagnosis of a separate cancer-related condition. A majority of these were nonmelanoma skin cancers (n = 43) or in situ conditions (n = 50); the number of cases with other malignancies was very low (n = 78, 2.3% of total cases), and including them was thus unlikely to materially alter our findings. We defined European ancestry individuals as those classified as British, Irish, or any other European background according to the baseline questionnaire.

We randomly selected 19,717 controls frequency-matched to cases by five-year age groups from among European ancestry females in the UK Biobank cohort without an ICD9 or ICD10 code for any primary or secondary diagnosis of cancer and with imputed genotypic data.

We excluded from controls any women with a previous cancer to limit the potential for bias arising from a shared genetic basis underlying different cancers. Age at the time of initial assessment was calculated by subtracting year of birth from year of assessment; month and day of birth were unavailable.

### Ethics statement

The Institutional Review Boards of each project that made the data used here publicly available approved the research. Since these are non-identifiable data, we are exempt from Institutional Review Board approval at our home institution.

### Removing duplicates and closely related individuals

For each of the seven U4C datasets and the UK Biobank case-control sub-study, we used the KING toolset to calculate pairwise kinship coefficients and remove subjects with up to second degree familial relationships. We found that all participants of the NHS1 were included in both the CGEMS and BPC3 U4C datasets. We thus excluded the NHS1 from the latter dataset. For related individuals, we retained one individual from the relationship pair for potential inclusion in our analyses.

### Quality control and imputation

As a first QC step for the U4C datasets, we merged all dbGaP consent groups within each of the seven studies and then checked self-reported sex against genotypic data (i.e., the X chromosome). We excluded all individuals with sex discrepancies as well as any individuals with overall call rates < 0.95. Next, we evaluated the rate of heterozygosity for all subjects. Of the seven U4C datasets, some included data from multiple sub-populations or cohorts (i.e., BPC3, MEC, and African Diaspora). As a result, we split BPC3, having already excluded the NHS1, into six datasets (Cancer Prevention Study II [CPSII], European Prospective Investigation into Cancer and Nutrition [EPIC], MEC—European, Nurses' Health Study 2 (NHS2), Polish Breast Cancer Study [PBCS], and Prostate, Lung, Colorectal, and Ovarian Cancer Screening Trial [PLCO]), MEC into two datasets (MEC—Japanese and MEC—Latina), and African Diaspora into two datasets (African and African American / Barbadian). Within the four datasets that we did not split, and in each of the ten newly created split datasets (14 datasets total), we excluded individuals with a heterozygosity rate greater than three standard deviations from the mean rate. Regarding SNP QC, we excluded those with an array genotyping rate < 0.98 in each study, as well as those with a minor allele frequency < 0.02.

Our next step was to ensure that all 14 datasets mapped to the same human reference genome (hg19). We used liftOver to lift datasets mapped to hg18 over to hg19 as necessary. We then ran SHAPEIT for haplotype phasing of each dataset. Finally, we imputed all datasets to the Haplotype Reference Consortium using Minimac3 [44].

Before being made available, UK Biobank data had already undergone extensive individual- and SNP-level QC procedures as previously described [13]. We thus used the data as provided except as outlined in the section below. We also used the imputed data provided by UK Biobank as described in the Study Populations and Genotyping section above.

### Principal component analyses

We implemented principal component analysis to assess genetic ancestry in each of the 14 U4C datasets and in the UK Biobank case-control sub-study of unrelated individuals. To do so, we first LD pruned typed SNPs with r^2^ > 0.2 in PLINK. Then we excluded SNPs with > 0.2% missingness in the U4C datasets and > 1% missingness in the UK Biobank dataset. With the remaining data, we determined the principal components (PC) using EIGENSTRAT within smartpca [45].

Based on the PCs for the U4C datasets, we excluded any individuals outside six standard deviations along any one of the top ten principal components (**S3 Table**). For the UK Biobank dataset, we first focused on the top two PCs to identify any clusters of individuals that may have comprised separate sub-populations. Upon identifying one such cluster, we excluded outliers with a PC eigenvector value greater than seven standard deviations from the mean; doing so excluded individuals in the identified cluster (**S3 Table**).

### Statistical analyses

Details of the PrediXcan method have been previously described [9]. In brief, PrediXcan uses reference datasets in which both genomic variation and gene expression levels have been measured to train additive models of gene expression. The models are constrained using an elastic net method that allows for the inclusion of highly correlated variables. Estimates from the best fit models are stored in the publicly available database PredictDB. The application of PrediXcan to GWAS datasets entails imputing gene expression across the transcriptome using the weights stored in PredictDB and correlating transcript levels with the phenotype of interest.

For these analyses, we accessed the sets of imputation weights referencing the breast tissue transcriptome from the GTEx Project and the set of weights referencing the whole blood transcriptome from the Depression Genes Network(DGN) [46,47]. The versions of PrediXcan and PredictDB used here were dated 6/29/16 and 8/18/16, respectively. We used each set of weights to impute the transcriptome in each of our 14 discovery datasets and in our replication dataset based on the subset of SNPs with imputation quality ≥ 0.3. In each dataset, we performed logistic regression to estimate the associations between imputed transcript levels and breast cancer risk, adjusted for the top ten PCs and age. Finally, we combined the results from the 14 discovery datasets and then included the replication dataset using inverse-variance-weighted fixed-effects meta-analyses. We assessed heterogeneity in the meta-analyses of the discovery U4C datasets, and in the joint meta-analyses with the UK data using Cochran’s Q-test as implemented by METAL [48].

When a joint meta-analysis indicated a suggestive association between expression of a particular gene and breast cancer risk, we evaluated associations between its *cis*-eQTLs and breast cancer risk. Again, we performed logistic regression adjusted for the top ten PCs and age in each dataset and then combined estimates via meta-analysis.

## Supporting information

S1 Table Effect estimates and standard errors for associations nominally (*p*-value < 0.05) significant in a meta-analysis of the discovery Up for a Challenge datasets between breast cancer risk and the imputed expression of genes based on (A) breast tissue and (B) whole blood.
(PDF)

S2 Table Association of breast cancer risk with SNPs that contribute to the expression of (A) *RCCD1* in breast tissue, (B) *DHODH* in breast tissue, (C) *ANKLE1* in breast tissue, (D) *RCCD1* in whole blood, (E) *ACAP1* in whole blood, and (F) *LRRC25* in whole blood.
(PDF)

S3 Table Number of subjects removed from each cohort because of outlier principal components.
(PDF)

S1 Fig Forest plots of PrediXcan results for breast tissue expression of (A) *RCCD1*, (B) *DHODH*, and (C) *ANKLE1*.
(PDF)

S2 Fig Forest plots of PrediXcan results for whole blood expression of (A) *RCCD1*, (B) *ACAP1*, and (C) *LRRC25*.
(PDF)

S3 Fig Volcano plots of PrediXcan results for associations between breast cancer risk and the imputed expression of (A) 4,469 genes based on breast tissue and (B) 9,768 genes based on whole blood (genes with beta estimates outside three standard deviations from the mean were removed from the plots– 4 for breast tissue and 23 for whole blood).
(PDF)
