# Supplementary material for: Cis-eQTL-based trans-ethnic meta-analysis reveals novel genes associated with breast cancer risk
Source: PLoS Genet. 2017 Mar 31;13(3):e1006690. doi: 10.1371/journal.pgen.1006690 (PMC5391966; doi:10.1371/journal.pgen.1006690)
Supplement: S1 Table — Effect estimates and standard errors for associations nominally (p-value < 0.05) significant in a meta-analysis of the discovery Up for a Challenge datasets between breast cancer risk and the imputed expression of genes based on (A) breast tissue and (B) whole blood. (PDF) [file pgen.1006690.s001.pdf]

(A) Breast Tissue Expression

Gene	Location[a]	# SNPs in Prediction	Imputation Quality[b]	Discovery U4C Meta-analysis Beta (SE)	Discovery U4C Meta-analysis *p*-value	UK Biobank Replication Beta (SE)	UK Biobank Replication *p*-value	Joint Meta-analysis Beta (SE)	Joint Meta-analysis *p*-value
RCCD1	**15q26.1**	**24**	**0.16**	**-0.11 (0.038)**	**5.8×10^{-03}**	**-0.24 (0.057)**	**2.6×10^{-05}**	**-0.15 (0.032)**	**3.6×10^{-06}**
DHODH	**16q22.2**	**7**	**0.026**	**-0.52 (0.12)**	**2.4×10^{-05}**	**-0.29 (0.15)**	**5.6×10^{-02}**	**-0.43 (0.095)**	**7.1×10^{-06}**
ANKLE1	**19p13.11**	**6**	**0.081**	**0.19 (0.093)**	**4.4×10^{-02}**	**0.43 (0.12)**	**1.9×10^{-04}**	**0.28 (0.072)**	**9.3×10^{-05}**
CASP8	2q33.1	12	0.24	-0.16 (0.042)	1.9×10^{-04}	-0.069 (0.058)	2.3×10^{-01}	-0.13 (0.034)	1.9×10^{-04}
ZNF155	19q13.31	41	0.11	0.15 (0.055)	7.6×10^{-03}	0.13 (0.069)	5.1×10^{-02}	0.14 (0.043)	9.5×10^{-04}
SLC27A2	15q21.2	43	0.063	0.15 (0.050)	2.2×10^{-03}	0.080 (0.056)	1.5×10^{-01}	0.12 (0.037)	1.2×10^{-03}
TSNARE1	8q24.3	26	0.14	0.12 (0.037)	1.2×10^{-03}	0.044 (0.058)	4.5×10^{-01}	0.098 (0.031)	1.6×10^{-03}
XRCC2	7q36.1	8	0.11	0.24 (0.084)	4.3×10^{-03}	0.14 (0.088)	1.2×10^{-01}	0.19 (0.061)	1.7×10^{-03}
DDX43	6q13	40	0.023	-0.17 (0.049)	4.5×10^{-04}	0.010 (0.10)	9.2×10^{-01}	-0.14 (0.044)	1.8×10^{-03}
WDSUB1	2q24.2	10	0.1	0.16 (0.067)	1.7×10^{-02}	0.15 (0.080)	5.8×10^{-02}	0.16 (0.052)	2.4×10^{-03}
SV2A	1q21.2	4	0.022	-1.2 (0.37)	1.7×10^{-03}	-0.38 (0.58)	5.1×10^{-01}	-0.94 (0.31)	2.7×10^{-03}
ZNF83	19q13.41	43	0.39	0.079 (0.027)	3.6×10^{-03}	0.038 (0.038)	3.2×10^{-01}	0.065 (0.022)	3.2×10^{-03}
RABGEF1	7q11.21	37	0.16	-0.11 (0.043)	1.3×10^{-02}	-0.093 (0.058)	1.1×10^{-01}	-0.1 (0.035)	3.2×10^{-03}
CAMTA2	17p13.2	17	0.022	-0.34 (0.10)	5.5×10^{-04}	-0.046 (0.12)	7.1×10^{-01}	-0.23 (0.077)	3.5×10^{-03}
STRC	15q15.3	18	0.06	-0.24 (0.086)	4.7×10^{-03}	-0.11 (0.095)	2.4×10^{-01}	-0.18 (0.064)	3.9×10^{-03}
SMAD9	13q13.3	5	0.037	-0.24 (0.098)	1.3×10^{-02}	-0.2 (0.14)	1.5×10^{-01}	-0.23 (0.080)	4.2×10^{-03}
DCAF4	14q24.2	12	0.19	0.11 (0.049)	2.2×10^{-02}	0.10 (0.061)	9.0×10^{-02}	0.11 (0.038)	4.4×10^{-03}
GYPE	4q31.21	34	0.036	0.20 (0.065)	2.6×10^{-03}	0.063 (0.084)	4.5×10^{-01}	0.15 (0.051)	4.5×10^{-03}
JAKMIP3	10q26.3	89	0.065	-0.068 (0.031)	3.1×10^{-02}	-0.078 (0.042)	6.3×10^{-02}	-0.071 (0.025)	4.5×10^{-03}
SCARF1	17p13.3	53	0.12	-0.11 (0.048)	1.8×10^{-02}	-0.091 (0.059)	1.2×10^{-01}	-0.1 (0.037)	4.9×10^{-03}
XCL2	1q24.2	18	0.056	-0.24 (0.10)	1.4×10^{-02}	-0.16 (0.11)	1.4×10^{-01}	-0.21 (0.073)	5.2×10^{-03}
SDPR	2q32.3	9	0.024	-0.28 (0.11)	1.6×10^{-02}	-0.2 (0.13)	1.4×10^{-01}	-0.24 (0.087)	5.2×10^{-03}
DVL1	1p36.33	26	0.019	0.17 (0.068)	1.1×10^{-02}	0.11 (0.086)	2.2×10^{-01}	0.15 (0.053)	5.7×10^{-03}
SMDT1	22q13.2	22	0.61	-0.051 (0.022)	2.2×10^{-02}	-0.043 (0.028)	1.2×10^{-01}	-0.048 (0.017)	5.7×10^{-03}

Gene	Location[a]	# SNPs in Prediction	Imputation Quality[b]	Discovery U4C Meta-analysis Beta (SE)	Discovery U4C Meta-analysis *p*-value	UK Biobank Replication Beta (SE)	UK Biobank Replication *p*-value	Joint Meta-analysis Beta (SE)	Joint Meta-analysis *p*-value
ZNF181	19q13.11	9	0.23	-0.11 (0.040)	4.8×10^{-03}	-0.045 (0.053)	4.0×10^{-01}	-0.088 (0.032)	5.8×10^{-03}
P2RY11	19p13.2	7	0.049	0.21 (0.074)	4.0×10^{-03}	0.067 (0.12)	5.6×10^{-01}	0.17 (0.062)	6.1×10^{-03}
PIK3C3	18q12.3	28	0.028	-0.13 (0.057)	2.3×10^{-02}	-0.14 (0.092)	1.3×10^{-01}	-0.13 (0.048)	6.2×10^{-03}
FAM170A	5q23.1	30	0.066	0.12 (0.055)	3.5×10^{-02}	0.10 (0.060)	8.5×10^{-02}	0.11 (0.041)	6.5×10^{-03}
KCTD18	2q33.1	21	0.047	-0.12 (0.058)	4.2×10^{-02}	-0.14 (0.076)	7.6×10^{-02}	-0.12 (0.046)	7.1×10^{-03}
MAN2C1	15q24.2	26	0.56	-0.067 (0.023)	4.1×10^{-03}	-0.021 (0.027)	4.3×10^{-01}	-0.047 (0.018)	7.3×10^{-03}
FAM86C1	11q13.4	27	0.21	0.086 (0.039)	2.9×10^{-02}	0.070 (0.045)	1.2×10^{-01}	0.079 (0.030)	7.7×10^{-03}
WRAP73	1p36.32	12	0.18	-0.11 (0.052)	3.6×10^{-02}	-0.096 (0.059)	1.0×10^{-01}	-0.1 (0.039)	8.0×10^{-03}
TTC18	10q22.2	36	0.13	0.058 (0.028)	3.7×10^{-02}	0.10 (0.057)	7.4×10^{-02}	0.066 (0.025)	8.0×10^{-03}
CHMP7	8p21.3	80	0.17	-0.09 (0.032)	4.4×10^{-03}	-0.022 (0.046)	6.3×10^{-01}	-0.068 (0.026)	8.8×10^{-03}
OTUB1	11q13.1	11	0.066	-0.17 (0.057)	3.1×10^{-03}	-0.011 (0.098)	9.1×10^{-01}	-0.13 (0.050)	9.1×10^{-03}
TRPC4AP	20q11.22	31	0.04	0.15 (0.049)	2.4×10^{-03}	0.012 (0.070)	8.6×10^{-01}	0.10 (0.040)	9.5×10^{-03}
WBSCR27	7q11.23	29	0.62	0.055 (0.019)	3.1×10^{-03}	0.0070 (0.027)	8.0×10^{-01}	0.040 (0.015)	9.8×10^{-03}
NELFE	6p21.33	14	0.032	-0.19 (0.077)	1.5×10^{-02}	-0.11 (0.11)	3.1×10^{-01}	-0.16 (0.064)	1.0×10^{-02}
TMEM185B	2q14.2	23	0.097	0.12 (0.054)	2.8×10^{-02}	0.091 (0.067)	1.7×10^{-01}	0.11 (0.042)	1.0×10^{-02}
POLDIP3	22q13.2	3	0.032	3.2 (1.6)	4.4×10^{-02}	3.6 (2.3)	1.1×10^{-01}	3.3 (1.3)	1.0×10^{-02}
TECPR1	7q21.3	1	0.02	-1.5 (0.55)	5.6×10^{-03}	-0.4 (0.70)	5.6×10^{-01}	-1.1 (0.43)	1.1×10^{-02}
KIFC2	8q24.3	19	0.055	0.14 (0.062)	2.0×10^{-02}	0.091 (0.078)	2.4×10^{-01}	0.12 (0.048)	1.1×10^{-02}
FOXN1	17q11.2	4	0.033	-0.29 (0.12)	2.1×10^{-02}	-0.16 (0.13)	2.3×10^{-01}	-0.22 (0.090)	1.2×10^{-02}
LILRB3	19q13.42	71	0.032	-0.12 (0.040)	2.5×10^{-03}	-0.012 (0.047)	8.0×10^{-01}	-0.075 (0.030)	1.3×10^{-02}
LY6G5C	6p21.33	27	0.054	-0.13 (0.060)	2.9×10^{-02}	-0.089 (0.069)	2.0×10^{-01}	-0.11 (0.046)	1.3×10^{-02}
CBX8	17q25.3	10	0.068	0.25 (0.082)	2.4×10^{-03}	0.019 (0.098)	8.4×10^{-01}	0.15 (0.063)	1.4×10^{-02}
CCDC114	19q13.33	14	0.096	0.24 (0.079)	2.5×10^{-03}	0.019 (0.096)	8.4×10^{-01}	0.15 (0.061)	1.4×10^{-02}
GPR19	12p13.1	19	0.054	-0.16 (0.069)	2.0×10^{-02}	-0.093 (0.093)	3.2×10^{-01}	-0.14 (0.055)	1.4×10^{-02}
STRA13	17q25.3	10	0.25	-0.084 (0.040)	3.5×10^{-02}	-0.061 (0.047)	1.9×10^{-01}	-0.074 (0.030)	1.4×10^{-02}
ITCH	20q11.22	21	0.045	0.31 (0.086)	2.8×10^{-04}	-0.024 (0.090)	7.9×10^{-01}	0.15 (0.062)	1.5×10^{-02}
GLYATL2	11q12.1	74	0.089	0.13 (0.040)	1.9×10^{-03}	-0.024 (0.064)	7.1×10^{-01}	0.083 (0.034)	1.5×10^{-02}

Gene	Location[a]	# SNPs in Prediction	Imputation Quality[b]	Discovery U4C Meta-analysis Beta (SE)	Discovery U4C Meta-analysis *p*-value	UK Biobank Replication Beta (SE)	UK Biobank Replication *p*-value	Joint Meta-analysis Beta (SE)	Joint Meta-analysis *p*-value
CCDC53	12q23.2	13	0.098	0.14 (0.069)	$4.0x10^{-02}$	0.14 (0.11)	$1.9x10^{-01}$	0.14 (0.058)	$1.5x10^{-02}$
FRG1B	20q11.21	32	0.062	-0.11 (0.042)	$7.6x10^{-03}$	-0.028 (0.049)	$5.6x10^{-01}$	-0.076 (0.032)	$1.6x10^{-02}$
JAKMIP2	5q32	30	0.059	0.14 (0.055)	$8.9x10^{-03}$	0.039 (0.071)	$5.8x10^{-01}$	0.10 (0.043)	$1.6x10^{-02}$
DNAH1	3p21.1	58	0.045	0.072 (0.036)	$4.7x10^{-02}$	0.064 (0.046)	$1.7x10^{-01}$	0.069 (0.029)	$1.6x10^{-02}$
IL1RN	2q13	15	0.068	0.16 (0.071)	$2.9x10^{-02}$	0.10 (0.094)	$2.8x10^{-01}$	0.14 (0.057)	$1.7x10^{-02}$
GTF2H1	11p15.1	12	0.17	-0.11 (0.056)	$4.3x10^{-02}$	-0.086 (0.065)	$1.9x10^{-01}$	-0.1 (0.043)	$1.7x10^{-02}$
DCAF5	14q24.1	31	0.034	0.13 (0.064)	$4.9x10^{-02}$	0.12 (0.088)	$1.9x10^{-01}$	0.12 (0.052)	$1.8x10^{-02}$
STEAP3	2q14.2	18	0.094	0.17 (0.060)	$4.9x10^{-03}$	0.015 (0.077)	$8.5x10^{-01}$	0.11 (0.047)	$2.0x10^{-02}$
LRRK1	15q26.3	30	0.034	0.15 (0.055)	$5.6x10^{-03}$	-0.043 (0.11)	$7.0x10^{-01}$	0.11 (0.049)	$2.0x10^{-02}$
AP3M2	8p11.21	16	0.095	-0.12 (0.053)	$2.2x10^{-02}$	-0.056 (0.067)	$4.0x10^{-01}$	-0.096 (0.041)	$2.0x10^{-02}$
ZSCAN29	15q15.3	33	0.24	0.11 (0.049)	$2.2x10^{-02}$	0.050 (0.050)	$3.1x10^{-01}$	0.082 (0.035)	$2.0x10^{-02}$
TACSTD2	1p32.1	11	0.038	0.21 (0.10)	$4.1x10^{-02}$	0.15 (0.12)	$2.3x10^{-01}$	0.18 (0.079)	$2.0x10^{-02}$
PHOSPHO2	2q31.1	10	0.14	-0.12 (0.059)	$4.3x10^{-02}$	-0.086 (0.072)	$2.3x10^{-01}$	-0.11 (0.046)	$2.0x10^{-02}$
WIZ	19p13.12	17	0.022	0.34 (0.10)	$7.9x10^{-04}$	-0.05 (0.12)	$6.8x10^{-01}$	0.18 (0.078)	$2.1x10^{-02}$
PAN3	13q12.2	27	0.023	-0.21 (0.076)	$5.7x10^{-03}$	-0.012 (0.10)	$9.1x10^{-01}$	-0.14 (0.061)	$2.1x10^{-02}$
CTD-2054N24.2	15q26.3	23	0.22	-0.094 (0.041)	$2.1x10^{-02}$	-0.041 (0.047)	$3.9x10^{-01}$	-0.071 (0.031)	$2.1x10^{-02}$
BIRC5	17q25.3	21	0.084	0.12 (0.055)	$3.3x10^{-02}$	0.083 (0.085)	$3.3x10^{-01}$	0.11 (0.046)	$2.1x10^{-02}$
OXTR	3p25.3	13	0.042	0.22 (0.11)	$4.2x10^{-02}$	0.13 (0.11)	$2.2x10^{-01}$	0.17 (0.075)	$2.1x10^{-02}$
CNOT6L	4q21.1	67	0.11	-0.11 (0.042)	$6.0x10^{-03}$	-0.0093 (0.055)	$8.7x10^{-01}$	-0.076 (0.033)	$2.2x10^{-02}$
LDB3	10q23.2	20	0.18	0.095 (0.036)	$8.1x10^{-03}$	0.0059 (0.056)	$9.2x10^{-01}$	0.069 (0.030)	$2.2x10^{-02}$
CKAP5	11p11.2	9	0.039	0.18 (0.085)	$2.9x10^{-02}$	0.10 (0.12)	$3.8x10^{-01}$	0.16 (0.069)	$2.2x10^{-02}$
CUL9	6p21.1	63	0.077	0.068 (0.035)	$4.9x10^{-02}$	0.059 (0.050)	$2.4x10^{-01}$	0.065 (0.028)	$2.2x10^{-02}$
RIF1	2q23.3	35	0.16	0.11 (0.045)	$1.3x10^{-02}$	0.029 (0.059)	$6.3x10^{-01}$	0.081 (0.036)	$2.3x10^{-02}$
OOEP	6q13	33	0.037	-0.12 (0.059)	$4.1x10^{-02}$	-0.092 (0.087)	$2.9x10^{-01}$	-0.11 (0.049)	$2.3x10^{-02}$
IL10RB	21q22.11	9	0.085	0.18 (0.078)	$1.9x10^{-02}$	0.065 (0.11)	$5.4x10^{-01}$	0.14 (0.063)	$2.4x10^{-02}$
CARHSP1	16p13.2	35	0.077	-0.11 (0.051)	$2.7x10^{-02}$	-0.056 (0.073)	$4.4x10^{-01}$	-0.094 (0.042)	$2.4x10^{-02}$
ZNF112	19q13.31	24	0.025	-0.13 (0.062)	$3.5x10^{-02}$	-0.078 (0.085)	$3.6x10^{-01}$	-0.11 (0.050)	$2.4x10^{-02}$

Gene	Location[a]	# SNPs in Prediction	Imputation Quality[b]	Discovery U4C Meta-analysis Beta (SE)	Discovery U4C Meta-analysis *p*-value	UK Biobank Replication Beta (SE)	UK Biobank Replication *p*-value	Joint Meta-analysis Beta (SE)	Joint Meta-analysis *p*-value
TXNL4B	16q22.2	18	0.033	0.11 (0.052)	$3.6x10^{-02}$	0.11 (0.13)	$4.0x10^{-01}$	0.11 (0.048)	$2.4x10^{-02}$
MET	7q31.2	81	0.071	0.072 (0.030)	$1.8x10^{-02}$	0.022 (0.042)	$6.0x10^{-01}$	0.055 (0.025)	$2.5x10^{-02}$
ZNF544	19q13.43	10	0.022	-0.21 (0.11)	$4.7x10^{-02}$	-0.13 (0.11)	$2.4x10^{-01}$	-0.17 (0.077)	$2.5x10^{-02}$
PCDHA10	5q31.3	10	0.12	0.12 (0.054)	$2.8x10^{-02}$	0.052 (0.058)	$3.7x10^{-01}$	0.088 (0.040)	$2.6x10^{-02}$
TREM1	6p21.1	26	0.025	0.17 (0.077)	$3.2x10^{-02}$	0.091 (0.11)	$4.2x10^{-01}$	0.14 (0.064)	$2.6x10^{-02}$
CCNF	16p13.3	15	0.022	0.21 (0.11)	$4.8x10^{-02}$	0.14 (0.13)	$2.7x10^{-01}$	0.18 (0.083)	$2.6x10^{-02}$
PRR12	19q13.33	6	0.12	0.19 (0.073)	$9.8x10^{-03}$	0.029 (0.090)	$7.4x10^{-01}$	0.13 (0.057)	$2.7x10^{-02}$
ZNF606	19q13.43	2	0.027	1.1 (0.57)	$4.3x10^{-02}$	1.1 (1.2)	$3.6x10^{-01}$	1.1 (0.51)	$2.7x10^{-02}$
Z98049.1	6q27	2	0.034	0.32 (0.14)	$2.2x10^{-02}$	0.11 (0.20)	$5.6x10^{-01}$	0.25 (0.11)	$2.8x10^{-02}$
GABRB3	15q12	12	0.19	-0.11 (0.051)	$3.7x10^{-02}$	-0.058 (0.063)	$3.6x10^{-01}$	-0.087 (0.040)	$2.8x10^{-02}$
MPP4	2q33.1	44	0.03	-0.13 (0.047)	$6.5x10^{-03}$	-0.0077 (0.057)	$8.9x10^{-01}$	-0.079 (0.036)	$2.9x10^{-02}$
MYO15B	17q25.1	22	0.11	0.14 (0.054)	$1.0x10^{-02}$	0.018 (0.070)	$8.0x10^{-01}$	0.094 (0.043)	$2.9x10^{-02}$
TTL	2q13	62	0.037	-0.097 (0.049)	$4.8x10^{-02}$	-0.066 (0.066)	$3.2x10^{-01}$	-0.086 (0.039)	$2.9x10^{-02}$
RNMT	18p11.21	25	0.028	0.16 (0.077)	$3.9x10^{-02}$	0.096 (0.12)	$4.0x10^{-01}$	0.14 (0.064)	$3.0x10^{-02}$
GLRB	4q32.1	23	0.031	-0.14 (0.067)	$4.2x10^{-02}$	-0.085 (0.099)	$3.9x10^{-01}$	-0.12 (0.056)	$3.0x10^{-02}$
CYP27C1	2q14.3	13	0.089	-0.15 (0.067)	$2.7x10^{-02}$	-0.057 (0.095)	$5.5x10^{-01}$	-0.12 (0.055)	$3.1x10^{-02}$
GAPT	5q11.2	31	0.026	-0.16 (0.078)	$4.3x10^{-02}$	-0.091 (0.10)	$3.7x10^{-01}$	-0.13 (0.062)	$3.1x10^{-02}$
STXBP4	17q22	43	0.091	0.14 (0.050)	$4.6x10^{-03}$	-0.0047 (0.061)	$9.4x10^{-01}$	0.083 (0.038)	$3.2x10^{-02}$
ALS2CR12	2q33.1	30	0.067	0.14 (0.065)	$3.7x10^{-02}$	0.069 (0.085)	$4.2x10^{-01}$	0.11 (0.052)	$3.2x10^{-02}$
DNAH6	2p11.2	7	0.23	-0.16 (0.054)	$2.6x10^{-03}$	0.0073 (0.058)	$9.0x10^{-01}$	-0.084 (0.039)	$3.3x10^{-02}$
IFT46	11q23.3	22	0.25	-0.086 (0.034)	$1.1x10^{-02}$	0.0048 (0.056)	$9.3x10^{-01}$	-0.062 (0.029)	$3.3x10^{-02}$
MIS18BP1	14q21.2	23	0.086	0.086 (0.041)	$3.6x10^{-02}$	0.046 (0.074)	$5.3x10^{-01}$	0.077 (0.036)	$3.3x10^{-02}$
CXCL9	4q21.1	3	0.027	-1.3 (0.66)	$4.6x10^{-02}$	-0.86 (1.1)	$4.2x10^{-01}$	-1.2 (0.56)	$3.4x10^{-02}$
ATF3	1q32.3	26	0.033	-0.24 (0.095)	$1.3x10^{-02}$	-0.043 (0.10)	$6.8x10^{-01}$	-0.15 (0.070)	$3.5x10^{-02}$
NT5C3B	17q21.2	20	0.58	0.047 (0.021)	$2.5x10^{-02}$	0.015 (0.026)	$5.7x10^{-01}$	0.034 (0.016)	$3.6x10^{-02}$
CYP2J2	1p32.1	14	0.097	-0.19 (0.086)	$2.8x10^{-02}$	-0.065 (0.090)	$4.7x10^{-01}$	-0.13 (0.062)	$3.6x10^{-02}$
MCHR1	22q13.2	15	0.048	-0.13 (0.066)	$4.8x10^{-02}$	-0.078 (0.094)	$4.1x10^{-01}$	-0.11 (0.054)	$3.6x10^{-02}$

Gene	Location[a]	# SNPs in Prediction	Imputation Quality[b]	Discovery U4C Meta-analysis		UK Biobank Replication		Joint Meta-analysis	
				Beta (SE)	*p*-value	Beta (SE)	*p*-value	Beta (SE)	*p*-value
RPS10	6p21.31	8	0.057	0.14 (0.061)	$2.1x10^{-02}$	0.012 (0.11)	$9.2x10^{-01}$	0.11 (0.054)	$3.7x10^{-02}$
ZNF500	16p13.3	65	0.28	-0.062 (0.027)	$2.4x10^{-02}$	-0.017 (0.035)	$6.3x10^{-01}$	-0.045 (0.022)	$3.8x10^{-02}$
KIAA1279	10q22.1	12	0.039	-0.31 (0.11)	$4.4x10^{-03}$	0.0073 (0.12)	$9.5x10^{-01}$	-0.17 (0.081)	$3.9x10^{-02}$
MVB12B	9q33.3	16	0.022	-0.16 (0.082)	$4.4x10^{-02}$	-0.08 (0.10)	$4.3x10^{-01}$	-0.13 (0.064)	$3.9x10^{-02}$
XRN2	20p11.23	90	0.071	0.069 (0.029)	$1.7x10^{-02}$	0.0073 (0.041)	$8.6x10^{-01}$	0.049 (0.024)	$4.0x10^{-02}$
LRRC27	10q26.3	8	0.12	0.11 (0.049)	$2.8x10^{-02}$	0.028 (0.079)	$7.2x10^{-01}$	0.086 (0.042)	$4.0x10^{-02}$
SLC5A11	16p12.1	35	0.44	-0.056 (0.026)	$3.1x10^{-02}$	-0.019 (0.030)	$5.2x10^{-01}$	-0.04 (0.020)	$4.0x10^{-02}$
NDUFA10	2q37.3	61	0.44	0.050 (0.024)	$3.4x10^{-02}$	0.019 (0.027)	$4.9x10^{-01}$	0.037 (0.018)	$4.0x10^{-02}$
IGSF6	16p12.2	22	0.073	-0.14 (0.065)	$3.4x10^{-02}$	-0.049 (0.11)	$6.5x10^{-01}$	-0.11 (0.055)	$4.1x10^{-02}$
FLCN	17p11.2	34	0.54	0.043 (0.021)	$4.1x10^{-02}$	0.019 (0.028)	$5.0x10^{-01}$	0.035 (0.017)	$4.1x10^{-02}$
CHRM1	11q12.3	27	0.039	-0.12 (0.053)	$2.9x10^{-02}$	-0.032 (0.077)	$6.8x10^{-01}$	-0.089 (0.044)	$4.2x10^{-02}$
ZBTB12	6p21.33	8	0.027	-0.28 (0.093)	$2.7x10^{-03}$	0.29 (0.19)	$1.3x10^{-01}$	-0.17 (0.084)	$4.4x10^{-02}$
C2CD4C	19p13.3	11	0.15	0.16 (0.063)	$1.1x10^{-02}$	0.012 (0.070)	$8.6x10^{-01}$	0.094 (0.047)	$4.4x10^{-02}$
CABLES2	20q13.33	13	0.1	-0.18 (0.075)	$1.6x10^{-02}$	-0.032 (0.077)	$6.8x10^{-01}$	-0.11 (0.054)	$4.4x10^{-02}$
AP000769.1	11q13.1	9	0.035	0.22 (0.097)	$2.2x10^{-02}$	0.035 (0.14)	$8.0x10^{-01}$	0.16 (0.079)	$4.4x10^{-02}$
CCL23	17q12	17	0.26	-0.066 (0.032)	$3.9x10^{-02}$	-0.024 (0.046)	$5.9x10^{-01}$	-0.052 (0.026)	$4.6x10^{-02}$
MAPK8IP2	22q13.33	13	0.076	0.19 (0.069)	$6.7x10^{-03}$	-0.011 (0.082)	$9.0x10^{-01}$	0.10 (0.053)	$4.7x10^{-02}$
ABCA5	17q24.3	2	0.033	3.3 (1.4)	$2.0x10^{-02}$	0.46 (1.8)	$8.0x10^{-01}$	2.2 (1.1)	$4.8x10^{-02}$
GABRP	5q35.1	9	0.029	0.28 (0.12)	$2.5x10^{-02}$	0.050 (0.17)	$7.7x10^{-01}$	0.20 (0.10)	$4.8x10^{-02}$
GLTP	12q24.11	10	0.093	-0.16 (0.064)	$1.0x10^{-02}$	0.0034 (0.078)	$9.7x10^{-01}$	-0.097 (0.049)	$4.9x10^{-02}$
CRHR2	7p14.3	25	0.42	-0.062 (0.029)	$3.4x10^{-02}$	-0.019 (0.037)	$6.1x10^{-01}$	-0.045 (0.023)	$4.9x10^{-02}$
TCTN2	12q24.31	36	0.26	-0.069 (0.033)	$3.7x10^{-02}$	-0.021 (0.048)	$6.6x10^{-01}$	-0.054 (0.027)	$5.0x10^{-02}$
CYP4A22	1p33	18	0.019	-0.21 (0.076)	$4.5x10^{-03}$	0.064 (0.10)	$5.5x10^{-01}$	-0.12 (0.061)	$5.1x10^{-02}$
JMJD1C	10q21.3	1	0.041	5.1 (2.4)	$3.3x10^{-02}$	1.5 (2.2)	$4.9x10^{-01}$	3.2 (1.6)	$5.1x10^{-02}$
AAAS	12q13.13	36	0.043	0.10 (0.052)	$4.3x10^{-02}$	0.022 (0.14)	$8.7x10^{-01}$	0.094 (0.048)	$5.1x10^{-02}$
RHNO1	12p13.33	13	0.052	-0.18 (0.088)	$3.8x10^{-02}$	-0.054 (0.12)	$6.6x10^{-01}$	-0.14 (0.071)	$5.2x10^{-02}$
WDR81	17p13.3	11	0.034	-0.32 (0.10)	$1.3x10^{-03}$	0.14 (0.13)	$2.9x10^{-01}$	-0.16 (0.081)	$5.3x10^{-02}$

Gene	Location[a]	# SNPs in Prediction	Imputation Quality[b]	Discovery U4C Meta-analysis Beta (SE)	Discovery U4C Meta-analysis *p*-value	UK Biobank Replication Beta (SE)	UK Biobank Replication *p*-value	Joint Meta-analysis Beta (SE)	Joint Meta-analysis *p*-value
TAS2R43	12p13.2	21	0.29	-0.085 (0.036)	$1.7x10^{-02}$	-0.0066 (0.043)	$8.8x10^{-01}$	-0.053 (0.027)	$5.3x10^{-02}$
SH3BGR	21q22.2	12	0.078	-0.18 (0.090)	$4.3x10^{-02}$	-0.065 (0.098)	$5.1x10^{-01}$	-0.13 (0.066)	$5.3x10^{-02}$
NOMO3	16p13.11	36	0.17	-0.098 (0.048)	$4.2x10^{-02}$	-0.033 (0.053)	$5.3x10^{-01}$	-0.069 (0.036)	$5.4x10^{-02}$
KIF11	10q23.33	1	0.055	4.9 (2.2)	$2.5x10^{-02}$	1.00 (2.3)	$6.6x10^{-01}$	3.0 (1.6)	$5.5x10^{-02}$
PLCG2	16q23.3	18	0.044	0.17 (0.077)	$3.1x10^{-02}$	0.034 (0.098)	$7.3x10^{-01}$	0.12 (0.060)	$5.6x10^{-02}$
NDUFB1	14q32.12	16	0.041	-0.22 (0.092)	$1.5x10^{-02}$	0.0044 (0.12)	$9.7x10^{-01}$	-0.14 (0.072)	$5.7x10^{-02}$
MDGA1	6p21.2	65	0.3	-0.063 (0.029)	$3.0x10^{-02}$	-0.012 (0.037)	$7.5x10^{-01}$	-0.044 (0.023)	$5.7x10^{-02}$
MAGEF1	3q27.1	38	0.088	0.093 (0.045)	$3.6x10^{-02}$	0.022 (0.061)	$7.2x10^{-01}$	0.068 (0.036)	$5.7x10^{-02}$
ADRB1	10q25.3	17	0.021	-0.16 (0.078)	$3.8x10^{-02}$	-0.035 (0.12)	$7.7x10^{-01}$	-0.12 (0.065)	$5.9x10^{-02}$
WDR25	14q32.2	15	0.047	-0.13 (0.063)	$3.9x10^{-02}$	-0.035 (0.080)	$6.6x10^{-01}$	-0.094 (0.050)	$5.9x10^{-02}$
RPA1	17p13.3	95	0.098	-0.056 (0.027)	$3.8x10^{-02}$	-0.013 (0.037)	$7.3x10^{-01}$	-0.041 (0.022)	$6.0x10^{-02}$
HLA-DOB	6p21.32	43	0.48	-0.051 (0.024)	$3.4x10^{-02}$	-0.01 (0.030)	$7.3x10^{-01}$	-0.035 (0.019)	$6.1x10^{-02}$
DHX16	6p21.33	17	0.025	-0.13 (0.066)	$4.3x10^{-02}$	-0.037 (0.087)	$6.7x10^{-01}$	-0.098 (0.052)	$6.1x10^{-02}$
MYLK4	6p25.2	31	0.26	0.095 (0.038)	$1.3x10^{-02}$	-0.0033 (0.047)	$9.4x10^{-01}$	0.055 (0.030)	$6.2x10^{-02}$
MSMO1	4q32.3	8	0.045	0.20 (0.090)	$2.2x10^{-02}$	0.013 (0.11)	$9.1x10^{-01}$	0.13 (0.071)	$6.2x10^{-02}$
PSMG1	21q22.2	26	0.36	0.077 (0.036)	$3.2x10^{-02}$	0.016 (0.041)	$7.0x10^{-01}$	0.050 (0.027)	$6.3x10^{-02}$
STRADA	17q23.3	47	0.052	0.13 (0.051)	$8.9x10^{-03}$	-0.011 (0.058)	$8.5x10^{-01}$	0.070 (0.038)	$6.5x10^{-02}$
PTER	10p13	81	0.085	0.069 (0.032)	$3.1x10^{-02}$	0.0073 (0.044)	$8.7x10^{-01}$	0.048 (0.026)	$6.5x10^{-02}$
AC022498.1	3q27.3	10	0.044	-0.15 (0.074)	$3.9x10^{-02}$	-0.03 (0.10)	$7.8x10^{-01}$	-0.11 (0.061)	$6.5x10^{-02}$
SYNGR3	16p13.3	47	0.027	0.13 (0.049)	$8.2x10^{-03}$	-0.032 (0.066)	$6.3x10^{-01}$	0.072 (0.039)	$6.6x10^{-02}$
LSR	19q13.12	21	0.21	0.12 (0.049)	$1.1x10^{-02}$	0.0027 (0.049)	$9.6x10^{-01}$	0.063 (0.035)	$6.6x10^{-02}$
SNTA1	20q11.21	23	0.026	0.22 (0.076)	$4.5x10^{-03}$	-0.043 (0.087)	$6.2x10^{-01}$	0.10 (0.057)	$6.8x10^{-02}$
IPO8	12p11.21	13	0.1	-0.22 (0.081)	$7.0x10^{-03}$	0.034 (0.096)	$7.2x10^{-01}$	-0.11 (0.062)	$6.8x10^{-02}$
UROD	1p34.1	25	0.14	-0.11 (0.049)	$2.0x10^{-02}$	-0.0011 (0.060)	$9.9x10^{-01}$	-0.069 (0.038)	$6.8x10^{-02}$
SULT2B1	19q13.33	35	0.036	0.13 (0.060)	$3.3x10^{-02}$	0.018 (0.078)	$8.1x10^{-01}$	0.087 (0.048)	$6.8x10^{-02}$
FKBP7	2q31.2	6	0.064	0.23 (0.079)	$3.8x10^{-03}$	-0.12 (0.12)	$3.1x10^{-01}$	0.12 (0.065)	$6.9x10^{-02}$
EBNA1BP2	1p34.2	22	0.019	-0.2 (0.081)	$1.1x10^{-02}$	0.041 (0.11)	$7.0x10^{-01}$	-0.12 (0.065)	$7.1x10^{-02}$

Gene	Location[a]	# SNPs in Prediction	Imputation Quality[b]	Discovery U4C Meta-analysis Beta (SE)	Discovery U4C Meta-analysis *p*-value	UK Biobank Replication Beta (SE)	UK Biobank Replication *p*-value	Joint Meta-analysis Beta (SE)	Joint Meta-analysis *p*-value
SETD9	5q11.2	21	0.27	0.073 (0.030)	$1.7x10^{-02}$	-0.015 (0.046)	$7.4x10^{-01}$	0.046 (0.025)	$7.1x10^{-02}$
EVI5	1p22.1	24	0.073	-0.24 (0.10)	$1.9x10^{-02}$	-0.025 (0.096)	$8.0x10^{-01}$	-0.13 (0.070)	$7.1x10^{-02}$
TCP11	6p21.31	8	0.042	0.24 (0.091)	$9.4x10^{-03}$	-0.038 (0.11)	$7.3x10^{-01}$	0.13 (0.070)	$7.2x10^{-02}$
WDR20	14q32.31	53	0.028	-0.094 (0.040)	$1.8x10^{-02}$	0.016 (0.058)	$7.8x10^{-01}$	-0.059 (0.033)	$7.3x10^{-02}$
ABHD8	19p13.11	11	0.092	0.17 (0.079)	$3.3x10^{-02}$	0.027 (0.090)	$7.6x10^{-01}$	0.11 (0.059)	$7.3x10^{-02}$
CBX1	17q21.32	17	0.019	-0.15 (0.076)	$4.4x10^{-02}$	-0.03 (0.10)	$7.6x10^{-01}$	-0.11 (0.060)	$7.3x10^{-02}$
SLC25A44	1q22	24	0.12	-0.19 (0.076)	$1.5x10^{-02}$	-0.00025 (0.082)	1.00E+00	-0.1 (0.056)	$7.5x10^{-02}$
TRAIP	3p21.31	2	0.037	-2.3 (0.97)	$1.7x10^{-02}$	-0.25 (0.85)	$7.7x10^{-01}$	-1.1 (0.64)	$7.5x10^{-02}$
TXNL4A	18q23	11	0.067	0.17 (0.081)	$4.1x10^{-02}$	0.022 (0.11)	$8.5x10^{-01}$	0.12 (0.066)	$7.5x10^{-02}$
MS4A14	11q12.2	12	0.28	0.088 (0.036)	$1.5x10^{-02}$	-0.01 (0.046)	$8.2x10^{-01}$	0.051 (0.029)	$7.6x10^{-02}$
NNAT	20q11.23	24	0.048	-0.14 (0.058)	$2.0x10^{-02}$	0.031 (0.090)	$7.3x10^{-01}$	-0.087 (0.049)	$7.6x10^{-02}$
WDR11	10q26.12	25	0.45	0.047 (0.023)	$4.1x10^{-02}$	0.0060 (0.032)	$8.5x10^{-01}$	0.033 (0.019)	$7.6x10^{-02}$
IMPDH1	7q32.1	9	0.086	0.15 (0.063)	$1.6x10^{-02}$	-0.026 (0.085)	$7.6x10^{-01}$	0.088 (0.051)	$8.0x10^{-02}$
ANKS6	9q22.33	12	0.024	-0.21 (0.10)	$4.2x10^{-02}$	-0.016 (0.16)	$9.2x10^{-01}$	-0.15 (0.086)	$8.0x10^{-02}$
TAS2R10	12p13.2	9	0.021	0.31 (0.12)	$1.2x10^{-02}$	-0.017 (0.13)	$8.9x10^{-01}$	0.15 (0.089)	$8.1x10^{-02}$
DHRS3	1p36.22	36	0.054	0.16 (0.069)	$1.9x10^{-02}$	0.0025 (0.074)	$9.7x10^{-01}$	0.088 (0.050)	$8.1x10^{-02}$
SLFN12L	17q12	6	0.03	-0.21 (0.11)	$4.2x10^{-02}$	-0.034 (0.13)	$7.9x10^{-01}$	-0.14 (0.081)	$8.3x10^{-02}$
ZNF350	19q13.41	33	0.042	-0.1 (0.052)	$4.7x10^{-02}$	-0.017 (0.070)	$8.1x10^{-01}$	-0.072 (0.042)	$8.3x10^{-02}$
C17orf62	17q25.3	2	0.067	-0.16 (0.081)	$4.4x10^{-02}$	-0.0035 (0.13)	$9.8x10^{-01}$	-0.12 (0.069)	$8.4x10^{-02}$
MARVELD1	10q24.2	27	0.029	-0.17 (0.082)	$3.9x10^{-02}$	-0.01 (0.11)	$9.2x10^{-01}$	-0.11 (0.066)	$8.8x10^{-02}$
MMP16	8q21.3	24	0.02	0.16 (0.064)	$1.4x10^{-02}$	-0.041 (0.086)	$6.3x10^{-01}$	0.087 (0.051)	$9.1x10^{-02}$
GPATCH4	1q23.1	3	0.019	0.69 (0.34)	$4.7x10^{-02}$	0.063 (0.46)	$8.9x10^{-01}$	0.46 (0.28)	$9.4x10^{-02}$
ABHD11	7q11.23	17	0.026	-0.19 (0.093)	$4.1x10^{-02}$	-0.017 (0.11)	$8.8x10^{-01}$	-0.12 (0.071)	$9.6x10^{-02}$
RPL9	4p14	6	0.68	-0.043 (0.021)	$4.0x10^{-02}$	0.020 (0.042)	$6.3x10^{-01}$	-0.03 (0.019)	$1.0x10^{-01}$
MTMR4	17q22	4	0.034	-0.22 (0.11)	$4.2x10^{-02}$	-0.017 (0.13)	$8.9x10^{-01}$	-0.13 (0.081)	$1.0x10^{-01}$
C1orf229	1q44	11	0.028	0.18 (0.083)	$2.8x10^{-02}$	-0.027 (0.11)	$8.0x10^{-01}$	0.10 (0.066)	$1.1x10^{-01}$
ACTR3C	7q36.1	12	0.054	0.16 (0.076)	$3.4x10^{-02}$	-0.031 (0.11)	$7.8x10^{-01}$	0.10 (0.063)	$1.1x10^{-01}$

Gene	Location[a]	# SNPs in Prediction	Imputation Quality[b]	Discovery U4C Meta-analysis Beta (SE)	Discovery U4C Meta-analysis *p*-value	UK Biobank Replication Beta (SE)	UK Biobank Replication *p*-value	Joint Meta-analysis Beta (SE)	Joint Meta-analysis *p*-value
HP1BP3	1p36.12	33	0.02	0.17 (0.072)	$1.8x10^{-02}$	-0.044 (0.091)	$6.3x10^{-01}$	0.088 (0.056)	$1.2x10^{-01}$
RPS8	1p34.1	13	0.061	-0.16 (0.072)	$2.3x10^{-02}$	0.045 (0.099)	$6.5x10^{-01}$	-0.092 (0.058)	$1.2x10^{-01}$
COMMD7	20q11.21	23	0.036	0.17 (0.077)	$2.4x10^{-02}$	-0.0066 (0.080)	$9.3x10^{-01}$	0.087 (0.056)	$1.2x10^{-01}$
PPOX	1q23.3	112	0.094	-0.057 (0.025)	$2.5x10^{-02}$	0.018 (0.036)	$6.2x10^{-01}$	-0.032 (0.021)	$1.2x10^{-01}$
RPN1	3q21.3	24	0.044	-0.13 (0.058)	$2.6x10^{-02}$	0.023 (0.074)	$7.6x10^{-01}$	-0.071 (0.045)	$1.2x10^{-01}$
EPB41L4A	5q22.2	20	0.12	-0.17 (0.076)	$2.6x10^{-02}$	0.0013 (0.075)	$9.9x10^{-01}$	-0.083 (0.053)	$1.2x10^{-01}$
TAP1	6p21.32	9	0.021	0.24 (0.10)	$2.0x10^{-02}$	-0.13 (0.16)	$4.1x10^{-01}$	0.13 (0.087)	$1.3x10^{-01}$
TMEM201	1p36.22	27	0.087	-0.091 (0.041)	$2.7x10^{-02}$	0.038 (0.061)	$5.4x10^{-01}$	-0.051 (0.034)	$1.3x10^{-01}$
AGAP8	10q11.23	29	0.54	0.051 (0.025)	$4.1x10^{-02}$	0.00011 (0.028)	1.00E+00	0.028 (0.019)	$1.3x10^{-01}$
HMGB1	13q12.3	17	0.18	-0.071 (0.035)	$4.5x10^{-02}$	0.13 (0.11)	$2.1x10^{-01}$	-0.05 (0.034)	$1.3x10^{-01}$
CHKB	22q13.33	15	0.052	-0.22 (0.082)	$7.0x10^{-03}$	0.061 (0.087)	$4.8x10^{-01}$	-0.088 (0.059)	$1.4x10^{-01}$
MAP1LC3A	20q11.22	22	0.18	-0.09 (0.040)	$2.6x10^{-02}$	0.019 (0.049)	$7.0x10^{-01}$	-0.045 (0.031)	$1.4x10^{-01}$
NDUFAF7	2p22.2	11	0.029	0.27 (0.13)	$3.5x10^{-02}$	-0.031 (0.15)	$8.4x10^{-01}$	0.14 (0.097)	$1.4x10^{-01}$
MOGS	2p13.1	11	0.076	0.20 (0.070)	$4.8x10^{-03}$	-0.18 (0.11)	$8.6x10^{-02}$	0.084 (0.058)	$1.5x10^{-01}$
ZBTB25	14q23.3	32	0.079	0.14 (0.056)	$1.2x10^{-02}$	-0.066 (0.073)	$3.6x10^{-01}$	0.064 (0.044)	$1.5x10^{-01}$
CLN8	8p23.3	17	0.11	0.18 (0.073)	$1.5x10^{-02}$	-0.033 (0.075)	$6.6x10^{-01}$	0.075 (0.052)	$1.5x10^{-01}$
PLEKHA1	10q26.13	7	0.046	0.32 (0.13)	$1.6x10^{-02}$	-0.1 (0.16)	$5.3x10^{-01}$	0.15 (0.10)	$1.5x10^{-01}$
SYT17	16p12.3	19	0.031	0.20 (0.085)	$1.7x10^{-02}$	-0.15 (0.13)	$2.8x10^{-01}$	0.10 (0.072)	$1.5x10^{-01}$
ZNF302	19q13.11	47	0.29	0.081 (0.040)	$4.1x10^{-02}$	-0.0057 (0.045)	$9.0x10^{-01}$	0.044 (0.030)	$1.5x10^{-01}$
MAPRE3	2p23.3	8	0.021	-0.25 (0.11)	$2.7x10^{-02}$	0.12 (0.16)	$4.6x10^{-01}$	-0.13 (0.092)	$1.6x10^{-01}$
SPEF2	5p13.2	73	0.033	-0.077 (0.035)	$2.8x10^{-02}$	0.043 (0.054)	$4.2x10^{-01}$	-0.041 (0.029)	$1.6x10^{-01}$
PPME1	11q13.4	37	0.041	0.14 (0.062)	$2.0x10^{-02}$	-0.042 (0.072)	$5.6x10^{-01}$	0.065 (0.047)	$1.7x10^{-01}$
ING5	2q37.3	20	0.084	-0.13 (0.061)	$2.8x10^{-02}$	0.028 (0.068)	$6.8x10^{-01}$	-0.062 (0.045)	$1.7x10^{-01}$
SLC38A4	12q13.11	14	0.027	0.22 (0.10)	$3.3x10^{-02}$	-0.034 (0.11)	$7.6x10^{-01}$	0.10 (0.076)	$1.7x10^{-01}$
GOLGA3	12q24.33	44	0.034	0.10 (0.049)	$3.4x10^{-02}$	-0.038 (0.067)	$5.8x10^{-01}$	0.055 (0.040)	$1.7x10^{-01}$
CCDC149	4p15.2	53	0.05	0.11 (0.052)	$4.0x10^{-02}$	-0.021 (0.063)	$7.4x10^{-01}$	0.055 (0.040)	$1.7x10^{-01}$
TEX29	13q34	31	0.091	0.11 (0.057)	$4.6x10^{-02}$	-0.013 (0.067)	$8.5x10^{-01}$	0.060 (0.043)	$1.7x10^{-01}$

Gene	Location[a]	# SNPs in Prediction	Imputation Quality[b]	Discovery U4C Meta-analysis Beta (SE)	Discovery U4C Meta-analysis *p*-value	UK Biobank Replication Beta (SE)	UK Biobank Replication *p*-value	Joint Meta-analysis Beta (SE)	Joint Meta-analysis *p*-value
ARVCF	22q11.21	3	0.022	0.68 (0.34)	$4.7x10^{-02}$	-0.65 (0.71)	$3.6x10^{-01}$	0.43 (0.31)	$1.7x10^{-01}$
HAUS6	9p22.1	12	0.042	-0.17 (0.077)	$2.5x10^{-02}$	0.051 (0.091)	$5.7x10^{-01}$	-0.079 (0.058)	$1.8x10^{-01}$
AP3B2	15q25.2	17	0.29	0.076 (0.037)	$4.0x10^{-02}$	-0.0083 (0.039)	$8.3x10^{-01}$	0.036 (0.027)	$1.8x10^{-01}$
ZNF575	19q13.31	13	0.38	-0.11 (0.038)	$5.4x10^{-03}$	0.049 (0.042)	$2.5x10^{-01}$	-0.037 (0.028)	$1.9x10^{-01}$
NLK	17q11.2	62	0.065	-0.067 (0.027)	$1.3x10^{-02}$	0.039 (0.036)	$2.7x10^{-01}$	-0.028 (0.022)	$1.9x10^{-01}$
SPATA2L	16q24.3	12	0.048	0.19 (0.077)	$1.4x10^{-02}$	-0.086 (0.094)	$3.6x10^{-01}$	0.078 (0.060)	$1.9x10^{-01}$
NUDT19	19q13.11	24	0.25	-0.079 (0.035)	$2.3x10^{-02}$	0.040 (0.047)	$3.9x10^{-01}$	-0.037 (0.028)	$1.9x10^{-01}$
RHBDL1	16p13.3	24	0.058	-0.15 (0.061)	$1.5x10^{-02}$	0.081 (0.078)	$3.0x10^{-01}$	-0.061 (0.048)	$2.0x10^{-01}$
VCPKMT	14q21.3	1	0.035	-0.26 (0.12)	$2.4x10^{-02}$	0.24 (0.19)	$2.2x10^{-01}$	-0.13 (0.099)	$2.0x10^{-01}$
ZNF592	15q25.3	12	0.034	-0.23 (0.10)	$2.8x10^{-02}$	0.065 (0.12)	$5.9x10^{-01}$	-0.1 (0.079)	$2.0x10^{-01}$
ANKRD36	2q11.2	52	0.1	0.098 (0.041)	$1.7x10^{-02}$	-0.033 (0.045)	$4.6x10^{-01}$	0.038 (0.030)	$2.1x10^{-01}$
LPGAT1	1q32.3	1	0.023	-3.6 (1.6)	$2.9x10^{-02}$	1.5 (2.1)	$4.5x10^{-01}$	-1.6 (1.3)	$2.1x10^{-01}$
RRAS	19q13.33	6	0.074	-0.17 (0.083)	$3.7x10^{-02}$	0.068 (0.10)	$5.2x10^{-01}$	-0.081 (0.065)	$2.1x10^{-01}$
ZMAT3	3q26.32	27	0.029	0.14 (0.070)	$4.2x10^{-02}$	-0.044 (0.086)	$6.1x10^{-01}$	0.068 (0.054)	$2.1x10^{-01}$
ZNF461	19q13.12	46	0.021	-0.17 (0.055)	$2.2x10^{-03}$	0.16 (0.074)	$3.5x10^{-02}$	-0.054 (0.044)	$2.2x10^{-01}$
CCK	3p22.1	24	0.02	0.17 (0.078)	$3.4x10^{-02}$	-0.069 (0.098)	$4.8x10^{-01}$	0.074 (0.061)	$2.2x10^{-01}$
STX4	16p11.2	25	0.032	-0.17 (0.069)	$1.5x10^{-02}$	0.080 (0.080)	$3.2x10^{-01}$	-0.062 (0.052)	$2.3x10^{-01}$
STARD10	11q13.4	1	0.028	0.98 (0.47)	$3.9x10^{-02}$	-0.34 (0.56)	$5.5x10^{-01}$	0.43 (0.36)	$2.3x10^{-01}$
SMPD2	6q21	15	0.083	0.17 (0.065)	$7.7x10^{-03}$	-0.13 (0.085)	$1.2x10^{-01}$	0.060 (0.051)	$2.4x10^{-01}$
ERI1	8p23.1	22	0.059	0.13 (0.064)	$3.4x10^{-02}$	-0.063 (0.079)	$4.3x10^{-01}$	0.058 (0.050)	$2.5x10^{-01}$
HRCT1	9p13.3	23	0.08	0.13 (0.063)	$3.8x10^{-02}$	-0.043 (0.070)	$5.3x10^{-01}$	0.053 (0.047)	$2.6x10^{-01}$
TTC30A	2q31.2	66	0.16	0.074 (0.032)	$1.9x10^{-02}$	-0.084 (0.050)	$9.5x10^{-02}$	0.030 (0.027)	$2.7x10^{-01}$
MT1A	16q12.2	25	0.057	-0.14 (0.061)	$2.0x10^{-02}$	0.14 (0.092)	$1.3x10^{-01}$	-0.056 (0.051)	$2.7x10^{-01}$
C1QTNF4	11p11.2	17	0.083	0.14 (0.062)	$2.6x10^{-02}$	-0.079 (0.078)	$3.1x10^{-01}$	0.054 (0.049)	$2.7x10^{-01}$
PACRGL	4p15.31	37	0.14	0.086 (0.042)	$4.0x10^{-02}$	-0.034 (0.049)	$4.8x10^{-01}$	0.035 (0.032)	$2.7x10^{-01}$
CALML6	1p36.33	11	0.2	0.10 (0.049)	$3.3x10^{-02}$	-0.056 (0.061)	$3.5x10^{-01}$	0.041 (0.038)	$2.8x10^{-01}$
GNPDA2	4p12	47	0.22	-0.057 (0.027)	$3.7x10^{-02}$	0.048 (0.040)	$2.3x10^{-01}$	-0.024 (0.022)	$2.9x10^{-01}$

Gene	Location[a]	# SNPs in Prediction	Imputation Quality[b]	Discovery U4C Meta-analysis Beta (SE)	Discovery U4C Meta-analysis *p*-value	UK Biobank Replication Beta (SE)	UK Biobank Replication *p*-value	Joint Meta-analysis Beta (SE)	Joint Meta-analysis *p*-value
FZD9	7q11.23	2	0.037	0.31 (0.15)	$3.3x10^{-02}$	-0.16 (0.18)	$3.6x10^{-01}$	0.12 (0.11)	$3.0x10^{-01}$
MTBP	8q24.12	15	0.055	-0.15 (0.076)	$4.2x10^{-02}$	0.19 (0.13)	$1.4x10^{-01}$	-0.068 (0.066)	$3.0x10^{-01}$
B3GAT2	6q13	64	0.14	0.083 (0.033)	$1.2x10^{-02}$	-0.063 (0.041)	$1.3x10^{-01}$	0.026 (0.026)	$3.1x10^{-01}$
MCM3AP	21q22.3	5	0.09	-0.18 (0.074)	$1.7x10^{-02}$	0.11 (0.088)	$2.0x10^{-01}$	-0.058 (0.057)	$3.1x10^{-01}$
TTLL13	15q26.1	24	0.029	0.14 (0.066)	$3.6x10^{-02}$	-0.11 (0.094)	$2.3x10^{-01}$	0.056 (0.054)	$3.1x10^{-01}$
SULF2	20q13.13	9	0.037	-0.2 (0.098)	$4.0x10^{-02}$	0.13 (0.13)	$3.1x10^{-01}$	-0.077 (0.077)	$3.1x10^{-01}$
TNNI3K	1p31.1	28	0.11	-0.097 (0.044)	$2.9x10^{-02}$	0.11 (0.069)	$1.1x10^{-01}$	-0.037 (0.037)	$3.2x10^{-01}$
MYOZ3	5q33.1	53	0.019	-0.13 (0.062)	$3.5x10^{-02}$	0.068 (0.073)	$3.5x10^{-01}$	-0.048 (0.048)	$3.2x10^{-01}$
EDC4	16q22.1	18	0.052	-0.15 (0.071)	$4.0x10^{-02}$	0.082 (0.087)	$3.5x10^{-01}$	-0.055 (0.055)	$3.2x10^{-01}$
ZNF660	3p21.31	54	0.34	0.056 (0.026)	$3.3x10^{-02}$	-0.031 (0.031)	$3.2x10^{-01}$	0.020 (0.020)	$3.3x10^{-01}$
MSR1	8p22	9	0.024	0.32 (0.12)	$8.7x10^{-03}$	-0.2 (0.13)	$1.5x10^{-01}$	0.086 (0.091)	$3.4x10^{-01}$
MYLK2	20q11.21	8	0.031	0.31 (0.14)	$2.8x10^{-02}$	-0.061 (0.11)	$5.8x10^{-01}$	0.081 (0.086)	$3.5x10^{-01}$
TMEM206	1q32.3	22	0.049	0.13 (0.054)	$1.6x10^{-02}$	-0.26 (0.10)	$9.7x10^{-03}$	0.044 (0.048)	$3.6x10^{-01}$
NARF	17q25.3	12	0.17	-0.13 (0.053)	$1.7x10^{-02}$	0.093 (0.064)	$1.5x10^{-01}$	-0.037 (0.041)	$3.6x10^{-01}$
ZNF502	3p21.31	13	0.21	-0.14 (0.060)	$1.9x10^{-02}$	0.060 (0.059)	$3.1x10^{-01}$	-0.038 (0.042)	$3.6x10^{-01}$
DOCK8	9p24.3	22	0.11	-0.11 (0.057)	$4.9x10^{-02}$	0.024 (0.049)	$6.3x10^{-01}$	-0.034 (0.037)	$3.6x10^{-01}$
PXK	3p14.3	8	0.069	0.22 (0.076)	$4.5x10^{-03}$	-0.21 (0.097)	$3.1x10^{-02}$	0.054 (0.060)	$3.7x10^{-01}$
EPHB1	3q22.2	37	0.055	-0.16 (0.065)	$1.5x10^{-02}$	0.082 (0.067)	$2.2x10^{-01}$	-0.042 (0.047)	$3.7x10^{-01}$
ZNF131	5p12	19	0.047	-0.14 (0.067)	$4.3x10^{-02}$	0.073 (0.076)	$3.3x10^{-01}$	-0.044 (0.050)	$3.8x10^{-01}$
PRIMPOL	4q35.1	51	0.13	-0.083 (0.040)	$4.1x10^{-02}$	0.053 (0.048)	$2.7x10^{-01}$	-0.027 (0.031)	$3.9x10^{-01}$
AGAP9	10q11.22	10	0.065	-0.15 (0.070)	$3.6x10^{-02}$	0.13 (0.092)	$1.6x10^{-01}$	-0.046 (0.056)	$4.1x10^{-01}$
BCAT1	12p12.1	14	0.027	-0.17 (0.083)	$3.7x10^{-02}$	0.17 (0.11)	$1.4x10^{-01}$	-0.056 (0.067)	$4.1x10^{-01}$
SVIP	11p14.3	17	0.16	0.15 (0.066)	$2.7x10^{-02}$	-0.064 (0.063)	$3.1x10^{-01}$	0.035 (0.046)	$4.4x10^{-01}$
MYH1	17p13.1	54	0.032	-0.12 (0.052)	$2.8x10^{-02}$	0.11 (0.066)	$1.1x10^{-01}$	-0.03 (0.041)	$4.7x10^{-01}$
TOX2	20q13.12	32	0.33	0.068 (0.033)	$3.9x10^{-02}$	-0.058 (0.041)	$1.6x10^{-01}$	0.018 (0.026)	$4.7x10^{-01}$
RP11-295P9.3	10p13	17	0.15	0.11 (0.055)	$4.5x10^{-02}$	-0.092 (0.068)	$1.7x10^{-01}$	0.030 (0.042)	$4.8x10^{-01}$
SGCG	13q12.12	1	0.049	-2.9 (1.2)	$1.7x10^{-02}$	1.3 (1.1)	$2.2x10^{-01}$	-0.55 (0.80)	$4.9x10^{-01}$

Gene	Location[a]	# SNPs in Prediction	Imputation Quality[b]	Discovery U4C Meta-analysis Beta (SE)	Discovery U4C Meta-analysis *p*-value	UK Biobank Replication Beta (SE)	UK Biobank Replication *p*-value	Joint Meta-analysis Beta (SE)	Joint Meta-analysis *p*-value
TRMT61B	2p23.2	20	0.2	-0.088 (0.044)	4.4×10^{-02}	0.069 (0.052)	1.8×10^{-01}	-0.022 (0.033)	5.0×10^{-01}
PHYHIPL	10q21.1	31	0.022	0.16 (0.060)	8.4×10^{-03}	-0.22 (0.085)	9.0×10^{-03}	0.032 (0.049)	5.1×10^{-01}
TMEM98	17q11.2	14	0.047	-0.23 (0.089)	1.0×10^{-02}	0.28 (0.11)	1.4×10^{-02}	-0.036 (0.070)	6.0×10^{-01}
ZNF501	3p21.31	2	0.12	-0.21 (0.095)	2.6×10^{-02}	0.14 (0.093)	1.5×10^{-01}	-0.035 (0.067)	6.0×10^{-01}
COLEC11	2p25.3	33	0.026	0.17 (0.068)	1.5×10^{-02}	-0.22 (0.092)	1.5×10^{-02}	0.027 (0.055)	6.2×10^{-01}
HAGH	16p13.3	14	0.098	0.13 (0.060)	3.3×10^{-02}	-0.14 (0.074)	5.9×10^{-02}	0.022 (0.047)	6.4×10^{-01}
HELLS	10q23.33	32	0.049	0.18 (0.079)	1.9×10^{-02}	-0.23 (0.10)	2.4×10^{-02}	0.028 (0.062)	6.5×10^{-01}
KAT8	16p11.2	38	0.24	-0.068 (0.033)	3.8×10^{-02}	0.047 (0.033)	1.5×10^{-01}	-0.011 (0.023)	6.5×10^{-01}
LRRFIP1	2q37.3	15	0.024	-0.23 (0.11)	3.3×10^{-02}	0.31 (0.14)	3.4×10^{-02}	-0.038 (0.086)	6.6×10^{-01}
ZNF709	19p13.2	20	0.027	0.21 (0.096)	2.9×10^{-02}	-0.18 (0.10)	7.7×10^{-02}	0.029 (0.071)	6.8×10^{-01}
RGS17	6q25.2	1	0.038	2.2 (1.1)	4.6×10^{-02}	-1.2 (0.97)	2.3×10^{-01}	0.30 (0.73)	6.8×10^{-01}
MSH4	1p31.1	27	0.12	0.10 (0.050)	4.4×10^{-02}	-0.085 (0.052)	1.1×10^{-01}	0.013 (0.036)	7.2×10^{-01}
COMMD10	5q23.1	57	0.1	0.085 (0.042)	4.3×10^{-02}	-0.13 (0.055)	1.6×10^{-02}	0.0053 (0.033)	8.7×10^{-01}

(B) Whole Blood Expression

Gene	Location[a]	# SNPs in Prediction	Imputation Quality[b]	Discovery U4C Meta-analysis Beta (SE)	Discovery U4C Meta-analysis *p*-value	UK Biobank Replication Beta (SE)	UK Biobank Replication *p*-value	Joint Meta-analysis Beta (SE)	Joint Meta-analysis *p*-value
RCCD1	**15q26.1**	**20**	**0.35**	**-0.074 (0.026)**	**4.7×10^{-03}**	**-0.14 (0.039)**	**2.7×10^{-04}**	**-0.095 (0.022)**	**1.2×10^{-05}**
ACAP1	**17p13.1**	**19**	**0.39**	**0.098 (0.037)**	**7.9×10^{-03}**	**0.11 (0.033)**	**7.9×10^{-04}**	**0.11 (0.025)**	**1.9×10^{-05}**
LRRC25	**19p13.11**	**33**	**0.35**	**0.086 (0.029)**	**2.7×10^{-03}**	**0.094 (0.034)**	**6.5×10^{-03}**	**0.089 (0.022)**	**5.2×10^{-05}**
TMEM150C	4q21.22	18	0.12	0.16 (0.058)	4.7×10^{-03}	0.17 (0.065)	9.9×10^{-03}	0.17 (0.043)	1.3×10^{-04}
TNNT3	11p15.5	11	0.38	0.080 (0.028)	3.9×10^{-03}	0.084 (0.035)	1.7×10^{-02}	0.082 (0.022)	1.8×10^{-04}
HP	16q22.2	20	0.49	0.11 (0.024)	7.2×10^{-06}	0.011 (0.029)	7.0×10^{-01}	0.069 (0.019)	2.1×10^{-04}
CTDNEP1	17p13.1	22	0.14	0.15 (0.050)	3.0×10^{-03}	0.13 (0.058)	3.1×10^{-02}	0.14 (0.038)	2.5×10^{-04}
DHODH	16q22.2	59	0.1	-0.14 (0.041)	4.2×10^{-04}	-0.074 (0.053)	1.6×10^{-01}	-0.12 (0.032)	2.6×10^{-04}
DPY19L1	7p14.2	24	0.3	0.11 (0.029)	7.9×10^{-05}	0.030 (0.038)	4.3×10^{-01}	0.084 (0.023)	2.8×10^{-04}
KIN	10p14	23	0.11	0.12 (0.054)	2.6×10^{-02}	0.19 (0.066)	4.6×10^{-03}	0.15 (0.042)	4.3×10^{-04}
APOBEC3A	22q13.1	78	0.16	-0.10 (0.036)	5.7×10^{-03}	-0.094 (0.045)	3.9×10^{-02}	-0.098 (0.028)	5.6×10^{-04}
KCTD11	17p13.1	37	0.25	-0.093 (0.047)	4.8×10^{-02}	-0.12 (0.041)	4.7×10^{-03}	-0.11 (0.031)	6.0×10^{-04}
VEZT	12q22	4	0.0077	-0.41 (0.18)	2.1×10^{-02}	-0.97 (0.33)	3.8×10^{-03}	-0.54 (0.16)	6.7×10^{-04}
CCNE1	19q12	6	0.015	-0.59 (0.29)	4.1×10^{-02}	-0.65 (0.24)	7.6×10^{-03}	-0.63 (0.19)	7.9×10^{-04}
SCN4B	11q23.3	33	0.05	-0.15 (0.078)	5.0×10^{-02}	-0.27 (0.095)	4.5×10^{-03}	-0.20 (0.060)	9.0×10^{-04}
ALDH5A1	6p22.3	45	0.11	-0.098 (0.046)	3.3×10^{-02}	-0.17 (0.063)	7.4×10^{-03}	-0.12 (0.037)	9.7×10^{-04}
GIT1	17q11.2	31	0.4	-0.053 (0.026)	4.0×10^{-02}	-0.086 (0.032)	7.0×10^{-03}	-0.066 (0.020)	9.9×10^{-04}
KCNAB3	17p13.1	34	0.093	-0.17 (0.063)	5.5×10^{-03}	-0.13 (0.073)	7.8×10^{-02}	-0.16 (0.048)	1.1×10^{-03}
ELL	19p13.11	37	0.34	-0.071 (0.029)	1.6×10^{-02}	-0.076 (0.035)	2.8×10^{-02}	-0.073 (0.022)	1.1×10^{-03}
ZNF23	16q22.2	25	0.15	0.11 (0.050)	2.1×10^{-02}	0.13 (0.054)	2.0×10^{-02}	0.12 (0.037)	1.1×10^{-03}
ACADVL	17p13.1	23	0.12	-0.14 (0.055)	1.3×10^{-02}	-0.13 (0.065)	4.0×10^{-02}	-0.14 (0.042)	1.2×10^{-03}
ITGB7	12q13.13	25	0.17	-0.080 (0.026)	2.3×10^{-03}	-0.057 (0.052)	2.8×10^{-01}	-0.076 (0.024)	1.3×10^{-03}
DPYD	1p21.3	108	0.13	-0.073 (0.037)	4.8×10^{-02}	-0.14 (0.051)	6.0×10^{-03}	-0.096 (0.030)	1.3×10^{-03}
AP1B1	22q12.2	12	0.04	-0.21 (0.082)	9.9×10^{-03}	-0.21 (0.11)	6.2×10^{-02}	-0.21 (0.066)	1.4×10^{-03}

Gene	Location[a]	# SNPs in Prediction	Imputation Quality[b]	Discovery U4C Meta-analysis Beta (SE)	Discovery U4C Meta-analysis *p*-value	UK Biobank Replication Beta (SE)	UK Biobank Replication *p*-value	Joint Meta-analysis Beta (SE)	Joint Meta-analysis *p*-value
SASS6	1p21.2	24	0.019	-0.24 (0.11)	$2.3x10^{-02}$	-0.32 (0.14)	$2.6x10^{-02}$	-0.27 (0.086)	$1.6x10^{-03}$
TTLL5	14q24.3	27	0.16	0.13 (0.048)	$6.4x10^{-03}$	0.092 (0.058)	$1.1x10^{-01}$	0.11 (0.037)	$1.8x10^{-03}$
ZNF564	19p13.2	24	0.011	-0.27 (0.10)	$8.5x10^{-03}$	-0.26 (0.15)	$9.4x10^{-02}$	-0.27 (0.085)	$1.8x10^{-03}$
GPM6A	4q34.2	20	0.39	-0.043 (0.021)	$4.4x10^{-02}$	-0.087 (0.033)	$8.6x10^{-03}$	-0.056 (0.018)	$1.8x10^{-03}$
KLHL28	14q21.2	55	0.022	0.16 (0.069)	$1.7x10^{-02}$	0.21 (0.10)	$4.3x10^{-02}$	0.18 (0.057)	$1.9x10^{-03}$
POLN	4p16.3	41	0.079	-0.097 (0.033)	$2.9x10^{-03}$	-0.066 (0.075)	$3.7x10^{-01}$	-0.092 (0.030)	$2.0x10^{-03}$
CCT5	5p15.2	21	0.042	-0.22 (0.085)	$8.4x10^{-03}$	-0.22 (0.14)	$1.1x10^{-01}$	-0.22 (0.072)	$2.0x10^{-03}$
CC2D1A	19p13.12	19	0.037	-0.27 (0.098)	$6.1x10^{-03}$	-0.17 (0.11)	$1.3x10^{-01}$	-0.23 (0.074)	$2.1x10^{-03}$
SSBP4	19p13.11	29	0.067	0.15 (0.061)	$1.6x10^{-02}$	0.15 (0.079)	$5.4x10^{-02}$	0.15 (0.048)	$2.1x10^{-03}$
KBTBD3	11q22.3	32	0.051	0.15 (0.075)	$4.5x10^{-02}$	0.24 (0.097)	$1.4x10^{-02}$	0.18 (0.059)	$2.1x10^{-03}$
MAN2C1	15q24.2	45	0.77	-0.051 (0.017)	$2.1x10^{-03}$	-0.022 (0.022)	$3.2x10^{-01}$	-0.040 (0.013)	$2.2x10^{-03}$
FAM103A1	15q25.2	29	0.013	-0.23 (0.085)	$5.6x10^{-03}$	-0.15 (0.11)	$1.6x10^{-01}$	-0.20 (0.067)	$2.3x10^{-03}$
SLC35E2B	1p36.33	18	0.081	-0.20 (0.085)	$1.8x10^{-02}$	-0.17 (0.085)	$5.2x10^{-02}$	-0.18 (0.060)	$2.4x10^{-03}$
TP53INP2	20q11.22	6	0.02	-0.50 (0.13)	$1.0x10^{-04}$	0.004 (0.16)	$9.8x10^{-01}$	-0.30 (0.10)	$2.5x10^{-03}$
ZNF578	19q13.41	9	0.028	0.26 (0.11)	$1.4x10^{-02}$	0.30 (0.17)	$8.0x10^{-02}$	0.27 (0.090)	$2.6x10^{-03}$
THUMPD1	16p12.3	57	0.23	0.12 (0.043)	$3.5x10^{-03}$	0.059 (0.048)	$2.2x10^{-01}$	0.095 (0.032)	$2.7x10^{-03}$
STX16	20q13.32	22	0.066	-0.18 (0.069)	$9.2x10^{-03}$	-0.13 (0.084)	$1.4x10^{-01}$	-0.16 (0.054)	$3.1x10^{-03}$
CA12	15q22.2	42	0.026	-0.21 (0.086)	$1.2x10^{-02}$	-0.19 (0.12)	$1.1x10^{-01}$	-0.20 (0.069)	$3.1x10^{-03}$
UBE4A	11q23.3	29	0.057	-0.16 (0.075)	$3.9x10^{-02}$	-0.19 (0.093)	$3.6x10^{-02}$	-0.17 (0.058)	$3.4x10^{-03}$
PLEKHJ1	19p13.3	10	0.044	0.20 (0.064)	$1.4x10^{-03}$	0.016 (0.12)	$8.9x10^{-01}$	0.17 (0.057)	$3.6x10^{-03}$
TMIGD2	19p13.3	18	0.085	-0.17 (0.060)	$5.2x10^{-03}$	-0.088 (0.080)	$2.7x10^{-01}$	-0.14 (0.048)	$3.7x10^{-03}$
ABHD8	19p13.11	11	0.11	0.16 (0.054)	$4.2x10^{-03}$	0.071 (0.070)	$3.1x10^{-01}$	0.12 (0.043)	$3.9x10^{-03}$
ITGA2	5q11.2	49	0.12	0.10 (0.043)	$1.6x10^{-02}$	0.093 (0.059)	$1.2x10^{-01}$	0.10 (0.035)	$4.0x10^{-03}$
DONSON	21q22.11	23	0.014	0.40 (0.14)	$3.1x10^{-03}$	0.15 (0.16)	$3.5x10^{-01}$	0.30 (0.10)	$4.1x10^{-03}$
SLFN11	17q12	35	0.079	0.093 (0.045)	$3.8x10^{-02}$	0.15 (0.073)	$3.4x10^{-02}$	0.11 (0.038)	$4.1x10^{-03}$
XRCC2	7q36.1	18	0.15	0.13 (0.051)	$9.5x10^{-03}$	0.078 (0.054)	$1.5x10^{-01}$	0.11 (0.037)	$4.2x10^{-03}$
HRSP12	8q22.2	38	0.09	-0.16 (0.058)	$6.5x10^{-03}$	-0.082 (0.071)	$2.5x10^{-01}$	-0.13 (0.045)	$4.5x10^{-03}$

Gene	Location[a]	# SNPs in Prediction	Imputation Quality[b]	Discovery U4C Meta-analysis Beta (SE)	Discovery U4C Meta-analysis *p*-value	UK Biobank Replication Beta (SE)	UK Biobank Replication *p*-value	Joint Meta-analysis Beta (SE)	Joint Meta-analysis *p*-value
PRKG2	4q21.21	32	0.15	-0.097 (0.040)	$1.7x10^{-02}$	-0.088 (0.057)	$1.2x10^{-01}$	-0.094 (0.033)	$4.5x10^{-03}$
EEA1	12q22	22	0.008	-0.38 (0.17)	$2.5x10^{-02}$	-0.36 (0.21)	$8.2x10^{-02}$	-0.37 (0.13)	$4.5x10^{-03}$
NYNRIN	14q12	76	0.052	-0.12 (0.049)	$1.6x10^{-02}$	-0.10 (0.066)	$1.4x10^{-01}$	-0.11 (0.039)	$4.7x10^{-03}$
RPS10	6p21.31	29	0.035	0.17 (0.059)	$4.3x10^{-03}$	0.070 (0.11)	$5.4x10^{-01}$	0.15 (0.053)	$4.8x10^{-03}$
TMEM143	19q13.33	9	0.059	0.21 (0.092)	$2.1x10^{-02}$	0.17 (0.100)	$9.7x10^{-02}$	0.19 (0.068)	$4.8x10^{-03}$
GLI3	7p14.1	16	0.043	0.22 (0.100)	$2.7x10^{-02}$	0.21 (0.12)	$7.8x10^{-02}$	0.22 (0.077)	$4.8x10^{-03}$
ZNF879	5q35.3	16	0.18	-0.086 (0.035)	$1.4x10^{-02}$	-0.072 (0.052)	$1.6x10^{-01}$	-0.081 (0.029)	$4.9x10^{-03}$
UGGT2	13q32.1	47	0.15	-0.091 (0.043)	$3.2x10^{-02}$	-0.096 (0.053)	$6.8x10^{-02}$	-0.093 (0.033)	$4.9x10^{-03}$
MYCL1	1p34.2	25	0.014	-0.25 (0.12)	$3.9x10^{-02}$	-0.26 (0.14)	$5.8x10^{-02}$	-0.26 (0.092)	$5.1x10^{-03}$
ELF3	1q32.1	53	0.28	-0.093 (0.029)	$1.3x10^{-03}$	-0.015 (0.037)	$6.9x10^{-01}$	-0.064 (0.023)	$5.4x10^{-03}$
CCDC117	22q12.1	24	0.02	0.29 (0.10)	$4.1x10^{-03}$	0.11 (0.15)	$4.6x10^{-01}$	0.23 (0.084)	$5.4x10^{-03}$
CRCP	7q11.21	47	0.12	-0.082 (0.034)	$1.6x10^{-02}$	-0.070 (0.049)	$1.5x10^{-01}$	-0.078 (0.028)	$5.4x10^{-03}$
KCNIP2	10q24.32	13	0.055	-0.24 (0.083)	$3.5x10^{-03}$	-0.080 (0.12)	$5.0x10^{-01}$	-0.19 (0.068)	$5.5x10^{-03}$
RDBP	6p21.33	17	0.076	-0.16 (0.059)	$7.4x10^{-03}$	-0.085 (0.083)	$3.1x10^{-01}$	-0.13 (0.048)	$5.5x10^{-03}$
ZSCAN20	1p35.1	46	0.015	0.17 (0.084)	$4.2x10^{-02}$	0.22 (0.11)	$5.4x10^{-02}$	0.19 (0.068)	$5.5x10^{-03}$
TRAF3IP3	1q32.2	41	0.21	-0.11 (0.036)	$2.0x10^{-03}$	-0.026 (0.045)	$5.6x10^{-01}$	-0.078 (0.028)	$5.6x10^{-03}$
STAP2	19p13.3	14	0.054	0.21 (0.083)	$1.1x10^{-02}$	0.13 (0.11)	$2.1x10^{-01}$	0.18 (0.065)	$5.6x10^{-03}$
INCENP	11q12.3	45	0.084	-0.13 (0.062)	$4.0x10^{-02}$	-0.13 (0.070)	$6.4x10^{-02}$	-0.13 (0.047)	$5.6x10^{-03}$
GLB1	3p22.3	23	0.041	-0.22 (0.091)	$1.8x10^{-02}$	-0.15 (0.10)	$1.3x10^{-01}$	-0.19 (0.068)	$5.7x10^{-03}$
CASP8	2q33.1	30	0.33	-0.060 (0.026)	$2.1x10^{-02}$	-0.056 (0.037)	$1.3x10^{-01}$	-0.059 (0.021)	$5.7x10^{-03}$
OTUD7B	1q21.2	9	0.017	0.43 (0.14)	$1.8x10^{-03}$	0.088 (0.17)	$6.1x10^{-01}$	0.30 (0.11)	$5.8x10^{-03}$
SMAD3	15q22.33	43	0.008	-0.29 (0.097)	$3.2x10^{-03}$	-0.086 (0.13)	$5.1x10^{-01}$	-0.21 (0.078)	$5.8x10^{-03}$
AATF	17q12	27	0.028	-0.29 (0.11)	$9.1x10^{-03}$	-0.16 (0.14)	$2.4x10^{-01}$	-0.24 (0.087)	$5.8x10^{-03}$
ZNF367	9q22.32	60	0.026	-0.15 (0.070)	$3.3x10^{-02}$	-0.16 (0.089)	$7.8x10^{-02}$	-0.15 (0.055)	$5.8x10^{-03}$
WNT10B	12q13.12	1	0.0075	0.54 (0.26)	$3.6x10^{-02}$	0.57 (0.32)	$7.4x10^{-02}$	0.56 (0.20)	$5.9x10^{-03}$
LRRFIP2	3p22.2	63	0.2	0.093 (0.042)	$2.7x10^{-02}$	0.075 (0.045)	$9.8x10^{-02}$	0.084 (0.031)	$6.0x10^{-03}$
C22orf32	22q13.2	29	0.86	-0.039 (0.018)	$2.6x10^{-02}$	-0.034 (0.021)	$1.1x10^{-01}$	-0.037 (0.014)	$6.5x10^{-03}$

Gene	Location[a]	# SNPs in Prediction	Imputation Quality[b]	Discovery U4C Meta-analysis Beta (SE)	Discovery U4C Meta-analysis *p*-value	UK Biobank Replication Beta (SE)	UK Biobank Replication *p*-value	Joint Meta-analysis Beta (SE)	Joint Meta-analysis *p*-value
ZNF16	8q24.3	21	0.014	0.21 (0.10)	$3.6x10^{-02}$	0.25 (0.14)	$8.2x10^{-02}$	0.22 (0.083)	$6.5x10^{-03}$
APH1A	1q21.2	2	0.0058	2.80 (1.0)	$6.8x10^{-03}$	1.20 (1.2)	$3.2x10^{-01}$	2.20 (0.80)	$6.8x10^{-03}$
STX1A	7q11.23	18	0.031	-0.23 (0.10)	$2.8x10^{-02}$	-0.19 (0.12)	$1.1x10^{-01}$	-0.21 (0.079)	$6.8x10^{-03}$
C12orf32	12p13.33	15	0.3	-0.061 (0.030)	$4.2x10^{-02}$	-0.078 (0.043)	$7.0x10^{-02}$	-0.067 (0.025)	$6.8x10^{-03}$
TTL	2q13	25	0.033	0.20 (0.082)	$1.8x10^{-02}$	0.15 (0.11)	$1.8x10^{-01}$	0.18 (0.067)	$6.9x10^{-03}$
XCL2	1q24.2	23	0.51	-0.053 (0.026)	$4.4x10^{-02}$	-0.051 (0.029)	$7.3x10^{-02}$	-0.052 (0.019)	$6.9x10^{-03}$
PF4V1	4q13.3	37	0.58	0.040 (0.020)	$4.8x10^{-02}$	0.046 (0.025)	$6.7x10^{-02}$	0.042 (0.016)	$7.1x10^{-03}$
IRF2BP2	1q42.3	77	0.048	-0.13 (0.055)	$1.5x10^{-02}$	-0.087 (0.069)	$2.1x10^{-01}$	-0.12 (0.043)	$7.2x10^{-03}$
LGMN	14q32.12	35	0.061	-0.15 (0.070)	$3.9x10^{-02}$	-0.15 (0.088)	$8.9x10^{-02}$	-0.15 (0.055)	$7.4x10^{-03}$
RIOK2	5q15	8	0.059	-0.20 (0.075)	$6.7x10^{-03}$	-0.084 (0.098)	$3.9x10^{-01}$	-0.16 (0.060)	$7.5x10^{-03}$
FAM200B	4p15.32	15	0.008	-0.40 (0.16)	$1.4x10^{-02}$	-0.27 (0.23)	$2.5x10^{-01}$	-0.35 (0.13)	$7.5x10^{-03}$
TUBGCP4	15q15.3	21	0.092	0.12 (0.057)	$3.7x10^{-02}$	0.11 (0.068)	$9.4x10^{-02}$	0.12 (0.043)	$7.6x10^{-03}$
SOD2	6q25.3	39	0.32	0.11 (0.040)	$7.8x10^{-03}$	0.043 (0.036)	$2.3x10^{-01}$	0.071 (0.027)	$7.7x10^{-03}$
IRAK3	12q14.3	47	0.13	0.089 (0.037)	$1.6x10^{-02}$	0.069 (0.059)	$2.4x10^{-01}$	0.083 (0.031)	$7.7x10^{-03}$
SDC2	8q22.1	46	0.054	0.19 (0.078)	$1.7x10^{-02}$	0.12 (0.092)	$2.0x10^{-01}$	0.16 (0.060)	$7.7x10^{-03}$
SYNE2	14q23.2	45	0.036	-0.14 (0.066)	$3.3x10^{-02}$	-0.16 (0.099)	$1.1x10^{-01}$	-0.15 (0.055)	$7.8x10^{-03}$
FAIM	3q22.3	12	0.061	0.28 (0.086)	$1.1x10^{-03}$	0.037 (0.093)	$6.9x10^{-01}$	0.17 (0.063)	$7.9x10^{-03}$
RINT1	7q22.3	16	0.045	0.20 (0.085)	$1.8x10^{-02}$	0.14 (0.12)	$2.1x10^{-01}$	0.18 (0.068)	$8.1x10^{-03}$
S100Z	5q13.3	32	0.16	0.10 (0.045)	$2.3x10^{-02}$	0.081 (0.059)	$1.6x10^{-01}$	0.095 (0.036)	$8.2x10^{-03}$
YJEFN3	19p13.11	20	0.12	-0.12 (0.048)	$1.5x10^{-02}$	-0.071 (0.061)	$2.4x10^{-01}$	-0.10 (0.038)	$8.3x10^{-03}$
CASP10	2q33.1	17	0.059	-0.24 (0.087)	$6.7x10^{-03}$	-0.085 (0.10)	$4.0x10^{-01}$	-0.17 (0.066)	$9.2x10^{-03}$
C19orf57	19p13.12	27	0.0062	-0.51 (0.22)	$1.9x10^{-02}$	-0.34 (0.28)	$2.3x10^{-01}$	-0.45 (0.17)	$9.6x10^{-03}$
ING5	2q37.3	17	0.0067	0.32 (0.14)	$2.2x10^{-02}$	0.23 (0.19)	$2.1x10^{-01}$	0.29 (0.11)	$9.6x10^{-03}$
ASL	7q11.21	19	0.28	0.063 (0.030)	$3.2x10^{-02}$	0.056 (0.038)	$1.4x10^{-01}$	0.061 (0.023)	$9.6x10^{-03}$
NEIL2	8p23.1	40	0.44	-0.11 (0.033)	$1.1x10^{-03}$	-0.016 (0.030)	$6.0x10^{-01}$	-0.057 (0.022)	$9.7x10^{-03}$
TMEM69	1p34.1	20	0.015	-0.27 (0.12)	$3.0x10^{-02}$	-0.21 (0.14)	$1.5x10^{-01}$	-0.24 (0.094)	$9.9x10^{-03}$

Gene	Location[a]	# SNPs in Prediction	Imputation Quality[b]	Discovery U4C Meta-analysis Beta (SE)	Discovery U4C Meta-analysis *p*-value	UK Biobank Replication Beta (SE)	UK Biobank Replication *p*-value	Joint Meta-analysis Beta (SE)	Joint Meta-analysis *p*-value
ARHGAP11A	15q13.3	95	0.26	0.12 (0.031)	$1.8x10^{-04}$	-0.019 (0.038)	$6.1x10^{-01}$	0.062 (0.024)	$1.0x10^{-02}$
SNUPN	15q24.2	24	0.37	-0.078 (0.029)	$7.1x10^{-03}$	-0.027 (0.033)	$4.0x10^{-01}$	-0.056 (0.022)	$1.0x10^{-02}$
ZNF181	19q13.11	48	0.11	-0.12 (0.047)	$1.1x10^{-02}$	-0.054 (0.061)	$3.8x10^{-01}$	-0.095 (0.037)	$1.0x10^{-02}$
STON2	14q31.1	13	0.0078	0.42 (0.18)	$2.2x10^{-02}$	0.32 (0.27)	$2.4x10^{-01}$	0.39 (0.15)	$1.0x10^{-02}$
SKIL	3q26.2	45	0.038	0.14 (0.064)	$2.9x10^{-02}$	0.12 (0.087)	$1.7x10^{-01}$	0.13 (0.052)	$1.0x10^{-02}$
TSTA3	8q24.3	2	0.017	-0.31 (0.15)	$4.2x10^{-02}$	-0.41 (0.26)	$1.1x10^{-01}$	-0.34 (0.13)	$1.0x10^{-02}$
WBSCR27	7q11.23	43	0.78	0.044 (0.015)	$3.3x10^{-03}$	0.005 (0.021)	$8.1x10^{-01}$	0.031 (0.012)	$1.1x10^{-02}$
AP3D1	19p13.3	34	0.083	-0.14 (0.055)	$1.1x10^{-02}$	-0.062 (0.078)	$4.3x10^{-01}$	-0.11 (0.045)	$1.1x10^{-02}$
CDCA4	14q32.33	4	0.15	-0.11 (0.043)	$1.1x10^{-02}$	-0.050 (0.061)	$4.1x10^{-01}$	-0.089 (0.035)	$1.1x10^{-02}$
ZNF19	16q22.2	23	0.063	-0.18 (0.073)	$1.6x10^{-02}$	-0.097 (0.086)	$2.6x10^{-01}$	-0.14 (0.056)	$1.1x10^{-02}$
SEMA4G	10q24.31	18	0.087	-0.12 (0.050)	$2.1x10^{-02}$	-0.089 (0.081)	$2.7x10^{-01}$	-0.11 (0.042)	$1.1x10^{-02}$
SERF2	15q15.3	16	0.083	0.18 (0.081)	$2.6x10^{-02}$	0.11 (0.080)	$1.7x10^{-01}$	0.15 (0.057)	$1.1x10^{-02}$
RNF187	1q42.13	56	0.14	-0.094 (0.044)	$3.1x10^{-02}$	-0.072 (0.051)	$1.6x10^{-01}$	-0.084 (0.033)	$1.1x10^{-02}$
TUB	11p15.4	77	0.052	-0.13 (0.059)	$3.4x10^{-02}$	-0.10 (0.073)	$1.6x10^{-01}$	-0.12 (0.046)	$1.1x10^{-02}$
PHLPP1	18q21.33	34	0.15	0.087 (0.042)	$3.6x10^{-02}$	0.083 (0.056)	$1.4x10^{-01}$	0.086 (0.034)	$1.1x10^{-02}$
HOOK1	1p32.1	40	0.0087	0.22 (0.11)	$4.7x10^{-02}$	0.22 (0.14)	$1.1x10^{-01}$	0.22 (0.086)	$1.1x10^{-02}$
MYOF	10q23.33	54	0.092	0.14 (0.051)	$5.3x10^{-03}$	0.034 (0.061)	$5.8x10^{-01}$	0.097 (0.039)	$1.2x10^{-02}$
PRKAB2	1q21.1	36	0.1	0.16 (0.063)	$1.0x10^{-02}$	0.062 (0.065)	$3.4x10^{-01}$	0.11 (0.045)	$1.2x10^{-02}$
GPX3	5q33.1	45	0.14	0.11 (0.046)	$1.4x10^{-02}$	0.054 (0.056)	$3.3x10^{-01}$	0.090 (0.036)	$1.2x10^{-02}$
C1orf21	1q25.3	61	0.026	0.18 (0.073)	$1.5x10^{-02}$	0.093 (0.098)	$3.4x10^{-01}$	0.15 (0.059)	$1.2x10^{-02}$
RABGAP1	9q33.2	14	0.0058	0.38 (0.16)	$1.9x10^{-02}$	0.23 (0.22)	$3.0x10^{-01}$	0.33 (0.13)	$1.2x10^{-02}$
PDIA3	15q15.3	10	0.03	0.30 (0.13)	$2.2x10^{-02}$	0.17 (0.13)	$2.0x10^{-01}$	0.24 (0.093)	$1.2x10^{-02}$
MDFIC	7q31.1	19	0.0079	-0.35 (0.16)	$3.1x10^{-02}$	-0.24 (0.18)	$1.8x10^{-01}$	-0.30 (0.12)	$1.2x10^{-02}$
ZNF83	19q13.41	42	0.61	0.043 (0.020)	$3.3x10^{-02}$	0.035 (0.025)	$1.7x10^{-01}$	0.040 (0.016)	$1.2x10^{-02}$
IL24	1q32.1	16	0.16	0.14 (0.049)	$4.8x10^{-03}$	0.031 (0.054)	$5.6x10^{-01}$	0.089 (0.036)	$1.3x10^{-02}$
RNPEP	1q32.1	57	0.77	-0.046 (0.018)	$9.2x10^{-03}$	-0.016 (0.022)	$4.8x10^{-01}$	-0.034 (0.014)	$1.3x10^{-02}$

Gene	Location[a]	# SNPs in Prediction	Imputation Quality[b]	Discovery U4C Meta-analysis Beta (SE)	Discovery U4C Meta-analysis *p*-value	UK Biobank Replication Beta (SE)	UK Biobank Replication *p*-value	Joint Meta-analysis Beta (SE)	Joint Meta-analysis *p*-value
KCTD20	6p21.31	25	0.042	0.20 (0.077)	$1.1x10^{-02}$	0.076 (0.11)	$4.9x10^{-01}$	0.16 (0.063)	$1.3x10^{-02}$
CERKL	2q31.3	59	0.32	0.059 (0.024)	$1.5x10^{-02}$	0.030 (0.035)	$3.9x10^{-01}$	0.049 (0.020)	$1.3x10^{-02}$
PIGN	18q21.33	65	0.35	0.056 (0.026)	$2.9x10^{-02}$	0.044 (0.036)	$2.3x10^{-01}$	0.052 (0.021)	$1.3x10^{-02}$
ELL3	15q15.3	7	0.035	0.23 (0.11)	$4.5x10^{-02}$	0.20 (0.13)	$1.3x10^{-01}$	0.22 (0.087)	$1.3x10^{-02}$
PLEKHB1	11q13.4	43	0.14	-0.083 (0.042)	$4.7x10^{-02}$	-0.077 (0.052)	$1.4x10^{-01}$	-0.081 (0.033)	$1.3x10^{-02}$
NPW	16p13.3	21	0.014	-0.49 (0.14)	$5.8x10^{-04}$	0.053 (0.18)	$7.6x10^{-01}$	-0.27 (0.11)	$1.4x10^{-02}$
UGP2	2p15	12	0.0065	-0.45 (0.14)	$1.7x10^{-03}$	0.050 (0.21)	$8.1x10^{-01}$	-0.29 (0.12)	$1.4x10^{-02}$
CCDC114	19q13.33	27	0.32	0.085 (0.032)	$7.3x10^{-03}$	0.024 (0.038)	$5.2x10^{-01}$	0.060 (0.024)	$1.4x10^{-02}$
FKBP11	12q13.12	17	0.031	-0.26 (0.10)	$9.4x10^{-03}$	-0.086 (0.12)	$4.8x10^{-01}$	-0.19 (0.077)	$1.4x10^{-02}$
PTPLAD2	9p21.3	37	0.087	0.16 (0.062)	$1.1x10^{-02}$	0.060 (0.074)	$4.2x10^{-01}$	0.12 (0.048)	$1.4x10^{-02}$
PDE9A	21q22.3	97	0.34	0.075 (0.031)	$1.7x10^{-02}$	0.035 (0.032)	$2.8x10^{-01}$	0.055 (0.022)	$1.4x10^{-02}$
CA6	1p36.23	42	0.33	0.051 (0.023)	$2.6x10^{-02}$	0.037 (0.035)	$2.9x10^{-01}$	0.047 (0.019)	$1.4x10^{-02}$
BCS1L	2q35	8	0.0051	0.65 (0.29)	$2.8x10^{-02}$	0.43 (0.37)	$2.4x10^{-01}$	0.56 (0.23)	$1.4x10^{-02}$
FDXR	17q25.1	33	0.12	0.090 (0.046)	$4.9x10^{-02}$	0.089 (0.060)	$1.4x10^{-01}$	0.090 (0.037)	$1.4x10^{-02}$
PEX14	1p36.22	18	0.066	0.23 (0.082)	$5.0x10^{-03}$	0.045 (0.092)	$6.2x10^{-01}$	0.15 (0.061)	$1.5x10^{-02}$
FAIM3	1q32.1	4	0.016	0.44 (0.17)	$9.1x10^{-03}$	0.14 (0.18)	$4.5x10^{-01}$	0.30 (0.12)	$1.5x10^{-02}$
PPP1R13L	19q13.32	33	0.044	-0.21 (0.087)	$1.4x10^{-02}$	-0.086 (0.10)	$4.0x10^{-01}$	-0.16 (0.066)	$1.5x10^{-02}$
MXD4	4p16.3	14	0.013	-0.26 (0.11)	$2.2x10^{-02}$	-0.17 (0.19)	$3.5x10^{-01}$	-0.24 (0.097)	$1.5x10^{-02}$
HIAT1	1p21.2	3	0.0054	-0.71 (0.34)	$3.8x10^{-02}$	-0.43 (0.30)	$1.6x10^{-01}$	-0.55 (0.23)	$1.5x10^{-02}$
BAG3	10q26.11	18	0.0051	-0.71 (0.20)	$4.9x10^{-04}$	0.22 (0.29)	$4.4x10^{-01}$	-0.40 (0.17)	$1.6x10^{-02}$
ZNF860	3p23	10	0.016	0.33 (0.13)	$9.9x10^{-03}$	0.085 (0.21)	$6.8x10^{-01}$	0.26 (0.11)	$1.6x10^{-02}$
TANC2	17q23.2	31	0.2	0.089 (0.040)	$2.5x10^{-02}$	0.048 (0.044)	$2.7x10^{-01}$	0.071 (0.029)	$1.6x10^{-02}$
RTDR1	22q11.22	19	0.053	0.16 (0.074)	$2.8x10^{-02}$	0.11 (0.100)	$2.7x10^{-01}$	0.14 (0.060)	$1.6x10^{-02}$
AKNA	9q32	9	0.074	-0.19 (0.088)	$3.3x10^{-02}$	-0.13 (0.10)	$2.3x10^{-01}$	-0.16 (0.068)	$1.6x10^{-02}$
PTPRJ	11p11.2	34	0.16	0.080 (0.038)	$3.6x10^{-02}$	0.063 (0.052)	$2.2x10^{-01}$	0.074 (0.031)	$1.6x10^{-02}$
ZNF576	19q13.31	37	0.052	-0.24 (0.068)	$4.5x10^{-04}$	0.056 (0.088)	$5.3x10^{-01}$	-0.13 (0.054)	$1.7x10^{-02}$
SYDE2	1p22.3	11	0.018	-0.34 (0.13)	$8.6x10^{-03}$	-0.083 (0.17)	$6.2x10^{-01}$	-0.24 (0.10)	$1.7x10^{-02}$

Gene	Location[a]	# SNPs in Prediction	Imputation Quality[b]	Discovery U4C Meta-analysis Beta (SE)	Discovery U4C Meta-analysis *p*-value	UK Biobank Replication Beta (SE)	UK Biobank Replication *p*-value	Joint Meta-analysis Beta (SE)	Joint Meta-analysis *p*-value
QDPR	4p15.32	6	0.65	-0.050 (0.020)	$1.2x10^{-02}$	-0.017 (0.025)	$4.9x10^{-01}$	-0.037 (0.016)	$1.7x10^{-02}$
STK16	2q35	27	0.0097	0.32 (0.13)	$1.8x10^{-02}$	0.15 (0.17)	$3.9x10^{-01}$	0.25 (0.11)	$1.7x10^{-02}$
PHOSPHO2	2q31.1	31	0.12	-0.10 (0.047)	$3.3x10^{-02}$	-0.069 (0.060)	$2.5x10^{-01}$	-0.089 (0.037)	$1.7x10^{-02}$
ARRDC2	19p13.11	20	0.034	-0.21 (0.100)	$3.7x10^{-02}$	-0.18 (0.15)	$2.4x10^{-01}$	-0.20 (0.083)	$1.7x10^{-02}$
NDUFA7	19p13.2	34	0.095	-0.13 (0.063)	$4.2x10^{-02}$	-0.091 (0.069)	$1.9x10^{-01}$	-0.11 (0.047)	$1.7x10^{-02}$
GPR19	12p13.1	8	0.26	-0.12 (0.041)	$3.2x10^{-03}$	-0.014 (0.044)	$7.6x10^{-01}$	-0.071 (0.030)	$1.8x10^{-02}$
MCM9	6q22.31	76	0.094	0.11 (0.047)	$2.2x10^{-02}$	0.052 (0.053)	$3.3x10^{-01}$	0.084 (0.035)	$1.8x10^{-02}$
NOL9	1p36.31	19	0.081	0.096 (0.048)	$4.5x10^{-02}$	0.12 (0.095)	$2.0x10^{-01}$	0.10 (0.043)	$1.8x10^{-02}$
IDH1	2q34	6	0.011	0.59 (0.20)	$3.1x10^{-03}$	0.034 (0.23)	$8.8x10^{-01}$	0.35 (0.15)	$1.9x10^{-02}$
PMFBP1	16q22.2	39	0.012	-0.21 (0.082)	$1.1x10^{-02}$	-0.051 (0.12)	$6.6x10^{-01}$	-0.16 (0.067)	$1.9x10^{-02}$
TRIM16	17p12	32	0.21	-0.095 (0.037)	$1.1x10^{-02}$	-0.027 (0.046)	$5.6x10^{-01}$	-0.068 (0.029)	$1.9x10^{-02}$
ARID5B	10q21.2	29	0.11	0.14 (0.061)	$2.5x10^{-02}$	0.069 (0.068)	$3.1x10^{-01}$	0.11 (0.045)	$1.9x10^{-02}$
QSOX1	1q25.2	55	0.45	-0.043 (0.021)	$4.4x10^{-02}$	-0.035 (0.029)	$2.2x10^{-01}$	-0.040 (0.017)	$1.9x10^{-02}$
SYNPO2	4q26	30	0.2	0.11 (0.040)	$4.4x10^{-03}$	0.012 (0.046)	$7.9x10^{-01}$	0.070 (0.030)	$2.0x10^{-02}$
TAP2	6p21.32	56	0.56	0.054 (0.023)	$1.9x10^{-02}$	0.023 (0.029)	$4.3x10^{-01}$	0.042 (0.018)	$2.0x10^{-02}$
POLR3G	5q14.3	19	0.16	-0.11 (0.047)	$2.2x10^{-02}$	-0.049 (0.054)	$3.7x10^{-01}$	-0.083 (0.036)	$2.0x10^{-02}$
ABCC10	6p21.1	22	0.054	-0.19 (0.091)	$3.2x10^{-02}$	-0.12 (0.11)	$2.8x10^{-01}$	-0.16 (0.069)	$2.0x10^{-02}$
CCDC53	12q23.2	26	0.35	0.050 (0.025)	$4.4x10^{-02}$	0.041 (0.035)	$2.4x10^{-01}$	0.047 (0.020)	$2.0x10^{-02}$
TRAF3IP2	6q21	22	0.12	0.088 (0.045)	$4.8x10^{-02}$	0.071 (0.058)	$2.2x10^{-01}$	0.082 (0.035)	$2.0x10^{-02}$
MS4A14	11q12.2	33	0.23	0.10 (0.036)	$4.6x10^{-03}$	0.009 (0.043)	$8.3x10^{-01}$	0.064 (0.028)	$2.1x10^{-02}$
ZNF165	6p22.1	21	0.039	-0.20 (0.078)	$8.3x10^{-03}$	-0.033 (0.11)	$7.5x10^{-01}$	-0.14 (0.062)	$2.1x10^{-02}$
XBP1	22q12.1	39	0.25	0.082 (0.032)	$1.2x10^{-02}$	0.021 (0.042)	$6.1x10^{-01}$	0.059 (0.026)	$2.1x10^{-02}$
SLC5A11	16p12.1	34	0.75	-0.051 (0.021)	$1.5x10^{-02}$	-0.017 (0.023)	$4.6x10^{-01}$	-0.036 (0.016)	$2.1x10^{-02}$
FAM114A1	4p14	26	0.077	0.17 (0.071)	$1.6x10^{-02}$	0.061 (0.087)	$4.8x10^{-01}$	0.13 (0.055)	$2.1x10^{-02}$
NDUFA10	2q37.3	46	0.52	0.053 (0.023)	$2.1x10^{-02}$	0.023 (0.028)	$4.1x10^{-01}$	0.041 (0.018)	$2.1x10^{-02}$
BLMH	17q11.2	44	0.14	0.092 (0.041)	$2.4x10^{-02}$	0.043 (0.048)	$3.7x10^{-01}$	0.071 (0.031)	$2.1x10^{-02}$
PDS5B	13q13.1	33	0.017	-0.26 (0.12)	$2.7x10^{-02}$	-0.14 (0.15)	$3.6x10^{-01}$	-0.21 (0.092)	$2.1x10^{-02}$

Gene	Location[a]	# SNPs in Prediction	Imputation Quality[b]	Discovery U4C Meta-analysis Beta (SE)	Discovery U4C Meta-analysis *p*-value	UK Biobank Replication Beta (SE)	UK Biobank Replication *p*-value	Joint Meta-analysis Beta (SE)	Joint Meta-analysis *p*-value
DFFA	1p36.22	6	0.0067	0.50 (0.23)	$3.1x10^{-02}$	0.29 (0.29)	$3.1x10^{-01}$	0.42 (0.18)	$2.1x10^{-02}$
CCDC132	7q21.3	7	0.022	-0.32 (0.16)	$4.4x10^{-02}$	-0.22 (0.18)	$2.4x10^{-01}$	-0.28 (0.12)	$2.1x10^{-02}$
NUP210	3p25.1	17	0.033	-0.27 (0.10)	$1.0x10^{-02}$	-0.056 (0.13)	$6.8x10^{-01}$	-0.19 (0.082)	$2.2x10^{-02}$
FKBP7	2q31.2	28	0.034	0.24 (0.074)	$1.2x10^{-03}$	-0.068 (0.10)	$5.2x10^{-01}$	0.14 (0.061)	$2.3x10^{-02}$
NSMCE1	16p12.1	26	0.067	-0.19 (0.076)	$1.3x10^{-02}$	-0.054 (0.090)	$5.5x10^{-01}$	-0.13 (0.058)	$2.3x10^{-02}$
MRPL43	10q24.31	17	0.41	-0.049 (0.022)	$2.3x10^{-02}$	-0.023 (0.033)	$4.9x10^{-01}$	-0.041 (0.018)	$2.3x10^{-02}$
LZTS1	8p21.3	56	0.15	-0.079 (0.038)	$3.6x10^{-02}$	-0.052 (0.054)	$3.4x10^{-01}$	-0.070 (0.031)	$2.3x10^{-02}$
SOCS6	18q22.2	8	0.0066	-0.40 (0.20)	$4.6x10^{-02}$	-0.31 (0.27)	$2.6x10^{-01}$	-0.37 (0.16)	$2.3x10^{-02}$
ZNF707	8q24.3	16	0.11	0.085 (0.043)	$4.9x10^{-02}$	0.075 (0.066)	$2.5x10^{-01}$	0.082 (0.036)	$2.3x10^{-02}$
CRYM	16p12.2	40	0.12	-0.12 (0.054)	$2.6x10^{-02}$	-0.053 (0.056)	$3.4x10^{-01}$	-0.088 (0.039)	$2.4x10^{-02}$
CNOT6L	4q21.1	13	0.0061	-0.42 (0.16)	$1.2x10^{-02}$	-0.056 (0.25)	$8.3x10^{-01}$	-0.31 (0.14)	$2.5x10^{-02}$
C10orf2	10q24.31	16	0.022	0.31 (0.12)	$1.2x10^{-02}$	0.062 (0.16)	$6.9x10^{-01}$	0.22 (0.097)	$2.5x10^{-02}$
MSLN	16p13.3	137	0.37	-0.060 (0.026)	$2.1x10^{-02}$	-0.023 (0.032)	$4.7x10^{-01}$	-0.045 (0.020)	$2.5x10^{-02}$
TMEM62	15q15.2	6	0.014	0.33 (0.16)	$4.0x10^{-02}$	0.20 (0.20)	$3.1x10^{-01}$	0.28 (0.12)	$2.5x10^{-02}$
SRGAP2	1q32.1	61	0.012	0.17 (0.064)	$6.2x10^{-03}$	0.000 (0.090)	1.00E+00	0.12 (0.052)	$2.6x10^{-02}$
ALCAM	3q13.11	77	0.28	0.064 (0.025)	$1.1x10^{-02}$	0.009 (0.036)	$8.1x10^{-01}$	0.046 (0.021)	$2.6x10^{-02}$
PCBP3	21q22.3	73	0.39	0.060 (0.027)	$2.3x10^{-02}$	0.024 (0.031)	$4.5x10^{-01}$	0.045 (0.020)	$2.6x10^{-02}$
RPL7L1	6p21.1	16	0.09	0.15 (0.070)	$3.1x10^{-02}$	0.072 (0.075)	$3.4x10^{-01}$	0.11 (0.051)	$2.6x10^{-02}$
UNG	12q24.11	4	0.23	-0.075 (0.037)	$4.5x10^{-02}$	-0.050 (0.048)	$2.9x10^{-01}$	-0.065 (0.029)	$2.6x10^{-02}$
CNKSR3	6q25.2	28	0.17	-0.11 (0.043)	$7.4x10^{-03}$	-0.009 (0.054)	$8.7x10^{-01}$	-0.074 (0.034)	$2.7x10^{-02}$
SAT2	17p13.1	32	0.24	0.10 (0.040)	$1.2x10^{-02}$	0.023 (0.042)	$5.9x10^{-01}$	0.064 (0.029)	$2.7x10^{-02}$
ITGA7	12q13.2	43	0.1	-0.11 (0.049)	$3.1x10^{-02}$	-0.053 (0.066)	$4.2x10^{-01}$	-0.087 (0.039)	$2.7x10^{-02}$
CCND1	11q13.3	29	0.083	0.15 (0.072)	$3.7x10^{-02}$	0.077 (0.077)	$3.2x10^{-01}$	0.12 (0.053)	$2.7x10^{-02}$
ARL4A	7p21.3	47	0.06	-0.20 (0.073)	$5.3x10^{-03}$	-0.014 (0.082)	$8.7x10^{-01}$	-0.12 (0.055)	$2.8x10^{-02}$
SHISA4	1q32.1	33	0.045	0.22 (0.099)	$2.9x10^{-02}$	0.094 (0.11)	$4.0x10^{-01}$	0.16 (0.075)	$2.8x10^{-02}$
PDCD11	10q24.33	42	0.034	-0.20 (0.071)	$5.7x10^{-03}$	-0.004 (0.089)	$9.7x10^{-01}$	-0.12 (0.056)	$2.9x10^{-02}$
TMEM170B	6p24.2	41	0.045	0.18 (0.084)	$3.7x10^{-02}$	0.091 (0.10)	$3.8x10^{-01}$	0.14 (0.066)	$2.9x10^{-02}$

Gene	Location[a]	# SNPs in Prediction	Imputation Quality[b]	Discovery U4C Meta-analysis Beta (SE)	Discovery U4C Meta-analysis *p*-value	UK Biobank Replication Beta (SE)	UK Biobank Replication *p*-value	Joint Meta-analysis Beta (SE)	Joint Meta-analysis *p*-value
TDG	12q23.3	19	0.0082	-0.36 (0.15)	$1.9x10^{-02}$	-0.11 (0.17)	$5.4x10^{-01}$	-0.25 (0.11)	$3.0x10^{-02}$
RNF157	17q25.1	21	0.094	0.14 (0.059)	$2.1x10^{-02}$	0.045 (0.074)	$5.5x10^{-01}$	0.10 (0.046)	$3.0x10^{-02}$
DCBLD2	3q12.1	32	0.17	0.090 (0.044)	$4.0x10^{-02}$	0.049 (0.054)	$3.6x10^{-01}$	0.074 (0.034)	$3.0x10^{-02}$
SCUBE2	11p15.4	25	0.035	-0.15 (0.078)	$4.9x10^{-02}$	-0.10 (0.11)	$3.3x10^{-01}$	-0.14 (0.063)	$3.0x10^{-02}$
PLD6	17p11.2	34	0.25	-0.054 (0.021)	$1.0x10^{-02}$	0.011 (0.041)	$7.9x10^{-01}$	-0.040 (0.019)	$3.1x10^{-02}$
FARS2	6p25.1	60	0.17	-0.089 (0.035)	$1.1x10^{-02}$	-0.007 (0.047)	$8.8x10^{-01}$	-0.060 (0.028)	$3.1x10^{-02}$
HBE1	11p15.4	8	0.016	-0.32 (0.14)	$2.2x10^{-02}$	-0.088 (0.22)	$6.9x10^{-01}$	-0.25 (0.12)	$3.1x10^{-02}$
ABCG1	21q22.3	33	0.0048	0.34 (0.16)	$3.8x10^{-02}$	0.18 (0.22)	$4.2x10^{-01}$	0.28 (0.13)	$3.1x10^{-02}$
IRGQ	19q13.31	22	0.052	-0.21 (0.072)	$3.0x10^{-03}$	0.028 (0.091)	$7.6x10^{-01}$	-0.12 (0.057)	$3.2x10^{-02}$
MAP1LC3A	20q11.22	49	0.27	-0.088 (0.031)	$4.7x10^{-03}$	-0.002 (0.035)	$9.6x10^{-01}$	-0.050 (0.023)	$3.2x10^{-02}$
AHNAK	11q12.3	57	0.038	0.19 (0.076)	$1.0x10^{-02}$	0.027 (0.090)	$7.6x10^{-01}$	0.12 (0.058)	$3.2x10^{-02}$
ATE1	10q26.13	4	0.011	-0.52 (0.25)	$3.4x10^{-02}$	-0.22 (0.24)	$3.6x10^{-01}$	-0.37 (0.17)	$3.2x10^{-02}$
TAF11	6p21.31	29	0.0077	-0.20 (0.098)	$3.7x10^{-02}$	-0.11 (0.14)	$4.4x10^{-01}$	-0.17 (0.080)	$3.2x10^{-02}$
AGPHD1	15q25.1	12	0.017	-0.31 (0.15)	$3.7x10^{-02}$	-0.14 (0.16)	$3.6x10^{-01}$	-0.23 (0.11)	$3.2x10^{-02}$
KISS1R	19p13.3	14	0.22	0.098 (0.049)	$4.8x10^{-02}$	0.051 (0.047)	$2.8x10^{-01}$	0.073 (0.034)	$3.2x10^{-02}$
PAK1	11q14.1	35	0.058	0.26 (0.093)	$4.5x10^{-03}$	0.018 (0.091)	$8.4x10^{-01}$	0.14 (0.065)	$3.3x10^{-02}$
RSPH9	6p21.1	50	0.009	0.28 (0.11)	$8.6x10^{-03}$	0.011 (0.14)	$9.3x10^{-01}$	0.18 (0.084)	$3.3x10^{-02}$
IFI16	1q23.1	14	0.019	0.30 (0.12)	$1.1x10^{-02}$	0.000 (0.19)	1.00E+00	0.21 (0.10)	$3.3x10^{-02}$
CSDA	17p13.1	64	0.033	0.14 (0.061)	$2.2x10^{-02}$	0.039 (0.090)	$6.7x10^{-01}$	0.11 (0.051)	$3.3x10^{-02}$
PDZD8	10q26.11	64	0.44	-0.060 (0.027)	$2.7x10^{-02}$	-0.022 (0.030)	$4.6x10^{-01}$	-0.043 (0.020)	$3.3x10^{-02}$
LSR	19q13.12	18	0.17	0.10 (0.046)	$2.8x10^{-02}$	0.039 (0.056)	$4.9x10^{-01}$	0.076 (0.036)	$3.3x10^{-02}$
C2	6p21.33	45	0.085	-0.21 (0.068)	$1.9x10^{-03}$	-0.002 (0.062)	$9.7x10^{-01}$	-0.097 (0.046)	$3.4x10^{-02}$
B3GALTL	13q12.3	53	0.034	0.20 (0.075)	$6.7x10^{-03}$	-0.024 (0.11)	$8.3x10^{-01}$	0.13 (0.062)	$3.4x10^{-02}$
BIK	22q13.2	40	0.068	0.20 (0.075)	$7.7x10^{-03}$	0.010 (0.090)	$9.2x10^{-01}$	0.12 (0.058)	$3.4x10^{-02}$
OCIAD2	4p11	1	0.0064	-0.59 (0.23)	$1.0x10^{-02}$	0.097 (0.41)	$8.1x10^{-01}$	-0.42 (0.20)	$3.4x10^{-02}$
ZDHHC5	11q12.1	16	0.084	0.17 (0.073)	$2.0x10^{-02}$	0.048 (0.081)	$5.5x10^{-01}$	0.12 (0.054)	$3.4x10^{-02}$
IDO1	8p11.21	37	0.13	-0.093 (0.045)	$3.8x10^{-02}$	-0.046 (0.059)	$4.4x10^{-01}$	-0.076 (0.036)	$3.4x10^{-02}$

Gene	Location[a]	# SNPs in Prediction	Imputation Quality[b]	Discovery U4C Meta-analysis		UK Biobank Replication		Joint Meta-analysis	
				Beta (SE)	*p*-value	Beta (SE)	*p*-value	Beta (SE)	*p*-value
CDKN1A	6p21.2	44	0.052	0.16 (0.065)	$1.3x10^{-02}$	0.020 (0.082)	$8.1x10^{-01}$	0.11 (0.051)	$3.5x10^{-02}$
UBE2E1	3p24.3	31	0.0064	0.23 (0.11)	$3.6x10^{-02}$	0.10 (0.15)	$4.8x10^{-01}$	0.18 (0.087)	$3.5x10^{-02}$
ACSL5	10q25.2	21	0.38	0.052 (0.026)	$4.4x10^{-02}$	0.029 (0.034)	$4.0x10^{-01}$	0.044 (0.021)	$3.5x10^{-02}$
TRPV5	7q34	32	0.22	-0.11 (0.043)	$1.0x10^{-02}$	-0.016 (0.044)	$7.2x10^{-01}$	-0.065 (0.031)	$3.6x10^{-02}$
CASC1	12p12.1	33	0.23	0.094 (0.038)	$1.2x10^{-02}$	0.011 (0.047)	$8.1x10^{-01}$	0.061 (0.029)	$3.6x10^{-02}$
AFMID	17q25.3	3	0.035	0.26 (0.098)	$7.7x10^{-03}$	-0.031 (0.15)	$8.3x10^{-01}$	0.17 (0.081)	$3.7x10^{-02}$
CD44	11p13	8	0.049	0.29 (0.12)	$1.1x10^{-02}$	0.031 (0.14)	$8.2x10^{-01}$	0.18 (0.088)	$3.7x10^{-02}$
TMEM184B	22q13.1	19	0.15	0.12 (0.050)	$2.1x10^{-02}$	0.029 (0.060)	$6.2x10^{-01}$	0.080 (0.038)	$3.7x10^{-02}$
CHPF2	7q36.1	45	0.07	0.13 (0.064)	$3.6x10^{-02}$	0.057 (0.074)	$4.4x10^{-01}$	0.10 (0.048)	$3.7x10^{-02}$
PLAT	8p11.21	8	0.067	-0.14 (0.072)	$4.6x10^{-02}$	-0.079 (0.097)	$4.2x10^{-01}$	-0.12 (0.058)	$3.7x10^{-02}$
NOG	17q22	36	0.069	0.18 (0.073)	$1.3x10^{-02}$	0.029 (0.082)	$7.2x10^{-01}$	0.11 (0.054)	$3.8x10^{-02}$
ANKRD26	10p12.1	89	0.077	0.096 (0.044)	$2.9x10^{-02}$	0.032 (0.062)	$6.1x10^{-01}$	0.074 (0.036)	$3.8x10^{-02}$
CYB561	17q23.3	11	0.014	-0.34 (0.17)	$4.9x10^{-02}$	-0.18 (0.20)	$3.7x10^{-01}$	-0.28 (0.13)	$3.8x10^{-02}$
WDR37	10p15.3	9	0.033	-0.28 (0.10)	$5.9x10^{-03}$	0.075 (0.16)	$6.4x10^{-01}$	-0.18 (0.086)	$3.9x10^{-02}$
ISOC1	5q23.3	42	0.32	0.061 (0.027)	$2.2x10^{-02}$	0.013 (0.036)	$7.1x10^{-01}$	0.044 (0.022)	$3.9x10^{-02}$
RNF216	7p22.1	41	0.059	-0.12 (0.054)	$2.6x10^{-02}$	-0.035 (0.074)	$6.4x10^{-01}$	-0.091 (0.044)	$3.9x10^{-02}$
CASC3	17q21.1	36	0.024	-0.12 (0.058)	$4.0x10^{-02}$	-0.057 (0.096)	$5.5x10^{-01}$	-0.10 (0.050)	$3.9x10^{-02}$
TNS4	17q21.2	6	0.031	0.27 (0.13)	$4.3x10^{-02}$	0.12 (0.15)	$4.1x10^{-01}$	0.21 (0.099)	$3.9x10^{-02}$
CCDC126	7p15.3	13	0.16	0.11 (0.057)	$4.9x10^{-02}$	0.054 (0.055)	$3.3x10^{-01}$	0.082 (0.040)	$3.9x10^{-02}$
TPBG	6q14.1	73	0.098	-0.098 (0.043)	$2.2x10^{-02}$	-0.022 (0.056)	$7.0x10^{-01}$	-0.070 (0.034)	$4.0x10^{-02}$
THNSL1	10p12.1	40	0.031	0.17 (0.086)	$4.6x10^{-02}$	0.086 (0.11)	$4.3x10^{-01}$	0.14 (0.068)	$4.0x10^{-02}$
MCCC1	3q27.1	37	0.12	0.11 (0.041)	$1.1x10^{-02}$	-0.005 (0.059)	$9.4x10^{-01}$	0.069 (0.034)	$4.1x10^{-02}$
TMED6	16q22.1	25	0.31	0.064 (0.032)	$4.7x10^{-02}$	0.030 (0.036)	$4.0x10^{-01}$	0.049 (0.024)	$4.1x10^{-02}$
MAFF	22q13.1	14	0.059	-0.19 (0.075)	$1.3x10^{-02}$	-0.006 (0.10)	$9.5x10^{-01}$	-0.12 (0.060)	$4.2x10^{-02}$
NME4	16p13.3	47	0.5	-0.052 (0.024)	$2.8x10^{-02}$	-0.015 (0.028)	$5.8x10^{-01}$	-0.037 (0.018)	$4.2x10^{-02}$
NDUFB1	14q32.12	10	0.011	0.50 (0.24)	$3.8x10^{-02}$	0.19 (0.29)	$5.1x10^{-01}$	0.38 (0.18)	$4.2x10^{-02}$
TM2D3	15q26.3	13	0.039	-0.21 (0.10)	$3.8x10^{-02}$	-0.083 (0.12)	$4.7x10^{-01}$	-0.16 (0.076)	$4.2x10^{-02}$

Gene	Location[a]	# SNPs in Prediction	Imputation Quality[b]	Discovery U4C Meta-analysis Beta (SE)	Discovery U4C Meta-analysis *p*-value	UK Biobank Replication Beta (SE)	UK Biobank Replication *p*-value	Joint Meta-analysis Beta (SE)	Joint Meta-analysis *p*-value
SPRYD3	12q13.13	14	0.06	-0.18 (0.071)	$1.2x10^{-02}$	0.024 (0.11)	$8.3x10^{-01}$	-0.12 (0.060)	$4.3x10^{-02}$
STX1B	16p11.2	23	0.045	-0.20 (0.087)	$2.4x10^{-02}$	-0.050 (0.096)	$6.0x10^{-01}$	-0.13 (0.064)	$4.3x10^{-02}$
SLK	10q24.33	32	0.08	0.12 (0.061)	$4.6x10^{-02}$	0.058 (0.078)	$4.6x10^{-01}$	0.098 (0.048)	$4.3x10^{-02}$
POLR3D	8p21.3	8	0.093	0.17 (0.074)	$2.4x10^{-02}$	0.041 (0.081)	$6.1x10^{-01}$	0.11 (0.055)	$4.4x10^{-02}$
GNA13	17q24.1	7	0.012	-0.37 (0.18)	$3.7x10^{-02}$	-0.13 (0.26)	$6.1x10^{-01}$	-0.29 (0.15)	$4.4x10^{-02}$
VIL1	2q35	6	0.084	0.15 (0.072)	$3.7x10^{-02}$	0.056 (0.078)	$4.8x10^{-01}$	0.11 (0.053)	$4.4x10^{-02}$
C16orf62	16p12.3	11	0.021	0.27 (0.12)	$2.5x10^{-02}$	0.068 (0.14)	$6.3x10^{-01}$	0.18 (0.091)	$4.5x10^{-02}$
HLA-DOB	6p21.32	51	0.47	-0.058 (0.025)	$1.8x10^{-02}$	-0.009 (0.030)	$7.7x10^{-01}$	-0.038 (0.019)	$4.6x10^{-02}$
CNNM2	10q24.32	11	0.0076	-0.35 (0.16)	$2.7x10^{-02}$	-0.080 (0.22)	$7.1x10^{-01}$	-0.26 (0.13)	$4.6x10^{-02}$
NKD1	16q12.1	49	0.075	-0.13 (0.053)	$1.8x10^{-02}$	-0.007 (0.075)	$9.3x10^{-01}$	-0.086 (0.043)	$4.7x10^{-02}$
CTAGE5	14q21.1	15	0.023	-0.29 (0.11)	$8.4x10^{-03}$	0.034 (0.15)	$8.2x10^{-01}$	-0.18 (0.089)	$4.8x10^{-02}$
KRT74	12q13.13	51	0.3	-0.076 (0.030)	$1.2x10^{-02}$	0.000 (0.039)	1.00E+00	-0.048 (0.024)	$4.8x10^{-02}$
GCOM1	15q21.3	75	0.67	-0.055 (0.020)	$4.8x10^{-03}$	0.007 (0.023)	$7.6x10^{-01}$	-0.030 (0.015)	$4.9x10^{-02}$
PRSS50	3p21.31	18	0.1	0.097 (0.047)	$4.1x10^{-02}$	0.033 (0.073)	$6.5x10^{-01}$	0.078 (0.040)	$4.9x10^{-02}$
KRT73	12q13.13	53	0.53	-0.052 (0.022)	$1.6x10^{-02}$	-0.001 (0.029)	$9.8x10^{-01}$	-0.034 (0.017)	$5.1x10^{-02}$
MPP2	17q21.31	27	0.39	0.051 (0.025)	$4.1x10^{-02}$	0.018 (0.034)	$6.0x10^{-01}$	0.039 (0.020)	$5.1x10^{-02}$
SCUBE3	6p21.31	31	0.049	0.20 (0.080)	$1.5x10^{-02}$	-0.005 (0.11)	$9.7x10^{-01}$	0.13 (0.065)	$5.2x10^{-02}$
PLIN3	19p13.3	15	0.0085	-0.34 (0.15)	$2.0x10^{-02}$	-0.042 (0.18)	$8.1x10^{-01}$	-0.22 (0.11)	$5.2x10^{-02}$
RRP1	21q22.3	51	0.12	0.11 (0.049)	$2.8x10^{-02}$	0.023 (0.059)	$7.0x10^{-01}$	0.073 (0.038)	$5.2x10^{-02}$
ADCY7	16q12.1	26	0.31	-0.051 (0.024)	$3.2x10^{-02}$	-0.009 (0.038)	$8.2x10^{-01}$	-0.039 (0.020)	$5.2x10^{-02}$
C12orf57	12p13.31	41	0.015	0.22 (0.11)	$3.5x10^{-02}$	0.035 (0.20)	$8.6x10^{-01}$	0.18 (0.094)	$5.2x10^{-02}$
GNPTAB	12q23.2	14	0.021	-0.23 (0.11)	$4.2x10^{-02}$	-0.077 (0.17)	$6.4x10^{-01}$	-0.18 (0.094)	$5.2x10^{-02}$
CHD7	8q12.1	25	0.059	-0.21 (0.087)	$1.3x10^{-02}$	-0.015 (0.094)	$8.7x10^{-01}$	-0.12 (0.064)	$5.3x10^{-02}$
CHD5	1p36.31	34	0.057	-0.18 (0.083)	$2.7x10^{-02}$	-0.041 (0.092)	$6.6x10^{-01}$	-0.12 (0.062)	$5.3x10^{-02}$
LILRB4	19q13.42	81	0.25	0.067 (0.033)	$4.5x10^{-02}$	0.024 (0.041)	$5.5x10^{-01}$	0.050 (0.026)	$5.3x10^{-02}$
TMEM200A	6q23.1	37	0.055	-0.17 (0.069)	$1.1x10^{-02}$	0.009 (0.087)	$9.2x10^{-01}$	-0.10 (0.054)	$5.4x10^{-02}$
NUDT19	19q13.11	36	0.05	-0.17 (0.062)	$6.9x10^{-03}$	0.052 (0.091)	$5.7x10^{-01}$	-0.098 (0.051)	$5.5x10^{-02}$

Gene	Location[a]	# SNPs in Prediction	Imputation Quality[b]	Discovery U4C Meta-analysis Beta (SE)	Discovery U4C Meta-analysis *p*-value	UK Biobank Replication Beta (SE)	UK Biobank Replication *p*-value	Joint Meta-analysis Beta (SE)	Joint Meta-analysis *p*-value
BRD7	16q12.1	20	0.32	-0.054 (0.024)	$2.2x10^{-02}$	0.002 (0.038)	$9.6x10^{-01}$	-0.039 (0.020)	$5.5x10^{-02}$
GLYCTK	3p21.1	25	0.24	0.077 (0.034)	$2.2x10^{-02}$	0.010 (0.041)	$8.1x10^{-01}$	0.050 (0.026)	$5.5x10^{-02}$
ZNF575	19q13.31	25	0.37	-0.10 (0.029)	$5.6x10^{-04}$	0.035 (0.033)	$2.9x10^{-01}$	-0.042 (0.022)	$5.6x10^{-02}$
FYTTD1	3q29	7	0.0054	-0.58 (0.21)	$6.7x10^{-03}$	0.16 (0.30)	$6.0x10^{-01}$	-0.34 (0.18)	$5.6x10^{-02}$
RRP9	3p21.2	17	0.0061	0.32 (0.13)	$1.5x10^{-02}$	-0.038 (0.20)	$8.5x10^{-01}$	0.21 (0.11)	$5.6x10^{-02}$
USP28	11q23.2	19	0.013	0.41 (0.20)	$3.6x10^{-02}$	0.12 (0.20)	$5.4x10^{-01}$	0.27 (0.14)	$5.6x10^{-02}$
GEMIN4	17p13.3	16	0.086	0.11 (0.055)	$4.1x10^{-02}$	0.031 (0.082)	$7.0x10^{-01}$	0.087 (0.046)	$5.6x10^{-02}$
AGMAT	1p36.21	16	0.0077	0.40 (0.20)	$4.7x10^{-02}$	0.14 (0.24)	$5.6x10^{-01}$	0.30 (0.16)	$5.6x10^{-02}$
CHEK2	22q12.1	23	0.045	0.17 (0.072)	$2.1x10^{-02}$	0.009 (0.096)	$9.2x10^{-01}$	0.11 (0.058)	$5.7x10^{-02}$
MRI1	19p13.2	57	0.097	-0.11 (0.052)	$4.2x10^{-02}$	-0.032 (0.063)	$6.1x10^{-01}$	-0.076 (0.040)	$5.8x10^{-02}$
SNTB1	8q24.12	22	0.01	0.36 (0.16)	$2.2x10^{-02}$	0.019 (0.21)	$9.3x10^{-01}$	0.24 (0.13)	$5.9x10^{-02}$
ZNF410	14q24.3	11	0.023	0.19 (0.090)	$3.3x10^{-02}$	0.008 (0.16)	$9.6x10^{-01}$	0.15 (0.079)	$5.9x10^{-02}$
TRDMT1	10p13	29	0.065	-0.15 (0.070)	$3.2x10^{-02}$	-0.026 (0.091)	$7.7x10^{-01}$	-0.10 (0.055)	$6.0x10^{-02}$
NUDT12	5q21.2	40	0.48	-0.053 (0.026)	$4.1x10^{-02}$	-0.016 (0.028)	$5.7x10^{-01}$	-0.036 (0.019)	$6.0x10^{-02}$
DNAL1	14q24.3	27	0.0096	0.40 (0.14)	$3.9x10^{-03}$	-0.047 (0.15)	$7.5x10^{-01}$	0.19 (0.10)	$6.1x10^{-02}$
CENPO	2p23.3	10	0.021	-0.27 (0.13)	$3.0x10^{-02}$	-0.056 (0.14)	$6.9x10^{-01}$	-0.18 (0.094)	$6.1x10^{-02}$
C7orf46	7p15.3	32	0.35	-0.053 (0.026)	$4.6x10^{-02}$	-0.016 (0.035)	$6.4x10^{-01}$	-0.039 (0.021)	$6.1x10^{-02}$
NOXO1	16p13.3	33	0.26	-0.16 (0.042)	$1.8x10^{-04}$	0.039 (0.040)	$3.2x10^{-01}$	-0.054 (0.029)	$6.2x10^{-02}$
HLA-DMA	6p21.32	31	0.059	-0.16 (0.063)	$1.1x10^{-02}$	0.006 (0.072)	$9.4x10^{-01}$	-0.088 (0.047)	$6.2x10^{-02}$
MS4A7	11q12.2	27	0.26	-0.081 (0.033)	$1.3x10^{-02}$	0.006 (0.042)	$8.9x10^{-01}$	-0.048 (0.026)	$6.2x10^{-02}$
DLK2	6p21.1	22	0.23	0.084 (0.036)	$1.9x10^{-02}$	0.004 (0.043)	$9.3x10^{-01}$	0.051 (0.028)	$6.2x10^{-02}$
SSX2IP	1p22.3	11	0.014	0.34 (0.16)	$2.8x10^{-02}$	0.036 (0.21)	$8.6x10^{-01}$	0.23 (0.12)	$6.2x10^{-02}$
RNF111	15q22.1	1	0.0078	4.80 (2.4)	$4.5x10^{-02}$	1.30 (3.5)	$7.2x10^{-01}$	3.70 (2.0)	$6.2x10^{-02}$
FAH	15q25.1	49	0.16	0.18 (0.054)	$1.0x10^{-03}$	-0.042 (0.056)	$4.6x10^{-01}$	0.073 (0.039)	$6.3x10^{-02}$
C7orf41	7p14.3	8	0.032	0.26 (0.12)	$2.3x10^{-02}$	0.017 (0.15)	$9.1x10^{-01}$	0.17 (0.091)	$6.4x10^{-02}$
GHRL	3p25.3	34	0.063	-0.19 (0.060)	$1.4x10^{-03}$	0.12 (0.086)	$1.6x10^{-01}$	-0.090 (0.049)	$6.6x10^{-02}$
DNAH1	3p21.1	16	0.051	0.14 (0.070)	$4.3x10^{-02}$	0.033 (0.097)	$7.4x10^{-01}$	0.11 (0.057)	$6.6x10^{-02}$

Gene	Location[a]	# SNPs in Prediction	Imputation Quality[b]	Discovery U4C Meta-analysis Beta (SE)	Discovery U4C Meta-analysis *p*-value	UK Biobank Replication Beta (SE)	UK Biobank Replication *p*-value	Joint Meta-analysis Beta (SE)	Joint Meta-analysis *p*-value
PLEKHA3	2q31.2	18	0.019	-0.29 (0.11)	$8.8x10^{-03}$	0.068 (0.15)	$6.5x10^{-01}$	-0.16 (0.088)	$6.7x10^{-02}$
H2AFZ	4q23	17	0.1	0.11 (0.055)	$4.4x10^{-02}$	0.026 (0.073)	$7.2x10^{-01}$	0.080 (0.044)	$6.7x10^{-02}$
SULF2	20q13.13	63	0.39	-0.099 (0.030)	$1.0x10^{-03}$	0.031 (0.034)	$3.5x10^{-01}$	-0.041 (0.023)	$6.8x10^{-02}$
PPP2R1A	19q13.41	34	0.011	0.35 (0.12)	$3.3x10^{-03}$	-0.13 (0.16)	$4.0x10^{-01}$	0.18 (0.096)	$6.8x10^{-02}$
KCNK17	6p21.2	32	0.71	-0.049 (0.020)	$1.3x10^{-02}$	0.003 (0.023)	$9.1x10^{-01}$	-0.028 (0.015)	$6.8x10^{-02}$
PHYH	10p13	24	0.21	0.13 (0.043)	$3.9x10^{-03}$	-0.025 (0.049)	$6.1x10^{-01}$	0.059 (0.032)	$6.9x10^{-02}$
SREK1IP1	5q12.3	18	0.029	0.28 (0.11)	$7.3x10^{-03}$	-0.072 (0.14)	$6.0x10^{-01}$	0.15 (0.083)	$7.0x10^{-02}$
THBS4	5q14.1	35	0.019	-0.29 (0.11)	$8.9x10^{-03}$	0.051 (0.14)	$7.1x10^{-01}$	-0.15 (0.086)	$7.0x10^{-02}$
LRRC45	17q25.3	18	0.24	-0.099 (0.037)	$8.0x10^{-03}$	0.015 (0.044)	$7.4x10^{-01}$	-0.052 (0.029)	$7.1x10^{-02}$
IER3	6p21.33	27	0.17	-0.11 (0.050)	$2.5x10^{-02}$	-0.019 (0.044)	$6.6x10^{-01}$	-0.059 (0.033)	$7.2x10^{-02}$
AMIGO1	1p13.3	24	0.19	-0.10 (0.045)	$2.6x10^{-02}$	-0.011 (0.050)	$8.3x10^{-01}$	-0.060 (0.033)	$7.2x10^{-02}$
CTNNA2	2p12	31	0.18	0.091 (0.042)	$2.9x10^{-02}$	0.009 (0.050)	$8.5x10^{-01}$	0.058 (0.032)	$7.2x10^{-02}$
RDX	11q22.3	26	0.033	-0.21 (0.081)	$1.1x10^{-02}$	0.030 (0.099)	$7.7x10^{-01}$	-0.11 (0.063)	$7.3x10^{-02}$
TNFSF12	17p13.1	15	0.34	-0.080 (0.038)	$3.6x10^{-02}$	-0.017 (0.037)	$6.3x10^{-01}$	-0.047 (0.026)	$7.3x10^{-02}$
ZSWIM5	1p34.1	36	0.097	-0.14 (0.057)	$1.2x10^{-02}$	0.004 (0.061)	$9.5x10^{-01}$	-0.075 (0.042)	$7.4x10^{-02}$
PIGU	20q11.22	6	0.0089	0.40 (0.18)	$2.6x10^{-02}$	0.005 (0.24)	$9.8x10^{-01}$	0.25 (0.14)	$7.4x10^{-02}$
PRRC1	5q23.2	14	0.0077	-0.33 (0.15)	$2.9x10^{-02}$	-0.012 (0.21)	$9.6x10^{-01}$	-0.22 (0.12)	$7.4x10^{-02}$
ZNF37A	10p11.1	41	0.091	0.067 (0.033)	$4.5x10^{-02}$	0.007 (0.057)	$9.1x10^{-01}$	0.051 (0.029)	$7.4x10^{-02}$
SERPINB10	18q21.33	69	0.36	-0.060 (0.025)	$1.5x10^{-02}$	0.009 (0.033)	$7.8x10^{-01}$	-0.035 (0.020)	$7.5x10^{-02}$
CCDC28A	6q24.1	62	0.097	-0.14 (0.047)	$3.0x10^{-03}$	0.063 (0.062)	$3.1x10^{-01}$	-0.066 (0.037)	$7.6x10^{-02}$
FAM18B2	17p12	54	0.11	-0.13 (0.049)	$9.0x10^{-03}$	0.022 (0.059)	$7.1x10^{-01}$	-0.066 (0.038)	$7.7x10^{-02}$
ANKRD33B	5p15.2	21	0.054	-0.19 (0.075)	$1.3x10^{-02}$	0.050 (0.11)	$6.4x10^{-01}$	-0.11 (0.061)	$7.7x10^{-02}$
NOTCH4	6p21.32	64	0.12	0.094 (0.041)	$2.1x10^{-02}$	-0.006 (0.053)	$9.1x10^{-01}$	0.057 (0.032)	$7.7x10^{-02}$
SMARCD3	7q36.1	39	0.027	0.22 (0.10)	$2.6x10^{-02}$	0.015 (0.12)	$9.0x10^{-01}$	0.14 (0.077)	$7.7x10^{-02}$
PPIA	7p13	26	0.073	-0.14 (0.069)	$4.7x10^{-02}$	-0.033 (0.079)	$6.8x10^{-01}$	-0.092 (0.052)	$7.7x10^{-02}$
FAM168A	11q13.4	11	0.07	-0.16 (0.073)	$2.7x10^{-02}$	0.000 (0.095)	1.00E+00	-0.10 (0.058)	$7.8x10^{-02}$
S1PR3	9q22.1	15	0.066	0.17 (0.080)	$2.9x10^{-02}$	0.010 (0.099)	$9.2x10^{-01}$	0.11 (0.062)	$7.9x10^{-02}$

		# SNPs in	Imputation	Discovery U4C Meta-analysis		UK Biobank Replication		Joint Meta-analysis	
Gene	Location[a]	Prediction	Quality[b]	Beta (SE)	*p*-value	Beta (SE)	*p*-value	Beta (SE)	*p*-value
LRRC27	10q26.3	35	0.67	0.034 (0.017)	3.8×10^{-02}	0.002 (0.024)	9.4×10^{-01}	0.024 (0.014)	7.9×10^{-02}
HECTD2	10q23.32	2	0.04	0.13 (0.059)	3.1×10^{-02}	-0.093 (0.14)	5.2×10^{-01}	0.095 (0.054)	8.0×10^{-02}
TALDO1	11p15.5	6	0.013	-0.33 (0.13)	1.5×10^{-02}	0.16 (0.23)	4.9×10^{-01}	-0.20 (0.12)	8.1×10^{-02}
SPDYE1	7p13	29	0.016	0.24 (0.100)	1.7×10^{-02}	-0.031 (0.13)	8.1×10^{-01}	0.14 (0.079)	8.1×10^{-02}
RPL10A	6p21.31	26	0.17	0.086 (0.042)	3.9×10^{-02}	0.014 (0.049)	7.8×10^{-01}	0.056 (0.032)	8.1×10^{-02}
CYLD	16q12.1	23	0.079	-0.12 (0.059)	4.2×10^{-02}	-0.012 (0.081)	8.8×10^{-01}	-0.083 (0.048)	8.3×10^{-02}
MED15	22q11.21	27	0.048	0.22 (0.090)	1.5×10^{-02}	-0.018 (0.10)	8.7×10^{-01}	0.12 (0.068)	8.4×10^{-02}
CPSF2	14q32.12	32	0.32	-0.055 (0.025)	2.4×10^{-02}	0.013 (0.038)	7.4×10^{-01}	-0.035 (0.021)	8.5×10^{-02}
SLC20A1	2q13	22	0.3	0.068 (0.034)	4.6×10^{-02}	0.012 (0.040)	7.7×10^{-01}	0.045 (0.026)	8.6×10^{-02}
ALKBH2	12q24.11	9	0.038	-0.21 (0.080)	9.8×10^{-03}	0.12 (0.13)	3.5×10^{-01}	-0.12 (0.068)	8.8×10^{-02}
TTC28	22q12.1	24	0.11	0.12 (0.059)	3.9×10^{-02}	0.009 (0.077)	9.1×10^{-01}	0.080 (0.047)	8.8×10^{-02}
KHDRBS2	6q11.1	16	0.034	-0.21 (0.11)	4.8×10^{-02}	-0.042 (0.12)	7.3×10^{-01}	-0.14 (0.080)	8.8×10^{-02}
UBE2E3	2q31.3	76	0.22	0.098 (0.035)	4.6×10^{-03}	-0.027 (0.040)	5.0×10^{-01}	0.045 (0.026)	8.9×10^{-02}
NDUFC1	4q31.1	29	0.1	-0.13 (0.064)	4.4×10^{-02}	-0.024 (0.067)	7.2×10^{-01}	-0.079 (0.046)	9.0×10^{-02}
AP2A1	19q13.33	41	0.0076	-0.30 (0.11)	5.2×10^{-03}	0.14 (0.15)	3.3×10^{-01}	-0.14 (0.086)	9.2×10^{-02}
MYO9B	19p13.11	6	0.014	0.49 (0.19)	9.9×10^{-03}	-0.14 (0.25)	5.6×10^{-01}	0.25 (0.15)	9.2×10^{-02}
TRPV6	7q34	131	0.25	0.048 (0.020)	1.5×10^{-02}	-0.037 (0.037)	3.1×10^{-01}	0.029 (0.017)	9.3×10^{-02}
ZBTB7C	18q21.1	36	0.13	0.11 (0.053)	3.0×10^{-02}	-0.002 (0.066)	9.8×10^{-01}	0.069 (0.041)	9.3×10^{-02}
UBL7	15q24.1	47	0.013	0.15 (0.069)	3.2×10^{-02}	-0.007 (0.093)	9.4×10^{-01}	0.093 (0.055)	9.3×10^{-02}
GGT7	20q11.22	16	0.093	-0.12 (0.055)	3.5×10^{-02}	0.004 (0.075)	9.6×10^{-01}	-0.074 (0.044)	9.3×10^{-02}
SDC3	1p35.2	28	0.23	-0.061 (0.030)	4.4×10^{-02}	-0.001 (0.045)	9.8×10^{-01}	-0.042 (0.025)	9.3×10^{-02}
CES4A	16q22.1	37	0.011	-0.23 (0.089)	8.3×10^{-03}	0.093 (0.12)	4.4×10^{-01}	-0.12 (0.072)	9.4×10^{-02}
ANP32E	1q21.2	30	0.089	0.14 (0.061)	2.3×10^{-02}	0.000 (0.068)	1.00E+00	0.076 (0.045)	9.4×10^{-02}
ZCCHC7	9p13.2	21	0.09	-0.15 (0.064)	1.6×10^{-02}	0.015 (0.072)	8.4×10^{-01}	-0.080 (0.048)	9.5×10^{-02}
SYNGR2	17q25.3	47	0.11	-0.096 (0.046)	3.7×10^{-02}	0.013 (0.072)	8.6×10^{-01}	-0.065 (0.039)	9.5×10^{-02}
IL6ST	5q11.2	32	0.036	0.27 (0.11)	1.4×10^{-02}	-0.029 (0.12)	8.1×10^{-01}	0.14 (0.082)	9.7×10^{-02}
PTPRM	18p11.23	174	0.33	0.063 (0.027)	2.0×10^{-02}	-0.009 (0.034)	7.9×10^{-01}	0.035 (0.021)	9.7×10^{-02}

Gene	Location[a]	# SNPs in Prediction	Imputation Quality[b]	Discovery U4C Meta-analysis Beta (SE)	Discovery U4C Meta-analysis *p*-value	UK Biobank Replication Beta (SE)	UK Biobank Replication *p*-value	Joint Meta-analysis Beta (SE)	Joint Meta-analysis *p*-value
AFAP1	4p16.1	95	0.72	-0.046 (0.022)	$3.1x10^{-02}$	-0.003 (0.022)	$8.9x10^{-01}$	-0.025 (0.016)	$1.0x10^{-01}$
ANXA5	4q27	66	0.51	-0.057 (0.021)	$6.9x10^{-03}$	0.022 (0.026)	$4.0x10^{-01}$	-0.026 (0.017)	$1.1x10^{-01}$
ZNF167	3p21.31	83	0.14	-0.12 (0.049)	$1.9x10^{-02}$	0.007 (0.050)	$8.9x10^{-01}$	-0.056 (0.035)	$1.1x10^{-01}$
HTRA2	2p13.1	31	0.03	-0.17 (0.074)	$2.1x10^{-02}$	0.049 (0.10)	$6.4x10^{-01}$	-0.097 (0.060)	$1.1x10^{-01}$
TRPV1	17p13.2	52	0.13	0.13 (0.059)	$3.2x10^{-02}$	0.009 (0.055)	$8.7x10^{-01}$	0.065 (0.040)	$1.1x10^{-01}$
COL13A1	10q22.1	21	0.061	-0.18 (0.085)	$3.3x10^{-02}$	0.006 (0.10)	$9.5x10^{-01}$	-0.11 (0.066)	$1.1x10^{-01}$
IPO8	12p11.21	47	0.12	-0.11 (0.052)	$3.5x10^{-02}$	-0.001 (0.060)	$9.9x10^{-01}$	-0.063 (0.039)	$1.1x10^{-01}$
MAP3K1	5q11.2	51	0.051	0.16 (0.080)	$4.1x10^{-02}$	0.009 (0.090)	$9.2x10^{-01}$	0.095 (0.060)	$1.1x10^{-01}$
MTCH1	6p21.2	19	0.022	0.27 (0.13)	$4.2x10^{-02}$	0.022 (0.15)	$8.8x10^{-01}$	0.16 (0.098)	$1.1x10^{-01}$
RPL9	4p14	21	0.83	-0.033 (0.016)	$4.2x10^{-02}$	0.015 (0.032)	$6.3x10^{-01}$	-0.023 (0.014)	$1.1x10^{-01}$
SNCG	10q23.2	19	0.064	0.14 (0.068)	$4.3x10^{-02}$	-0.007 (0.097)	$9.5x10^{-01}$	0.090 (0.056)	$1.1x10^{-01}$
GRK5	10q26.11	86	0.14	-0.076 (0.038)	$4.4x10^{-02}$	0.001 (0.049)	$9.8x10^{-01}$	-0.047 (0.030)	$1.1x10^{-01}$
PCBD2	5q31.1	4	0.059	-0.16 (0.082)	$4.4x10^{-02}$	0.007 (0.11)	$9.5x10^{-01}$	-0.10 (0.066)	$1.1x10^{-01}$
CDH4	20q13.33	50	0.076	-0.14 (0.068)	$4.6x10^{-02}$	-0.007 (0.081)	$9.3x10^{-01}$	-0.082 (0.052)	$1.1x10^{-01}$
C22orf15	22q11.23	14	0.055	-0.14 (0.068)	$4.6x10^{-02}$	0.011 (0.10)	$9.1x10^{-01}$	-0.090 (0.057)	$1.1x10^{-01}$
TMEM184A	7p22.3	15	0.076	0.17 (0.062)	$4.9x10^{-03}$	-0.093 (0.082)	$2.5x10^{-01}$	0.077 (0.049)	$1.2x10^{-01}$
THOC3	5q35.2	22	0.05	0.19 (0.073)	$9.0x10^{-03}$	-0.13 (0.11)	$2.5x10^{-01}$	0.096 (0.061)	$1.2x10^{-01}$
CELF1	11p11.2	6	0.014	0.34 (0.14)	$1.4x10^{-02}$	-0.11 (0.18)	$5.4x10^{-01}$	0.18 (0.11)	$1.2x10^{-01}$
IRAK2	3p25.3	52	0.012	-0.21 (0.086)	$1.7x10^{-02}$	0.092 (0.13)	$4.7x10^{-01}$	-0.11 (0.071)	$1.2x10^{-01}$
RC3H2	9q33.2	14	0.033	0.19 (0.080)	$1.7x10^{-02}$	-0.15 (0.14)	$2.9x10^{-01}$	0.11 (0.070)	$1.2x10^{-01}$
ZNF197	3p21.31	63	0.24	-0.10 (0.042)	$1.8x10^{-02}$	0.005 (0.041)	$9.0x10^{-01}$	-0.046 (0.030)	$1.2x10^{-01}$
PWWP2A	5q33.3	28	0.099	0.12 (0.051)	$2.0x10^{-02}$	-0.029 (0.065)	$6.6x10^{-01}$	0.063 (0.040)	$1.2x10^{-01}$
PREPL	2p21	11	0.099	-0.13 (0.056)	$2.1x10^{-02}$	0.040 (0.075)	$5.9x10^{-01}$	-0.069 (0.045)	$1.2x10^{-01}$
ZNF714	19p12	5	0.0072	-0.37 (0.16)	$2.4x10^{-02}$	0.19 (0.26)	$4.6x10^{-01}$	-0.21 (0.14)	$1.2x10^{-01}$
ZC3H12D	6q25.1	23	0.077	-0.12 (0.054)	$3.2x10^{-02}$	0.039 (0.084)	$6.4x10^{-01}$	-0.071 (0.046)	$1.2x10^{-01}$
KRT72	12q13.13	55	0.57	-0.044 (0.022)	$4.2x10^{-02}$	0.001 (0.027)	$9.7x10^{-01}$	-0.027 (0.017)	$1.2x10^{-01}$
PIK3C2A	11p15.1	7	0.0079	0.57 (0.29)	$4.5x10^{-02}$	-0.009 (0.36)	$9.8x10^{-01}$	0.35 (0.22)	$1.2x10^{-01}$

Gene	Location[a]	# SNPs in Prediction	Imputation Quality[b]	Discovery U4C Meta-analysis Beta (SE)	Discovery U4C Meta-analysis *p*-value	UK Biobank Replication Beta (SE)	UK Biobank Replication *p*-value	Joint Meta-analysis Beta (SE)	Joint Meta-analysis *p*-value
GART	21q22.11	1	0.0091	0.44 (0.22)	$4.7x10^{-02}$	-0.026 (0.30)	$9.3x10^{-01}$	0.28 (0.18)	$1.2x10^{-01}$
ESAM	11q24.2	6	0.0052	0.51 (0.26)	$4.7x10^{-02}$	-0.033 (0.36)	$9.3x10^{-01}$	0.32 (0.21)	$1.2x10^{-01}$
PTK2	8q24.3	14	0.037	-0.25 (0.095)	$9.5x10^{-03}$	0.10 (0.12)	$3.9x10^{-01}$	-0.11 (0.075)	$1.3x10^{-01}$
C17orf62	17q25.3	25	0.1	-0.11 (0.044)	$1.3x10^{-02}$	0.058 (0.063)	$3.6x10^{-01}$	-0.054 (0.036)	$1.3x10^{-01}$
LATS2	13q12.11	50	0.1	-0.14 (0.058)	$1.5x10^{-02}$	0.025 (0.063)	$7.0x10^{-01}$	-0.065 (0.043)	$1.3x10^{-01}$
FRMD4A	10p13	55	0.3	-0.077 (0.032)	$1.7x10^{-02}$	0.018 (0.037)	$6.3x10^{-01}$	-0.037 (0.024)	$1.3x10^{-01}$
SMAD5	5q31.1	25	0.31	0.067 (0.028)	$1.8x10^{-02}$	-0.022 (0.037)	$5.6x10^{-01}$	0.034 (0.023)	$1.3x10^{-01}$
C10orf116	10q23.2	33	0.086	0.16 (0.070)	$2.6x10^{-02}$	-0.013 (0.077)	$8.6x10^{-01}$	0.079 (0.052)	$1.3x10^{-01}$
CLCN2	3q27.1	32	0.033	0.20 (0.097)	$4.1x10^{-02}$	0.003 (0.11)	$9.8x10^{-01}$	0.11 (0.072)	$1.3x10^{-01}$
DPYSL4	10q26.3	43	0.53	-0.056 (0.028)	$4.3x10^{-02}$	-0.003 (0.028)	$9.1x10^{-01}$	-0.030 (0.020)	$1.3x10^{-01}$
C1GALT1	7p22.1	31	0.31	0.071 (0.036)	$4.7x10^{-02}$	0.003 (0.038)	$9.5x10^{-01}$	0.039 (0.026)	$1.3x10^{-01}$
PLEKHA1	10q26.13	29	0.15	0.14 (0.050)	$4.2x10^{-03}$	-0.070 (0.061)	$2.5x10^{-01}$	0.057 (0.039)	$1.4x10^{-01}$
FAM20A	17q24.2	18	0.059	0.20 (0.071)	$5.1x10^{-03}$	-0.11 (0.091)	$2.2x10^{-01}$	0.081 (0.056)	$1.4x10^{-01}$
ITPRIP	10q25.1	40	0.092	-0.14 (0.054)	$9.8x10^{-03}$	0.041 (0.061)	$5.0x10^{-01}$	-0.061 (0.041)	$1.4x10^{-01}$
CCDC93	2q14.1	36	0.012	-0.29 (0.12)	$1.4x10^{-02}$	0.079 (0.14)	$5.7x10^{-01}$	-0.13 (0.090)	$1.4x10^{-01}$
USP10	16q24.1	77	0.069	-0.16 (0.067)	$1.5x10^{-02}$	0.035 (0.074)	$6.4x10^{-01}$	-0.074 (0.050)	$1.4x10^{-01}$
POLR2D	2q14.3	19	0.28	0.093 (0.038)	$1.5x10^{-02}$	-0.019 (0.041)	$6.4x10^{-01}$	0.041 (0.028)	$1.4x10^{-01}$
RFWD2	1q25.2	10	0.016	-0.39 (0.19)	$3.8x10^{-02}$	0.050 (0.23)	$8.3x10^{-01}$	-0.21 (0.14)	$1.4x10^{-01}$
ARF3	12q13.12	29	0.21	-0.063 (0.032)	$4.8x10^{-02}$	0.015 (0.048)	$7.5x10^{-01}$	-0.039 (0.027)	$1.4x10^{-01}$
FPR1	19q13.41	51	0.39	-0.056 (0.028)	$4.9x10^{-02}$	0.005 (0.037)	$9.0x10^{-01}$	-0.034 (0.023)	$1.4x10^{-01}$
ZXDC	3q21.3	26	0.026	0.23 (0.092)	$1.4x10^{-02}$	-0.066 (0.11)	$5.3x10^{-01}$	0.10 (0.070)	$1.5x10^{-01}$
ATXN7L2	1p13.3	21	0.02	-0.28 (0.13)	$2.3x10^{-02}$	0.089 (0.16)	$5.8x10^{-01}$	-0.14 (0.099)	$1.5x10^{-01}$
ZNF487P	10q11.21	2	0.011	-0.60 (0.27)	$2.6x10^{-02}$	0.11 (0.30)	$7.2x10^{-01}$	-0.28 (0.20)	$1.5x10^{-01}$
SEMA7A	15q24.1	2	0.0047	1.80 (0.82)	$3.1x10^{-02}$	-0.38 (1.0)	$7.1x10^{-01}$	0.92 (0.64)	$1.5x10^{-01}$
ZNF563	19p13.2	32	0.07	-0.10 (0.052)	$4.8x10^{-02}$	0.021 (0.073)	$7.8x10^{-01}$	-0.061 (0.042)	$1.5x10^{-01}$
FBXO2	1p36.22	51	0.61	-0.068 (0.023)	$3.7x10^{-03}$	0.028 (0.025)	$2.7x10^{-01}$	-0.024 (0.017)	$1.6x10^{-01}$
TSPAN1	1p34.1	3	0.052	-0.19 (0.084)	$2.0x10^{-02}$	0.085 (0.11)	$4.5x10^{-01}$	-0.095 (0.067)	$1.6x10^{-01}$

Gene	Location[a]	# SNPs in Prediction	Imputation Quality[b]	Discovery U4C Meta-analysis Beta (SE)	Discovery U4C Meta-analysis *p*-value	UK Biobank Replication Beta (SE)	UK Biobank Replication *p*-value	Joint Meta-analysis Beta (SE)	Joint Meta-analysis *p*-value
MNDA	1q23.1	28	0.091	0.12 (0.052)	$2.2x10^{-02}$	-0.048 (0.069)	$4.8x10^{-01}$	0.058 (0.041)	$1.6x10^{-01}$
RPS8	1p34.1	5	0.077	-0.20 (0.089)	$2.4x10^{-02}$	0.012 (0.082)	$8.8x10^{-01}$	-0.085 (0.060)	$1.6x10^{-01}$
PTX4	16p13.3	86	0.028	0.13 (0.058)	$2.8x10^{-02}$	-0.032 (0.071)	$6.5x10^{-01}$	0.064 (0.045)	$1.6x10^{-01}$
AQP11	11q14.1	10	0.017	-0.24 (0.12)	$3.7x10^{-02}$	0.15 (0.20)	$4.5x10^{-01}$	-0.14 (0.099)	$1.6x10^{-01}$
RNF139	8q24.13	49	0.011	-0.18 (0.089)	$3.8x10^{-02}$	0.048 (0.12)	$6.8x10^{-01}$	-0.099 (0.070)	$1.6x10^{-01}$
DCTD	4q35.1	31	0.21	0.091 (0.037)	$1.4x10^{-02}$	-0.056 (0.052)	$2.7x10^{-01}$	0.041 (0.030)	$1.7x10^{-01}$
TMEM8B	9p13.3	22	0.066	0.17 (0.075)	$2.2x10^{-02}$	-0.049 (0.087)	$5.7x10^{-01}$	0.077 (0.057)	$1.7x10^{-01}$
OLFML1	11p15.4	11	0.064	0.17 (0.079)	$2.8x10^{-02}$	-0.067 (0.11)	$5.3x10^{-01}$	0.087 (0.064)	$1.7x10^{-01}$
HK1	10q22.1	31	0.031	0.18 (0.087)	$3.5x10^{-02}$	-0.053 (0.11)	$6.4x10^{-01}$	0.094 (0.069)	$1.7x10^{-01}$
MICA	6p21.33	84	0.67	-0.035 (0.017)	$3.7x10^{-02}$	0.013 (0.023)	$5.8x10^{-01}$	-0.019 (0.014)	$1.7x10^{-01}$
LTN1	21q21.3	9	0.033	-0.27 (0.13)	$3.7x10^{-02}$	0.055 (0.15)	$7.2x10^{-01}$	-0.13 (0.098)	$1.7x10^{-01}$
ALDH6A1	14q24.3	20	0.042	0.15 (0.072)	$3.8x10^{-02}$	-0.055 (0.10)	$5.9x10^{-01}$	0.081 (0.059)	$1.7x10^{-01}$
PTPRA	20p13	28	0.054	-0.19 (0.091)	$3.9x10^{-02}$	0.027 (0.10)	$7.9x10^{-01}$	-0.093 (0.068)	$1.7x10^{-01}$
C1QTNF4	11p11.2	41	0.69	0.044 (0.019)	$2.1x10^{-02}$	-0.014 (0.022)	$5.2x10^{-01}$	0.019 (0.014)	$1.8x10^{-01}$
KATNB1	16q21	47	0.041	-0.15 (0.066)	$2.2x10^{-02}$	0.063 (0.083)	$4.5x10^{-01}$	-0.068 (0.052)	$1.8x10^{-01}$
C7orf53	7q31.1	101	0.12	-0.075 (0.035)	$3.3x10^{-02}$	0.026 (0.046)	$5.7x10^{-01}$	-0.038 (0.028)	$1.8x10^{-01}$
PPM1M	3p21.2	32	0.17	0.084 (0.039)	$3.4x10^{-02}$	-0.022 (0.048)	$6.5x10^{-01}$	0.041 (0.031)	$1.8x10^{-01}$
DUSP18	22q12.2	58	0.32	0.059 (0.029)	$4.2x10^{-02}$	-0.011 (0.035)	$7.5x10^{-01}$	0.030 (0.022)	$1.8x10^{-01}$
FAM83A	8q24.13	17	0.3	0.063 (0.031)	$4.2x10^{-02}$	-0.017 (0.040)	$6.7x10^{-01}$	0.033 (0.024)	$1.8x10^{-01}$
FOXK2	17q25.3	23	0.057	0.15 (0.059)	$1.1x10^{-02}$	-0.13 (0.088)	$1.4x10^{-01}$	0.063 (0.049)	$1.9x10^{-01}$
RNMT	18p11.21	24	0.16	-0.11 (0.048)	$1.6x10^{-02}$	0.032 (0.050)	$5.3x10^{-01}$	-0.046 (0.035)	$1.9x10^{-01}$
PHLDA1	12q21.2	34	0.025	0.22 (0.095)	$2.1x10^{-02}$	-0.16 (0.14)	$2.7x10^{-01}$	0.10 (0.079)	$1.9x10^{-01}$
TCP11L2	12q23.3	31	0.075	0.14 (0.061)	$2.4x10^{-02}$	-0.046 (0.072)	$5.3x10^{-01}$	0.061 (0.047)	$1.9x10^{-01}$
GSTK1	7q34	16	0.15	0.081 (0.039)	$3.7x10^{-02}$	-0.044 (0.058)	$4.5x10^{-01}$	0.042 (0.032)	$1.9x10^{-01}$
MKS1	17q22	27	0.0076	-0.34 (0.11)	$1.8x10^{-03}$	0.31 (0.15)	$3.5x10^{-02}$	-0.11 (0.087)	$2.0x10^{-01}$
ARHGAP32	11q24.3	31	0.1	0.17 (0.059)	$4.0x10^{-03}$	-0.12 (0.074)	$1.2x10^{-01}$	0.059 (0.046)	$2.0x10^{-01}$
ADSL	22q13.1	9	0.025	0.27 (0.11)	$1.4x10^{-02}$	-0.11 (0.13)	$3.8x10^{-01}$	0.11 (0.084)	$2.0x10^{-01}$

Gene	Location[a]	# SNPs in Prediction	Imputation Quality[b]	Discovery U4C Meta-analysis Beta (SE)	*p*-value	UK Biobank Replication Beta (SE)	*p*-value	Joint Meta-analysis Beta (SE)	*p*-value
ZNF438	10p11.22	17	0.02	-0.30 (0.13)	1.9×10^{-02}	0.12 (0.15)	4.4×10^{-01}	-0.13 (0.098)	2.0×10^{-01}
FAM96A	15q22.31	14	0.005	0.42 (0.18)	1.9×10^{-02}	-0.29 (0.26)	2.6×10^{-01}	0.19 (0.15)	2.0×10^{-01}
TRNT1	3p26.2	26	0.31	-0.063 (0.028)	2.3×10^{-02}	0.036 (0.038)	3.4×10^{-01}	-0.029 (0.022)	2.0×10^{-01}
TTC9C	11q12.3	30	0.25	-0.069 (0.032)	2.9×10^{-02}	0.030 (0.041)	4.6×10^{-01}	-0.033 (0.025)	2.0×10^{-01}
SND1	7q32.1	13	0.045	0.31 (0.12)	1.1×10^{-02}	-0.098 (0.12)	4.2×10^{-01}	0.11 (0.086)	2.1×10^{-01}
ZNF76	6p21.31	23	0.12	-0.095 (0.043)	2.6×10^{-02}	0.073 (0.065)	2.7×10^{-01}	-0.045 (0.036)	2.1×10^{-01}
FUT8	14q23.3	26	0.19	0.091 (0.044)	3.8×10^{-02}	-0.031 (0.054)	5.7×10^{-01}	0.043 (0.034)	2.1×10^{-01}
C5orf20	5q31.1	29	0.043	-0.17 (0.080)	3.9×10^{-02}	0.057 (0.10)	5.7×10^{-01}	-0.079 (0.063)	2.1×10^{-01}
ISCU	12q23.3	72	0.29	0.062 (0.031)	4.5×10^{-02}	-0.018 (0.038)	6.4×10^{-01}	0.030 (0.024)	2.1×10^{-01}
ZNF668	16p11.2	22	0.26	0.10 (0.039)	8.8×10^{-03}	-0.036 (0.040)	3.6×10^{-01}	0.034 (0.028)	2.2×10^{-01}
CLPB	11q13.4	29	0.0067	0.25 (0.098)	1.3×10^{-02}	-0.28 (0.16)	8.2×10^{-02}	0.10 (0.084)	2.2×10^{-01}
PROCR	20q11.22	38	0.041	0.17 (0.075)	2.5×10^{-02}	-0.045 (0.079)	5.7×10^{-01}	0.067 (0.054)	2.2×10^{-01}
DHX37	12q24.31	31	0.014	0.29 (0.13)	3.1×10^{-02}	-0.11 (0.16)	4.9×10^{-01}	0.13 (0.10)	2.2×10^{-01}
AKAP3	12p13.32	11	0.3	0.061 (0.031)	4.9×10^{-02}	-0.020 (0.039)	6.2×10^{-01}	0.030 (0.024)	2.2×10^{-01}
MCM3	6p12.2	43	0.024	-0.30 (0.11)	7.8×10^{-03}	0.13 (0.12)	2.7×10^{-01}	-0.097 (0.081)	2.3×10^{-01}
PDHB	3p14.3	44	0.24	-0.075 (0.030)	1.1×10^{-02}	0.062 (0.041)	1.4×10^{-01}	-0.029 (0.024)	2.3×10^{-01}
CCDC127	5p15.33	15	0.33	-0.072 (0.030)	1.8×10^{-02}	0.032 (0.036)	3.6×10^{-01}	-0.028 (0.023)	2.3×10^{-01}
PCYOX1L	5q32	25	0.39	0.058 (0.025)	2.3×10^{-02}	-0.033 (0.033)	3.2×10^{-01}	0.024 (0.020)	2.3×10^{-01}
PTER	10p13	45	0.28	0.073 (0.035)	3.6×10^{-02}	-0.027 (0.042)	5.1×10^{-01}	0.032 (0.027)	2.3×10^{-01}
FAM26F	6q22.1	104	0.49	-0.057 (0.024)	1.6×10^{-02}	0.028 (0.028)	3.2×10^{-01}	-0.021 (0.018)	2.4×10^{-01}
PPP1R3B	8p23.1	12	0.0087	0.43 (0.18)	1.7×10^{-02}	-0.22 (0.22)	3.1×10^{-01}	0.16 (0.14)	2.4×10^{-01}
PPM1K	4q22.1	23	0.088	0.14 (0.065)	3.3×10^{-02}	-0.043 (0.072)	5.5×10^{-01}	0.056 (0.048)	2.4×10^{-01}
HAGHL	16p13.3	37	0.23	-0.074 (0.035)	3.7×10^{-02}	0.031 (0.044)	4.8×10^{-01}	-0.032 (0.027)	2.4×10^{-01}
ACACB	12q24.11	29	0.28	-0.060 (0.030)	4.7×10^{-02}	0.024 (0.038)	5.3×10^{-01}	-0.028 (0.024)	2.4×10^{-01}
ADSSL1	14q32.33	10	0.015	-0.32 (0.16)	4.9×10^{-02}	0.095 (0.19)	6.1×10^{-01}	-0.14 (0.12)	2.4×10^{-01}
NAA20	20p11.23	27	0.014	-0.27 (0.11)	2.0×10^{-02}	0.23 (0.17)	1.7×10^{-01}	-0.11 (0.094)	2.5×10^{-01}
ZNF202	11q24.1	15	0.054	0.20 (0.092)	2.9×10^{-02}	-0.11 (0.12)	3.6×10^{-01}	0.082 (0.072)	2.5×10^{-01}

Gene	Location[a]	# SNPs in Prediction	Imputation Quality[b]	Discovery U4C Meta-analysis Beta (SE)	Discovery U4C Meta-analysis *p*-value	UK Biobank Replication Beta (SE)	UK Biobank Replication *p*-value	Joint Meta-analysis Beta (SE)	Joint Meta-analysis *p*-value
RPF2	6q21	8	0.24	-0.064 (0.031)	4.0×10^{-02}	0.038 (0.044)	3.8×10^{-01}	-0.030 (0.025)	2.5×10^{-01}
CABIN1	22q11.23	18	0.0073	0.25 (0.13)	4.7×10^{-02}	-0.25 (0.22)	2.6×10^{-01}	0.13 (0.11)	2.5×10^{-01}
NUCB2	11p15.1	30	0.11	0.14 (0.054)	7.9×10^{-03}	-0.087 (0.063)	1.7×10^{-01}	0.047 (0.041)	2.6×10^{-01}
C6orf57	6q13	11	0.019	0.36 (0.14)	8.7×10^{-03}	-0.22 (0.16)	1.8×10^{-01}	0.12 (0.11)	2.6×10^{-01}
ZNF502	3p21.31	46	0.6	-0.071 (0.027)	9.1×10^{-03}	0.024 (0.026)	3.5×10^{-01}	-0.021 (0.019)	2.6×10^{-01}
C10orf10	10q11.21	31	0.035	0.14 (0.069)	3.9×10^{-02}	-0.12 (0.11)	2.8×10^{-01}	0.066 (0.058)	2.6×10^{-01}
SYNGR3	16p13.3	10	0.36	0.10 (0.038)	7.8×10^{-03}	-0.032 (0.035)	3.6×10^{-01}	0.029 (0.026)	2.7×10^{-01}
MAPK8IP2	22q13.33	13	0.04	-0.22 (0.086)	1.2×10^{-02}	0.18 (0.12)	1.2×10^{-01}	-0.075 (0.069)	2.7×10^{-01}
DYNLRB1	20q11.22	40	0.0048	0.21 (0.087)	1.5×10^{-02}	-0.25 (0.14)	7.1×10^{-02}	0.082 (0.074)	2.7×10^{-01}
CHMP4B	20q11.22	27	0.18	0.095 (0.042)	2.4×10^{-02}	-0.049 (0.050)	3.3×10^{-01}	0.036 (0.032)	2.7×10^{-01}
ZCCHC24	10q22.3	16	0.044	-0.19 (0.084)	2.5×10^{-02}	0.11 (0.11)	2.8×10^{-01}	-0.072 (0.066)	2.7×10^{-01}
HLA-C	6p21.33	104	0.67	-0.048 (0.022)	2.7×10^{-02}	0.021 (0.024)	3.9×10^{-01}	-0.018 (0.016)	2.8×10^{-01}
VANGL1	1p13.1	68	0.16	-0.074 (0.036)	4.1×10^{-02}	0.042 (0.047)	3.8×10^{-01}	-0.031 (0.029)	2.8×10^{-01}
MAL	2q11.1	16	0.025	0.26 (0.11)	1.5×10^{-02}	-0.19 (0.13)	1.6×10^{-01}	0.087 (0.083)	2.9×10^{-01}
PROM2	2q11.1	7	0.0094	-0.40 (0.16)	1.6×10^{-02}	0.29 (0.21)	1.7×10^{-01}	-0.14 (0.13)	2.9×10^{-01}
DNMT3A	2p23.3	18	0.085	-0.13 (0.054)	1.8×10^{-02}	0.11 (0.074)	1.5×10^{-01}	-0.046 (0.044)	2.9×10^{-01}
SMC6	2p24.2	15	0.01	0.28 (0.14)	3.9×10^{-02}	-0.16 (0.17)	3.6×10^{-01}	0.11 (0.11)	2.9×10^{-01}
RPP14	3p14.3	27	0.11	-0.18 (0.062)	4.6×10^{-03}	0.088 (0.063)	1.6×10^{-01}	-0.046 (0.044)	3.0×10^{-01}
CALML6	1p36.33	27	0.1	0.12 (0.056)	3.1×10^{-02}	-0.064 (0.067)	3.4×10^{-01}	0.045 (0.043)	3.0×10^{-01}
SNx10	7p15.2	3	0.006	0.78 (0.36)	3.2×10^{-02}	-0.43 (0.44)	3.3×10^{-01}	0.29 (0.28)	3.0×10^{-01}
PARN	16p13.12	4	0.014	-0.42 (0.20)	3.4×10^{-02}	0.44 (0.31)	1.6×10^{-01}	-0.17 (0.17)	3.0×10^{-01}
STAC	3p22.3	66	0.087	0.11 (0.051)	3.8×10^{-02}	-0.059 (0.064)	3.5×10^{-01}	0.042 (0.040)	3.0×10^{-01}
C16orf79	16p13.3	39	0.11	-0.11 (0.047)	2.4×10^{-02}	0.071 (0.059)	2.3×10^{-01}	-0.038 (0.037)	3.1×10^{-01}
GATSL3	22q12.2	17	0.26	-0.069 (0.032)	2.9×10^{-02}	0.050 (0.042)	2.3×10^{-01}	-0.026 (0.025)	3.1×10^{-01}
MYBPC2	19q13.33	20	0.0093	0.25 (0.12)	3.9×10^{-02}	-0.22 (0.18)	2.2×10^{-01}	0.10 (0.10)	3.1×10^{-01}
JHDM1D	7q34	18	0.032	0.24 (0.11)	2.1×10^{-02}	-0.19 (0.14)	1.6×10^{-01}	0.083 (0.084)	3.2×10^{-01}
RBBP6	16p12.1	59	0.37	-0.074 (0.032)	2.3×10^{-02}	0.028 (0.032)	3.8×10^{-01}	-0.022 (0.023)	3.3×10^{-01}

Gene	Location[a]	# SNPs in Prediction	Imputation Quality[b]	Discovery U4C Meta-analysis Beta (SE)	Discovery U4C Meta-analysis *p*-value	UK Biobank Replication Beta (SE)	UK Biobank Replication *p*-value	Joint Meta-analysis Beta (SE)	Joint Meta-analysis *p*-value
MIA	19q13.2	9	0.0048	-0.44 (0.22)	4.5×10^{-02}	0.56 (0.37)	1.3×10^{-01}	-0.18 (0.19)	3.3×10^{-01}
RGPD4	2q12.3	21	0.67	-0.051 (0.021)	1.8×10^{-02}	0.029 (0.024)	2.2×10^{-01}	-0.015 (0.016)	3.4×10^{-01}
TCERG1	5q32	19	0.016	0.31 (0.14)	2.5×10^{-02}	-0.13 (0.14)	3.6×10^{-01}	0.093 (0.098)	3.4×10^{-01}
FBXO44	1p36.22	39	0.31	-0.083 (0.035)	1.9×10^{-02}	0.049 (0.040)	2.2×10^{-01}	-0.025 (0.026)	3.5×10^{-01}
SIPA1	11q13.1	21	0.037	0.17 (0.079)	2.9×10^{-02}	-0.15 (0.11)	1.7×10^{-01}	0.059 (0.064)	3.5×10^{-01}
STX4	16p11.2	14	0.12	-0.12 (0.057)	3.1×10^{-02}	0.057 (0.061)	3.5×10^{-01}	-0.039 (0.042)	3.5×10^{-01}
SCP2	1p32.3	44	0.15	0.093 (0.043)	3.2×10^{-02}	-0.083 (0.060)	1.6×10^{-01}	0.032 (0.035)	3.6×10^{-01}
SNX1	15q22.31	17	0.19	-0.099 (0.044)	2.6×10^{-02}	0.064 (0.052)	2.2×10^{-01}	-0.030 (0.034)	3.7×10^{-01}
ZSWIM1	20q13.12	17	0.0082	-0.34 (0.14)	1.3×10^{-02}	0.44 (0.20)	3.1×10^{-02}	-0.097 (0.11)	4.0×10^{-01}
UBR2	6p21.1	3	0.023	0.45 (0.23)	4.5×10^{-02}	-0.14 (0.20)	5.0×10^{-01}	0.13 (0.15)	4.0×10^{-01}
HIST1H3D	6p22.2	15	0.02	0.28 (0.14)	4.5×10^{-02}	-0.18 (0.16)	2.8×10^{-01}	0.087 (0.11)	4.2×10^{-01}
ZNF805	19q13.43	19	0.059	-0.18 (0.080)	2.8×10^{-02}	0.14 (0.098)	1.5×10^{-01}	-0.049 (0.062)	4.3×10^{-01}
DNAH6	2p11.2	61	0.15	-0.098 (0.045)	3.1×10^{-02}	0.076 (0.055)	1.7×10^{-01}	-0.028 (0.035)	4.3×10^{-01}
STIM2	4p15.2	51	0.1	0.092 (0.045)	4.0×10^{-02}	-0.079 (0.057)	1.6×10^{-01}	0.026 (0.035)	4.5×10^{-01}
HIST1H4D	6p22.2	4	0.0094	0.50 (0.23)	2.7×10^{-02}	-0.49 (0.30)	1.0×10^{-01}	0.13 (0.18)	4.6×10^{-01}
BCKDK	16p11.2	16	0.081	0.16 (0.074)	3.1×10^{-02}	-0.082 (0.074)	2.7×10^{-01}	0.038 (0.053)	4.6×10^{-01}
TTC30A	2q31.2	26	0.015	0.27 (0.14)	4.3×10^{-02}	-0.24 (0.17)	1.7×10^{-01}	0.079 (0.11)	4.6×10^{-01}
TMEM206	1q32.3	21	0.096	0.15 (0.061)	1.6×10^{-02}	-0.14 (0.075)	6.7×10^{-02}	0.034 (0.047)	4.7×10^{-01}
PGP	16p13.3	6	0.099	-0.13 (0.063)	3.3×10^{-02}	0.12 (0.080)	1.3×10^{-01}	-0.036 (0.050)	4.7×10^{-01}
SETD1A	16p11.2	15	0.016	-0.38 (0.16)	1.4×10^{-02}	0.26 (0.17)	1.2×10^{-01}	-0.079 (0.11)	4.8×10^{-01}
ARHGAP24	4q21.23	66	0.23	0.078 (0.036)	3.2×10^{-02}	-0.059 (0.042)	1.6×10^{-01}	0.019 (0.027)	4.8×10^{-01}
STAM	10p12.33	3	0.023	-0.37 (0.18)	3.8×10^{-02}	0.29 (0.21)	1.7×10^{-01}	-0.095 (0.14)	4.8×10^{-01}
KSR1	17q11.1	29	0.24	0.060 (0.030)	4.7×10^{-02}	-0.062 (0.041)	1.2×10^{-01}	0.017 (0.024)	4.9×10^{-01}
EGLN3	14q13.1	66	0.11	-0.085 (0.044)	5.0×10^{-02}	0.083 (0.056)	1.4×10^{-01}	-0.022 (0.034)	5.2×10^{-01}
GDF11	12q13.2	40	0.033	0.18 (0.074)	1.7×10^{-02}	-0.16 (0.087)	6.4×10^{-02}	0.035 (0.056)	5.4×10^{-01}
RPS2	16p13.3	12	0.31	-0.078 (0.038)	3.8×10^{-02}	0.049 (0.039)	2.1×10^{-01}	-0.016 (0.027)	5.4×10^{-01}
KCNS1	20q13.12	18	0.044	-0.24 (0.12)	4.2×10^{-02}	0.14 (0.12)	2.5×10^{-01}	-0.051 (0.083)	5.4×10^{-01}

Gene	Location[a]	# SNPs in Prediction	Imputation Quality[b]	Discovery U4C Meta-analysis Beta (SE)	Discovery U4C Meta-analysis *p*-value	UK Biobank Replication Beta (SE)	UK Biobank Replication *p*-value	Joint Meta-analysis Beta (SE)	Joint Meta-analysis *p*-value
GIMAP4	7q36.1	67	0.08	-0.12 (0.061)	$4.6x10^{-02}$	0.097 (0.070)	$1.7x10^{-01}$	-0.028 (0.046)	$5.5x10^{-01}$
MAP3K8	10p11.23	59	0.034	-0.13 (0.065)	$3.9x10^{-02}$	0.15 (0.085)	$8.4x10^{-02}$	-0.031 (0.052)	$5.6x10^{-01}$
ADCY10	1q24.2	39	0.0056	-0.30 (0.15)	$4.5x10^{-02}$	0.30 (0.19)	$1.1x10^{-01}$	-0.068 (0.12)	$5.6x10^{-01}$
PCTP	17q22	40	0.23	-0.078 (0.035)	$2.8x10^{-02}$	0.11 (0.051)	$3.0x10^{-02}$	-0.016 (0.029)	$5.7x10^{-01}$
C3orf23	3p21.31	9	0.053	-0.18 (0.089)	$4.2x10^{-02}$	0.19 (0.11)	$9.6x10^{-02}$	-0.040 (0.070)	$5.7x10^{-01}$
NCF2	1q25.3	67	0.02	-0.13 (0.066)	$5.0x10^{-02}$	0.14 (0.086)	$1.1x10^{-01}$	-0.030 (0.053)	$5.7x10^{-01}$
MCMBP	10q26.11	77	0.19	0.093 (0.043)	$3.0x10^{-02}$	-0.063 (0.044)	$1.5x10^{-01}$	0.017 (0.031)	$5.8x10^{-01}$
EBF4	20p13	48	0.35	-0.072 (0.029)	$1.4x10^{-02}$	0.088 (0.038)	$2.0x10^{-02}$	-0.012 (0.023)	$6.0x10^{-01}$
FAM83H	8q24.3	15	0.046	-0.17 (0.081)	$4.0x10^{-02}$	0.18 (0.10)	$7.4x10^{-02}$	-0.033 (0.063)	$6.0x10^{-01}$
TPM2	9p13.3	23	0.54	0.051 (0.025)	$4.0x10^{-02}$	-0.042 (0.027)	$1.2x10^{-01}$	0.009 (0.018)	$6.3x10^{-01}$
NAV2	11p15.1	10	0.035	-0.28 (0.12)	$1.9x10^{-02}$	0.34 (0.15)	$2.5x10^{-02}$	-0.041 (0.093)	$6.6x10^{-01}$
PFN2	3q25.1	46	0.39	-0.059 (0.029)	$4.0x10^{-02}$	0.056 (0.033)	$8.6x10^{-02}$	-0.009 (0.022)	$6.8x10^{-01}$
AURKC	19q13.43	48	0.086	0.11 (0.053)	$4.6x10^{-02}$	-0.13 (0.067)	$6.2x10^{-02}$	0.017 (0.042)	$6.9x10^{-01}$
ZNF425	7q36.1	27	0.28	0.069 (0.033)	$3.6x10^{-02}$	-0.076 (0.039)	$5.4x10^{-02}$	0.010 (0.025)	$7.0x10^{-01}$
SPINT2	19q13.2	39	0.091	0.099 (0.050)	$4.5x10^{-02}$	-0.13 (0.065)	$4.1x10^{-02}$	0.014 (0.039)	$7.3x10^{-01}$
SCGB3A2	5q32	48	0.53	-0.047 (0.024)	$4.6x10^{-02}$	0.083 (0.028)	$3.3x10^{-03}$	0.006 (0.018)	$7.3x10^{-01}$
HLA-B	6p21.33	84	0.54	0.041 (0.021)	$4.6x10^{-02}$	-0.049 (0.025)	$5.3x10^{-02}$	0.005 (0.016)	$7.5x10^{-01}$
NUB1	7q36.1	18	0.048	-0.20 (0.097)	$4.1x10^{-02}$	0.22 (0.11)	$5.1x10^{-02}$	-0.021 (0.074)	$7.8x10^{-01}$
DACT3	19q13.32	16	0.12	-0.11 (0.053)	$4.0x10^{-02}$	0.13 (0.062)	$4.4x10^{-02}$	-0.011 (0.040)	$7.9x10^{-01}$
TMEM98	17q11.2	56	0.51	-0.058 (0.028)	$3.7x10^{-02}$	0.047 (0.027)	$7.9x10^{-02}$	-0.004 (0.019)	$8.5x10^{-01}$
FCF1	14q24.3	15	0.01	0.44 (0.18)	$1.8x10^{-02}$	-0.71 (0.24)	$3.4x10^{-03}$	0.017 (0.15)	$9.1x10^{-01}$
SACM1L	3p21.31	40	0.21	0.086 (0.043)	$4.5x10^{-02}$	-0.11 (0.049)	$3.1x10^{-02}$	0.002 (0.032)	$9.4x10^{-01}$
RPS6KL1	14q24.3	13	0.29	0.087 (0.033)	$8.4x10^{-03}$	-0.12 (0.039)	$2.4x10^{-03}$	0.001 (0.025)	$9.6x10^{-01}$

Abbreviations: SE: standard error; SNP: single nucleotide polymorphism; U4C: Up for a Challenge
[a] According to human reference genome GRCh37/hg19
[b] R^2 estimate derived from 10-fold cross-validation of true gene expression and predicted gene expression
