# Supplementary material for: Cis-eQTL-based trans-ethnic meta-analysis reveals novel genes associated with breast cancer risk
Source: PLoS Genet. 2017 Mar 31;13(3):e1006690. doi: 10.1371/journal.pgen.1006690 (PMC5391966; doi:10.1371/journal.pgen.1006690)
Supplement: S2 Table — Association of breast cancer risk with SNPs that contribute to the expression of (A) RCCD1 in breast tissue, (B) DHODH in breast tissue, (C) ANKLE1 in breast tissue, (D) RCCD1 in whole blood, (E) ACAP1 in whole blood, and (F) LRRC25 in whole blood. (PDF) [file pgen.1006690.s002.pdf]

(A) *RCCD1* at 15q26.1 (Breast Tissue)

SNP	Alleles[a]	Proportion of Weight[b]	Discovery U4C Meta-analysis			UK Biobank Replication			Joint Meta-analysis	
			EAF[c]	OR (95% CI)	*p*-value	EAF[c]	OR (95% CI)	*p*-value	OR (95% CI)	*p*-value
rs3826033	G / A	0.13	0.32	0.92 (0.88, 0.98)	4.1×10^{-03}	0.13	0.86 (0.79,0.93)	2.3×10^{-04}	0.90 (0.86,0.94)	9.5×10^{-06}
rs2290202	G / T	0.24	0.3	0.93 (0.89, 0.98)	5.3×10^{-03}	0.13	0.86 (0.79,0.93)	1.9×10^{-04}	0.91 (0.88,0.95)	1.7×10^{-05}
rs4347602	A / C	0.025	0.72	0.94 (0.90,0.98)	6.5×10^{-03}	0.77	0.96 (0.90,1.02)	1.6×10^{-01}	0.94 (0.91,0.98)	2.4×10^{-03}
rs11207	C / T	0.030	0.35	0.97 (0.93, 1.02)	2.1×10^{-01}	0.24	0.93 (0.87,0.98)	1.5×10^{-02}	0.96 (0.93,0.99)	1.6×10^{-02}
rs12907681	C / T	0.0045	0.15	0.96 (0.90, 1.02)	1.6×10^{-01}	0.03	0.88 (0.75,1.03)	1.2×10^{-01}	0.94 (0.89,1.00)	5.8×10^{-02}
rs756874	A / G	0.034	0.36	0.98 (0.93,1.03)	4.6×10^{-01}	0.35	0.96 (0.90,1.01)	1.1×10^{-01}	0.97 (0.93,1.01)	1.1×10^{-01}
rs17595461	A / G	0.0065	0.40	0.98 (0.93,1.02)	2.5×10^{-01}	0.35	0.97 (0.92,1.03)	3.2×10^{-01}	0.97 (0.94,1.01)	1.3×10^{-01}
rs2444061	T / G	0.0078	0.44	1.04 (1.00,1.09)	7.1×10^{-02}	0.13	0.99 (0.92,1.07)	7.8×10^{-01}	1.03 (0.99,1.06)	1.4×10^{-01}
rs2601166	G / A	0.0053	0.43	1.04 (1.00, 1.08)	7.3×10^{-02}	0.13	0.99 (0.91,1.07)	7.6×10^{-01}	1.03 (0.99,1.07)	1.5×10^{-01}
rs7184015	T / G	<0.001	0.48	1.01 (0.97,1.06)	5.4×10^{-01}	0.74	1.05 (0.99,1.11)	1.2×10^{-01}	1.03 (0.99,1.06)	1.5×10^{-01}
rs12912915	G / A	0.028	0.68	0.98 (0.93, 1.02)	3.4×10^{-01}	0.65	0.97 (0.92,1.03)	2.8×10^{-01}	0.97 (0.94,1.01)	1.5×10^{-01}
rs441399	G / A	0.043	0.52	0.97 (0.93, 1.01)	1.5×10^{-01}	0.52	1.00 (0.95,1.05)	1.0e+00	0.98 (0.95,1.01)	2.6×10^{-01}
rs10444879	T / C	0.038	0.90	0.99 (0.92,1.08)	8.8×10^{-01}	0.86	0.95 (0.88,1.03)	2.2×10^{-01}	0.97 (0.92,1.03)	3.2×10^{-01}
rs7496004	A / G	0.045	0.37	1.02 (0.97,1.06)	4.5×10^{-01}	0.34	1.01 (0.96,1.07)	6.6×10^{-01}	1.01 (0.98,1.05)	3.8×10^{-01}
rs7164810	G / A	0.057	0.084	0.96 (0.86, 1.06)	4.1×10^{-01}	-	-	-	0.96 (0.86,1.06)	4.1×10^{-01}
rs3862439	G / T	0.035	0.055	0.98 (0.86, 1.11)	7.4×10^{-01}	0.07	1.07 (0.96,1.18)	2.1×10^{-01}	1.03 (0.95,1.11)	4.4×10^{-01}
rs4932525	C / T	0.0016	0.74	0.99 (0.94, 1.04)	5.5×10^{-01}	0.59	0.99 (0.94,1.04)	6.8×10^{-01}	0.99 (0.95,1.02)	4.7×10^{-01}
rs17832339	G / T	0.027	0.027	1.04 (0.88, 1.23)	6.6×10^{-01}	0.04	0.92 (0.8,1.06)	2.4×10^{-01}	0.97 (0.87,1.08)	5.4×10^{-01}
rs8032568	T / C	0.041	0.50	1.00 (0.96,1.05)	8.7×10^{-01}	0.43	0.97 (0.92,1.03)	3.5×10^{-01}	0.99 (0.96,1.03)	6.5×10^{-01}
rs11639135	T / C	0.024	0.52	1.03 (0.99,1.06)	2.0×10^{-01}	0.58	0.97 (0.92,1.02)	2.7×10^{-01}	1.01 (0.97,1.04)	7.0×10^{-01}
rs2285625	T / C	0.0061	0.43	1.01 (0.96,1.05)	7.1×10^{-01}	0.30	0.97 (0.92,1.03)	3.3×10^{-01}	0.99 (0.96,1.03)	7.6×10^{-01}
rs12906053	T / C	0.0028	0.89	0.98 (0.92,1.05)	5.7×10^{-01}	0.90	1.05 (0.97,1.15)	2.4×10^{-01}	1.01 (0.95,1.06)	8.0×10^{-01}
rs17821347	A / G	0.16	0.07	1.10 (0.96,1.25)	1.8×10^{-01}	0.04	0.92 (0.8,1.06)	2.3×10^{-01}	1.01 (0.92,1.11)	8.9×10^{-01}
rs7176401	G / A	0.0057	0.52	1.01 (0.97, 1.06)	5.9×10^{-01}	0.46	0.98 (0.93,1.04)	5.9×10^{-01}	1.00 (0.97,1.04)	9.5×10^{-01}

(B) *DHODH* at 16q22.2 (Breast Tissue)

		Proportion	Discovery U4C Meta-analysis			UK Biobank Replication			Joint Meta-analysis	
**SNP**	**Alleles[a]**	**of Weight[b]**	**EAF[c]**	**OR (95% CI)**	***p*-value**	**EAF[c]**	**OR (95% CI)**	***p*-value**	**OR (95% CI)**	***p*-value**
rs3213422	C / A	0.56	0.42	0.92 (0.88,0.96)	$2.8x10^{-05}$	0.48	0.95 (0.90,1.00)	$3.9x10^{-02}$	0.93 (0.90,0.96)	$4.5x10^{-06}$
rs2240243	G / A	0.055	0.47	0.93 (0.89,0.97)	$2.7x10^{-04}$	0.34	0.98 (0.93,1.04)	$5.3x10^{-01}$	0.95 (0.92,0.98)	$1.0x10^{-03}$
rs12708928	C / A	0.019	0.47	0.93 (0.89,0.96)	$2.5x10^{-04}$	0.34	0.99 (0.93,1.04)	$5.9x10^{-01}$	0.95 (0.92,0.98)	$1.2x10^{-03}$
rs11640722	T / C	0.097	0.88	0.93 (0.97,1.01)	$8.9x10^{-02}$	0.84	0.96 (0.89,1.04)	$3.2x10^{-01}$	0.95 (0.90,1.00)	$6.0x10^{-02}$
rs7190257	G / T	0.16	0.89	0.99 (0.92,1.06)	$7.9x10^{-01}$	0.83	1.00 (0.93,1.07)	$8.9x10^{-01}$	0.99 (0.95,1.04)	$7.7x10^{-01}$
rs7404992	C / T	0.046	0.25	1.00 (0.95,1.05)	$9.0x10^{-01}$	0.06	1.04 (0.93,1.16)	$4.9x10^{-01}$	1.00 (0.96,1.05)	$8.6x10^{-01}$
rs9923937	T / C	0.069	0.09	1.03 (0.95,1.10)	$4.7x10^{-01}$	0.05	0.95 (0.84,1.07)	$3.7x10^{-01}$	1.00 (0.94,1.06)	$8.9x10^{-01}$

(C) *ANKLE1* at 19p13.11 (Breast Tissue)

		Proportion	Discovery U4C Meta-analysis			UK Biobank Replication			Joint Meta-analysis	
**SNP**	**Alleles[a]**	**of Weight[b]**	**EAF[c]**	**OR (95% CI)**	***p*-value**	**EAF[c]**	**OR (95% CI)**	***p*-value**	**OR (95% CI)**	***p*-value**
rs34084277	A / G	0.23	0.19	1.09 (1.02,1.15)	7.1x10-^{03}	0.19	1.11 (1.04,1.18)	$2.0x10^{-03}$	1.10 (1.05,1.14)	$4.7x10^{-05}$
rs8170	G / A	0.26	0.19	1.08 (1.02,1.15)	$7.2x10^{-03}$	0.19	1.11 (1.04,1.18)	$2.6x10^{-03}$	1.09 (1.05,1.14)	$6.3x10^{-05}$
rs3745162	T / G	0.24	0.81	1.00 (0.94,1.05)	$9.4x10^{-01}$	0.92	1.12 (1.01,1.24)	$2.9x10^{-02}$	1.02 (0.98,1.08)	$3.2x10^{-01}$
rs10409801	A / G	0.11	0.71	1.01 (0.95,1.06)	$8.5x10^{-01}$	0.86	1.04 (0.96,1.12)	$3.3x10^{-01}$	1.02 (0.97,1.06)	$4.8x10^{-01}$
rs2450831	T / C	0.15	0.45	0.99 (0.94,1.04)	$6.8x10^{-01}$	0.17	1.05 (0.98,1.13)	$1.7x10^{-01}$	1.01 (0.97,1.05)	$6.5x10^{-01}$
rs12983375	C / T	0.017	0.42	1.00 (0.96,1.04)	$9.4x10^{-01}$	0.38	0.99 (0.94,1.04)	$7.3x10^{-01}$	1.00 (0.97,1.03)	$8.9x10^{-01}$

(D) *RCCD1* at 15q26.1 (Whole Blood)

SNP	Alleles[a]	Proportion of Weight[b]	Discovery U4C Meta-analysis EAF[c]	Discovery U4C Meta-analysis OR (95% CI)	Discovery U4C Meta-analysis *p*-value	UK Biobank Replication EAF[c]	UK Biobank Replication OR (95% CI)	UK Biobank Replication *p*-value	Joint Meta-analysis OR (95% CI)	Joint Meta-analysis *p*-value
rs3826033	G / A	0.33	0.32	0.92 (0.88,0.98)	$4.1x10^{-03}$	0.13	0.86 (0.79,0.93)	$2.3x10^{-04}$	0.90 (0.86,0.94)	$9.5x10^{-06}$
rs2290202	G / T	0.29	0.3	0.93 (0.89,0.98)	$5.3x10^{-03}$	0.13	0.86 (0.79,0.93)	$1.9x10^{-04}$	0.91 (0.88,0.95)	$1.7x10^{-05}$
rs7180016	G / A	0.012	0.49	0.97 (0.93,1.01)	$1.3x10^{-01}$	0.16	0.90 (0.84,0.97)	$5.7x10^{-03}$	0.95 (0.92,0.99)	$7.3x10^{-03}$
rs11073961	A / G	0.049	0.35	0.97 (0.93,1.01)	$2.1x10^{-01}$	0.27	0.92 (0.87,0.98)	$7.5x10^{-03}$	0.95 (0.93,0.99)	$9.9x10^{-03}$
rs11207	C / T	0.0092	0.35	0.97 (0.93,1.02)	$2.1x10^{-01}$	0.24	0.93 (0.87,0.98)	$1.5x10^{-02}$	0.96 (0.93,0.99)	$1.6x10^{-02}$
rs2285937	A / G	0.0064	0.46	0.98 (0.94,1.02)	$3.1x10^{-01}$	0.16	0.90 (0.84,0.97)	$4.9x10^{-03}$	0.96 (0.93,0.99)	$2.3x10^{-02}$
rs3809583	A / G	0.0035	0.36	0.97 (0.93,1.01)	$1.2x10^{-01}$	0.32	0.96 (0.91,1.01)	$1.5x10^{-01}$	0.96 (0.93,1.00)	$3.5x10^{-02}$
rs1052532	T / C	0.0056	0.35	0.99 (0.94,1.03)	$6.3x10^{-01}$	0.27	0.92 (0.87,0.98)	$8.6x10^{-03}$	0.97 (0.93,1.00)	$5.4x10^{-02}$
rs7182610	C / T	<0.001	0.85	0.95 (0.90,1.01)	$8.6x10^{-02}$	0.91	1.01 (0.92,1.10)	$8.1x10^{-01}$	0.97 (0.92,1.02)	$1.9x10^{-01}$
rs11073986	C / T	0.032	0.65	1.01 (0.96,1.07)	$6.6x10^{-01}$	0.86	1.04 (0.96,1.12)	$3.6x10^{-01}$	1.02 (0.98,1.07)	$3.9x10^{-01}$
rs11638027	T / G	0.016	0.89	0.95 (0.89,1.02)	$2.0x10^{-01}$	0.86	1.01 (0.94,1.09)	$7.4x10^{-01}$	0.98 (0.93,1.03)	$4.6x10^{-01}$
rs4273013	G / A	0.011	0.96	1.01 (0.88,1.15)	$9.2x10^{-01}$	0.94	0.96 (0.86,1.07)	$4.3x10^{-01}$	0.98 (0.90,1.06)	$5.9x10^{-01}$
rs12911192	C / T	0.16	0.94	0.98 (0.87,1.11)	$7.6x10^{-01}$	0.93	0.98 (0.88,1.08)	$6.8x10^{-01}$	0.98 (0.91,1.06)	$6.1x10^{-01}$
rs1266479	T / C	0.062	0.73	0.99 (0.93,1.05)	$7.4x10^{-01}$	0.92	0.98 (0.89,1.08)	$6.6x10^{-01}$	0.99 (0.94,1.04)	$6.1x10^{-01}$
rs13380179	G / A	0.0026	0.28	0.99 (0.93,1.04)	$5.8x10^{-01}$	0.15	1.05 (0.97,1.13)	$2.1x10^{-01}$	1.01 (0.96,1.05)	$7.7x10^{-01}$
rs7177165	T / C	0.0022	0.64	1.02 (0.97,1.06)	$4.1x10^{-01}$	0.71	0.96 (0.91,1.02)	$2.0x10^{-01}$	1.00 (0.96,1.03)	$9.2x10^{-01}$
rs4932304	C / T	0.0026	0.28	0.97 (0.92,1.03)	$3.5x10^{-01}$	0.14	1.04 (0.97,1.12)	$2.6x10^{-01}$	1.00 (0.96,1.04)	$9.4x10^{-01}$
rs4932305	G / A	<0.001	0.28	0.98 (0.92,1.03)	$3.8x10^{-01}$	0.14	1.04 (0.97,1.12)	$2.6x10^{-01}$	1.00 (0.96,1.04)	$9.8x10^{-01}$
rs3751658	C / A	0.0034	0.28	0.98 (0.92,1.03)	$3.9x10^{-01}$	0.14	1.04 (0.97,1.12)	$2.6x10^{-01}$	1.00 (0.96,1.04)	$9.9x10^{-01}$
rs12441424	A / G	0.0031	0.56	0.99 (0.95,1.03)	$6.2x10^{-01}$	0.74	1.02 (0.96,1.08)	$4.7x10^{-01}$	1.00 (0.97,1.03)	$9.9x10^{-01}$

(E) *ACAP1* at 17p13.1 (Whole Blood)

SNP	Alleles[a]	Proportion of Weight[b]	Discovery U4C Meta-analysis EAF[c]	OR (95% CI)	*p*-value	UK Biobank Replication EAF[c]	OR (95% CI)	*p*-value	Joint Meta-analysis OR (95% CI)	*p*-value
rs35776863	A / G	0.49	0.85	1.08 (1.00,1.16)	$4.5x10^{-02}$	0.77	1.11 (1.04,1.18)	$1.5x10^{-01}$	1.10 (1.04,1.15)	$1.4x10^{-04}$
rs9892383	C / T	0.030	0.76	1.04 (0.98,1.09)	$1.7x10^{-01}$	0.73	1.10 (1.03,1.18)	$7.6x10^{-01}$	1.06 (1.02,1.11)	$3.6x10^{-03}$
rs5412	G / A	0.060	0.12	1.04 (0.97,1.12)	$2.6x10^{-01}$	0.17	1.09 (1.02,1.17)	$1.2x10^{-01}$	1.07 (1.02,1.12)	$8.0x10^{-03}$
rs4791423	A / C	0.0068	0.45	1.04 (1.00,1.09)	$3.3x10^{-02}$	0.34	1.03 (0.98,1.09)	$5.5x10^{-01}$	1.04 (1.01,1.08)	$1.8x10^{-02}$
rs35721044	T / C	0.031	0.84	1.11 (1.02,1.22)	$1.2x10^{-02}$	0.76	1.03 (0.97,1.10)	$1.6x10^{-01}$	1.06 (1.01,1.12)	$2.0x10^{-02}$
rs4796302	C / T	<0.001	0.30	0.98 (0.94,1.02)	$3.8x10^{-01}$	0.39	1.11 (1.06,1.17)	$3.0x10^{-01}$	1.03 (1.00,1.07)	$5.4x10^{-02}$
rs8081902	A / G	0.012	0.31	0.98 (0.93,1.02)	$3.1x10^{-01}$	0.39	1.11 (1.06,1.17)	$6.9x10^{-01}$	1.03 (1.00,1.06)	$7.2x10^{-02}$
rs8610	G / A	0.060	0.38	1.00 (0.96,1.04)	$1.0x10^{+00}$	0.41	1.07 (1.01,1.13)	$3.8x10^{-01}$	1.03 (0.99,1.06)	$1.1x10^{-01}$
rs3809827	A / G	0.18	0.44	1.04 (1.00,1.10)	$7.2x10^{-02}$	0.25	1.00 (0.94,1.07)	$5.6x10^{-01}$	1.03 (0.99,1.06)	$1.5x10^{-01}$
rs2135845	A / G	0.024	0.42	1.03 (0.99,1.08)	$1.1x10^{-01}$	0.50	1.01 (0.95,1.06)	$5.8x10^{-01}$	1.02 (0.99,1.05)	$1.7x10^{-01}$
rs3744251	G / A	0.012	0.25	1.04 (0.97,1.11)	$2.6x10^{-01}$	0.08	1.03 (0.93,1.13)	$2.5x10^{-01}$	1.03 (0.98,1.09)	$2.3x10^{-01}$
rs3027232	G / A	0.027	0.36	1.01 (0.97,1.05)	$5.6x10^{-01}$	0.22	1.04 (0.97,1.10)	$3.6x10^{-01}$	1.02 (0.98,1.05)	$2.8x10^{-01}$
rs4796492	A / C	0.0023	0.63	0.99 (0.94,1.03)	$5.0x10^{-01}$	0.70	0.99 (0.94,1.05)	$3.7x10^{-01}$	0.99 (0.95,1.02)	$4.6x10^{-01}$
rs8076718	G / A	0.0097	0.09	1.06 (0.96,1.18)	$2.3x10^{-01}$	0.11	0.99 (0.91,1.08)	$8.9x10^{-02}$	1.02 (0.96,1.09)	$5.5x10^{-01}$
rs11658922	A / C	0.0096	0.82	0.96 (0.91,1.02)	$1.8x10^{-01}$	0.89	1.06 (0.97,1.15)	$1.8x10^{-01}$	0.99 (0.94,1.04)	$6.6x10^{-01}$
rs8073152	A / G	0.011	0.23	1.01 (0.96,1.06)	$6.0x10^{-01}$	0.09	0.98 (0.90,1.08)	$7.7x10^{-01}$	1.01 (0.96,1.05)	$7.8x10^{-01}$
rs16956936	T / C	0.0025	0.89	1.03 (0.96,1.11)	$3.9x10^{-01}$	0.87	0.98 (0.91,1.06)	$1.1x10^{-01}$	1.01 (0.95,1.06)	$7.8x10^{-01}$
rs415704	C / T	0.010	0.50	0.99 (0.94,1.03)	$5.7x10^{-01}$	0.34	1.03 (0.97,1.09)	$5.0x10^{-01}$	1.00 (0.97,1.04)	$9.1x10^{-01}$
rs1567845	G / A	0.019	0.65	1.01 (0.97,1.05)	$6.6x10^{-01}$	0.61	0.99 (0.94,1.04)	$6.5x10^{-01}$	1.00 (0.97,1.03)	$9.4x10^{-01}$

(F) *LRRC25* at 19p13.11 (Whole Blood)

		Proportion	Discovery U4C Meta-analysis			UK Biobank Replication			Joint Meta-analysis	
**SNP**	**Alleles**[a]	**of Weight**[b]	**EAF**[c]	**OR (95% CI)**	***p*-value**	**EAF**[c]	**OR (95% CI)**	***p*-value**	**OR (95% CI)**	***p*-value**
rs11668719	C / T	0.25	0.5	1.06 (1.01,1.11)	1.1×10^{-02}	0.54	1.10 (1.05,1.16)	1.87×10^{-04}	1.08 (1.04,1.12)	1.2×10^{-05}
rs7257932	A / G	0.091	0.55	1.05 (1.01,1.10)	1.1×10^{-02}	0.67	1.08 (1.02,1.14)	7.01×10^{-03}	1.06 (1.03,1.10)	2.5×10^{-04}
rs13344313	A / G	0.16	0.68	1.06 (1.02,1.11)	6.6×10^{-03}	0.71	1.04 (0.98,1.10)	1.95×10^{-01}	1.05 (1.02,1.09)	3.2×10^{-03}
rs3795026	C / T	<0.001	0.54	1.04 (1.00,1.08)	5.1×10^{-02}	0.68	1.05 (0.99,1.11)	1.24×10^{-01}	1.04 (1.01,1.08)	1.3×10^{-02}
rs7251067	A / G	0.031	0.85	1.00 (0.95,1.06)	9.4×10^{-01}	0.86	1.14 (1.06,1.23)	6.70×10^{-04}	1.05 (1.00,1.10)	4.1×10^{-02}
rs2303693	T / C	0.13	0.86	1.00 (0.94,1.05)	8.9×10^{-01}	0.86	1.14 (1.05,1.23)	1.03×10^{-03}	1.04 (1.00,1.10)	6.5×10^{-02}
rs731945	C / T	0.011	0.39	1.04 (0.99,1.09)	1.2×10^{-01}	0.30	1.03 (0.97,1.09)	3.24×10^{-01}	1.03 (1.00,1.07)	6.9×10^{-02}
rs2277922	T / G	0.0028	0.58	0.97 (0.93,1.01)	1.2×10^{-01}	0.44	0.99 (0.94,1.04)	7.49×10^{-01}	0.98 (0.94,1.01)	1.6×10^{-01}
rs7254789	T / C	0.012	0.39	1.03 (0.98,1.09)	2.5×10^{-01}	0.18	1.03 (0.96,1.10)	4.42×10^{-01}	1.03 (0.99,1.08)	1.7×10^{-01}
rs17211813	C / T	0.0051	0.10	1.06 (0.96,1.17)	2.7×10^{-01}	0.13	1.03 (0.95,1.11)	4.43×10^{-01}	1.04 (0.98,1.11)	2.0×10^{-01}
rs8103177	G / A	<0.001	0.34	0.98 (0.94,1.02)	3.9×10^{-01}	0.16	0.98 (0.91,1.05)	5.99×10^{-01}	0.98 (0.95,1.02)	3.1×10^{-01}
rs2277921	G / A	<0.001	0.34	0.98 (0.94,1.02)	3.9×10^{-01}	0.16	0.98 (0.91,1.05)	6.10×10^{-01}	0.98 (0.95,1.02)	3.2×10^{-01}
rs2544725	C / A	0.0018	0.40	0.97 (0.94,1.01)	1.7×10^{-01}	0.40	1.01 (0.95,1.06)	7.99×10^{-01}	0.98 (0.95,1.02)	3.4×10^{-01}
rs374326	T / C	0.0062	0.21	0.97 (0.93,1.02)	2.5×10^{-01}	0.26	1.00 (0.94,1.06)	9.48×10^{-01}	0.98 (0.94,1.02)	3.5×10^{-01}
rs17214879	C / T	0.014	0.86	0.98 (0.91,1.04)	4.4×10^{-01}	0.81	0.98 (0.92,1.05)	6.17×10^{-01}	0.98 (0.94,1.03)	3.7×10^{-01}
rs8108878	T / C	<0.001	0.82	1.01 (0.95,1.08)	7.2×10^{-01}	0.84	0.94 (0.88,1.01)	8.88×10^{-02}	0.98 (0.94,1.03)	4.2×10^{-01}
rs169237	T / C	<0.001	0.40	0.98 (0.93,1.01)	2.3×10^{-01}	0.40	1.01 (0.96,1.06)	7.68×10^{-01}	0.99 (0.95,1.02)	4.3×10^{-01}
rs8108865	T / C	<0.001	0.82	1.01 (0.95,1.08)	7.1×10^{-01}	0.84	0.94 (0.88,1.01)	9.00×10^{-02}	0.98 (0.94,1.03)	4.3×10^{-01}
rs3212760	A / G	0.0027	0.38	0.98 (0.94,1.03)	4.7×10^{-01}	0.39	0.99 (0.94,1.04)	7.38×10^{-01}	0.99 (0.95,1.02)	4.4×10^{-01}
rs265558	C / T	0.0039	0.27	0.99 (0.94,1.04)	6.5×10^{-01}	0.39	0.98 (0.93,1.04)	5.51×10^{-01}	0.99 (0.95,1.02)	4.6×10^{-01}
rs3220	T / C	0.023	0.93	1.04 (0.93,1.15)	5.0×10^{-01}	0.90	1.01 (0.93,1.11)	7.52×10^{-01}	1.02 (0.95,1.10)	5.0×10^{-01}
rs17710624	G / A	0.017	0.65	0.99 (0.94,1.03)	5.7×10^{-01}	0.64	1.04 (0.99,1.10)	1.52×10^{-01}	1.01 (0.97,1.04)	6.4×10^{-01}
rs4808795	G / A	0.0099	0.25	1.02 (0.97,1.07)	4.9×10^{-01}	0.23	0.99 (0.93,1.06)	8.69×10^{-01}	1.01 (0.97,1.05)	6.6×10^{-01}
rs8103912	C / T	0.021	0.52	1.02 (0.97,1.06)	4.7×10^{-01}	0.37	0.96 (0.91,1.01)	1.50×10^{-01}	0.99 (0.96,1.03)	7.4×10^{-01}
rs1804826	G / T	0.0092	0.26	1.01 (0.97,1.06)	6.0×10^{-01}	0.23	0.99 (0.93,1.05)	7.27×10^{-01}	1.00 (0.97,1.04)	8.3×10^{-01}
rs12459566	C / T	0.072	0.69	1.04 (1.00,1.08)	7.8×10^{-02}	0.67	0.95 (0.90,1.00)	4.81×10^{-02}	1.00 (0.97,1.04)	8.5×10^{-01}
rs3746183	A / C	0.0076	0.74	1.00 (0.95,1.05)	1.0e+00	0.85	1.01 (0.94,1.09)	7.31×10^{-01}	1.00 (0.96,1.04)	8.5×10^{-01}
rs6512265	G / A	0.0044	0.72	1.03 (0.98,1.07)	2.8×10^{-01}	0.66	0.96 (0.91,1.01)	1.19×10^{-01}	1.00 (0.96,1.03)	8.6×10^{-01}
rs11672054	A / G	0.0069	0.90	1.00 (0.91,1.09)	9.3×10^{-01}	0.87	1.01 (0.93,1.09)	8.08×10^{-01}	1.00 (0.94,1.06)	9.0×10^{-01}
rs8101804	T / C	0.050	0.70	1.03 (0.99,1.08)	1.7×10^{-01}	0.66	0.95 (0.90,1.00)	5.91×10^{-02}	1.00 (0.96,1.03)	9.5×10^{-01}
rs17724992	A / G	0.0077	0.32	1.00 (0.95,1.04)	8.5×10^{-01}	0.27	1.01 (0.95,1.07)	7.36×10^{-01}	1.00 (0.96,1.04)	9.6×10^{-01}
rs4808652	G / A	<0.001	0.34	1.01 (0.96,1.06)	7.8×10^{-01}	0.07	0.97 (0.88,1.08)	6.14×10^{-01}	1.00 (0.96,1.04)	9.7×10^{-01}
rs12459782	T / C	0.050	0.70	1.03 (0.99,1.08)	1.6×10^{-01}	0.66	0.95 (0.90,1.00)	6.39×10^{-02}	1.00 (0.97,1.03)	9.8×10^{-01}

Abbreviations: CI: confidence interval; EAF: effect allele frequency; OR: odds ratio; SNP: single nucleotide polymorphism; U4C: Up for a Challenge

[a] Reference allele / effect allele

[b] Proportion of total weight attributed to SNP in gene prediction model

[c] Effect allele frequency in controls
