# Supplementary material for: Cis-eQTL-based trans-ethnic meta-analysis reveals novel genes associated with breast cancer risk
Source: PLoS Genet. 2017 Mar 31;13(3):e1006690. doi: 10.1371/journal.pgen.1006690 (PMC5391966; doi:10.1371/journal.pgen.1006690)
Supplement: S3 Table — Number of subjects removed from each cohort because of outlier principal components. (PDF) [file pgen.1006690.s003.pdf]

| Dataset (Source Dataset) | Race / Ethnicity | # PC Outliers Excluded |
|---|---|---|
| *Discovery* | | |
| AABC (AABC) | African | 0 |
| African (African Diaspora) | African | 23 |
| African American / Barbadian (African Diaspora) | African | 1 |
| CGEMS (CGEMS) | European | 6 |
| CPSII (BPC3) | European | 0 |
| EPIC (BPC3) | European | 7 |
| Latina Admixture (Latina Admixture) | Latina | 9 |
| MEC – European (BPC3) | European | 2 |
| MEC – Japanese (MEC) | East Asian | 51 |
| MEC – Latina (MEC) | Latina | 12 |
| NHS2 (BPC3) | European | 0 |
| PBCS (BPC3) | European | 8 |
| PLCO (BPC3) | European | 0 |
| Shanghai (Shanghai) | East Asian | 51 |
| *Replication* | | |
| UK Biobank | European | 335 |
