# Supplementary material for: Cis-eQTL-based trans-ethnic meta-analysis reveals novel genes associated with breast cancer risk
Source: PLoS Genet. 2017 Mar 31;13(3):e1006690. doi: 10.1371/journal.pgen.1006690 (PMC5391966; doi:10.1371/journal.pgen.1006690)
Supplement: S2 Fig — Forest plots of PrediXcan results for whole blood expression of (A) RCCD1, (B) ACAP1, and (C) LRRC25. (PDF) [file pgen.1006690.s005.pdf]

(A) ***RCCD1* at 15q26.1**

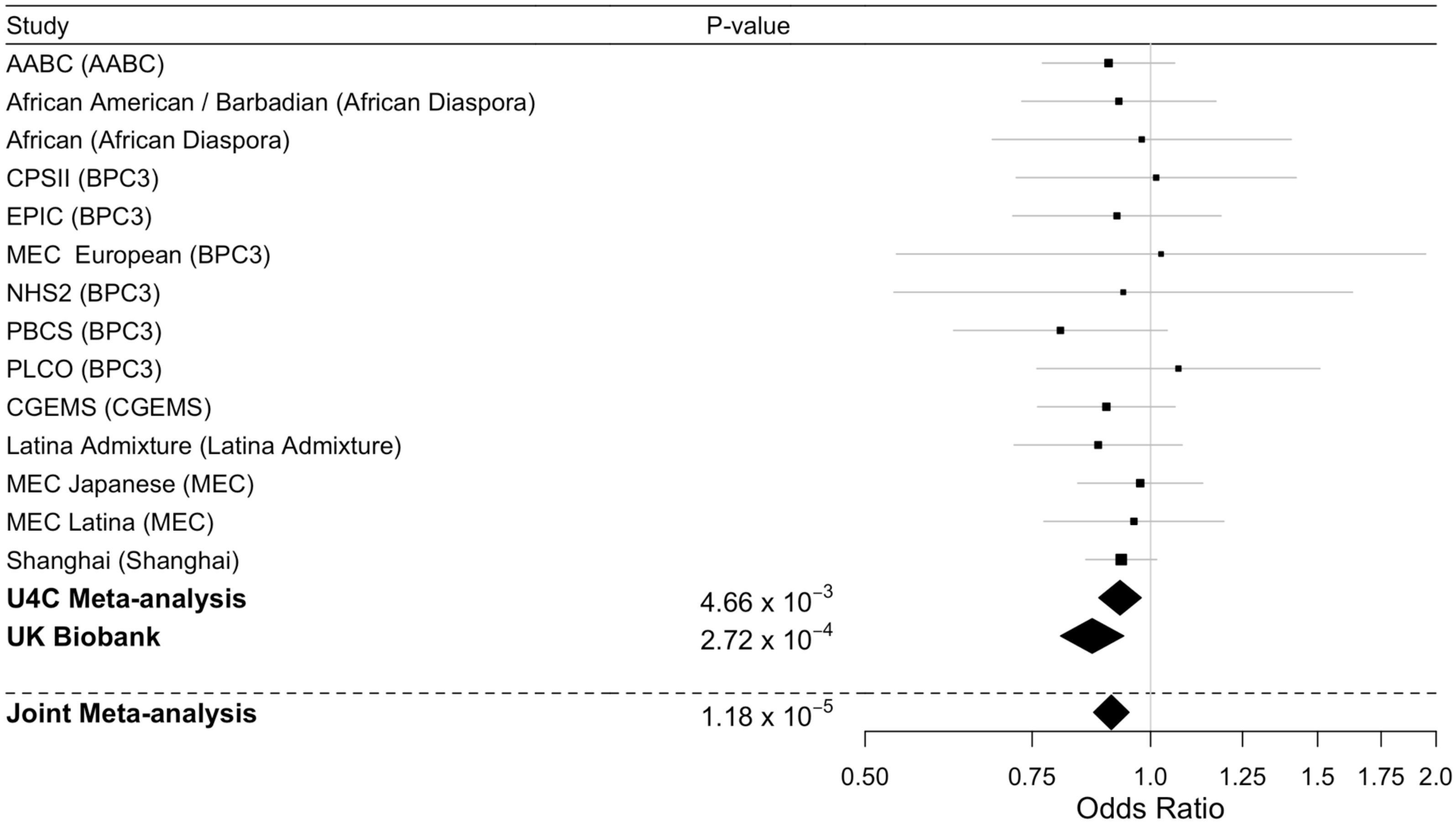

(B) ***ACAP1*** **at 17p13.1**

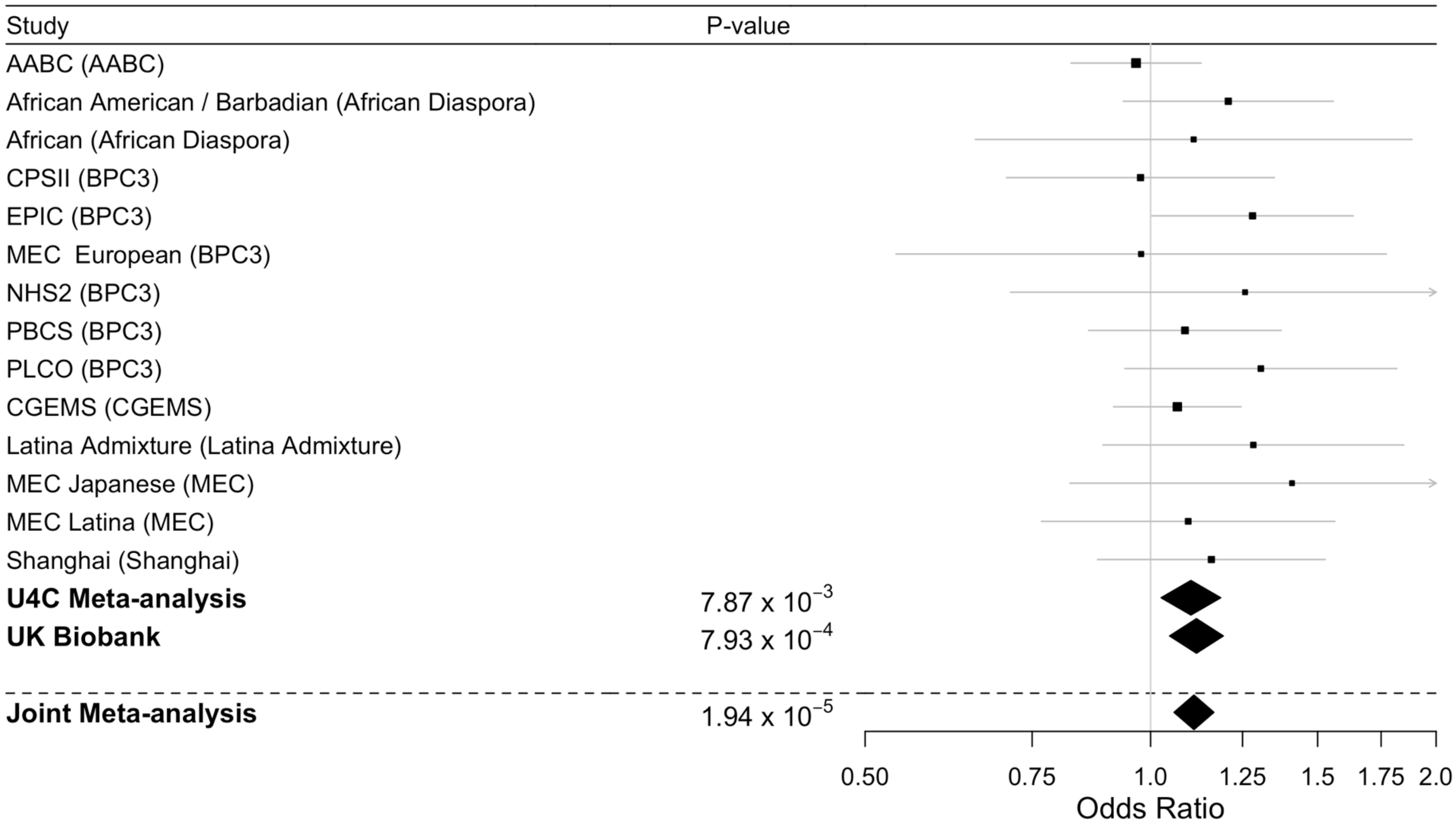

(C) *LRRC25* at 19p13.11

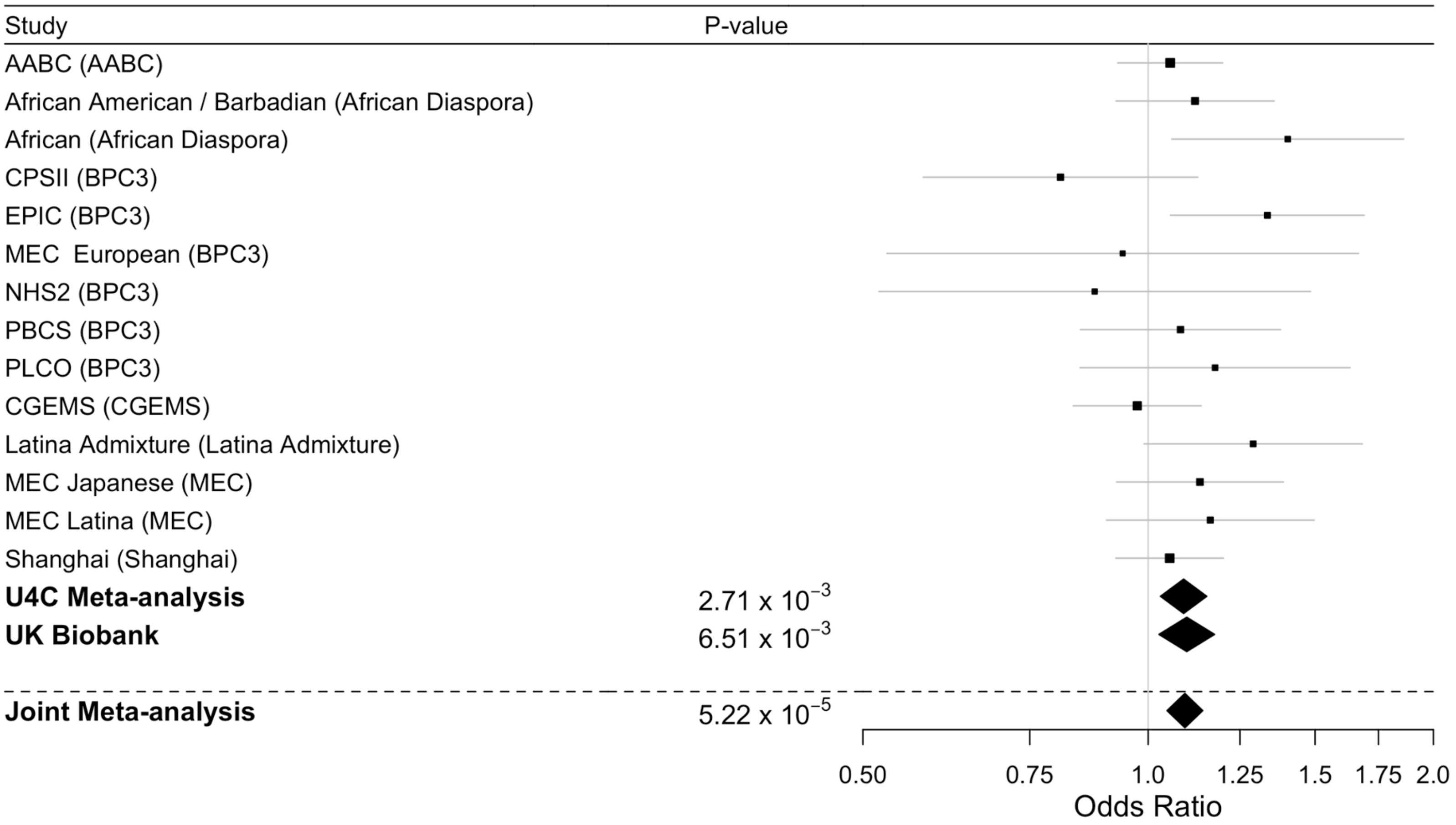

Note: the size of the boxes reflects the size of each study.
Abbreviations: AABC: African American Breast Cancer GWAS; African Diaspora: GWAS of Breast Cancer in the African Diaspora; BPC3: Breast and Prostate Cancer Cohort Consortium GWAS; CGEMS: Cancer Genetic Markers of Susceptibility Breast Cancer GWAS; CPSII: Cancer Prevention Study II; EPIC: European Prospective Investigation into Cancer and Nutrition; GWAS: genomx10-wide association study; Latina Admixture: Admixture Mapping for Breast Cancer in Latinas;

MEC: Multiethnic Cohort GWAS in African Americans, Latinos, and Japanese; NHS2: Nurses' Health Study 2; PBCS: Polish Breast Cancer Study; PLCO: Prostate, Lung, Colorectal, and Ovarian Cancer Screening Trial; Shanghai: Shanghai Breast Cancer Genetics Study; U4C: Up for a Challenge
