# Supplementary material for: Cis-eQTL-based trans-ethnic meta-analysis reveals novel genes associated with breast cancer risk
Source: PLoS Genet. 2017 Mar 31;13(3):e1006690. doi: 10.1371/journal.pgen.1006690 (PMC5391966; doi:10.1371/journal.pgen.1006690)
Supplement: S3 Fig — Volcano plots of PrediXcan results for associations between breast cancer risk and the imputed expression of (A) 4,469 genes based on breast tissue and (B) 9,768 genes based on whole blood (genes with beta estimates outside three standard deviations from the mean were removed from the plots– 4 for breast tissue and 23 for whole blood). (PDF) [file pgen.1006690.s006.pdf]

(A) Imputed expression based on breast tissue

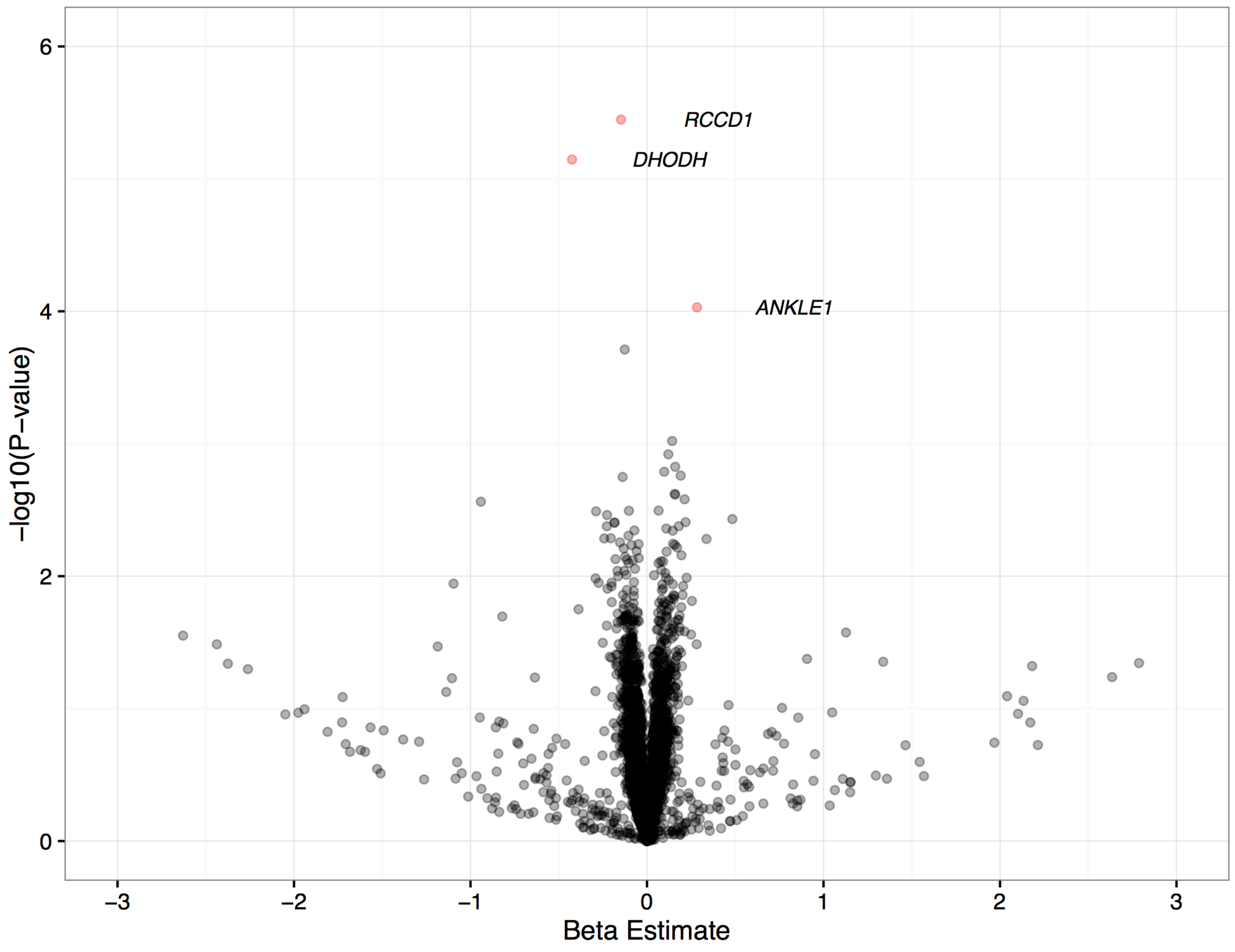

(B) Imputed expression based on whole blood

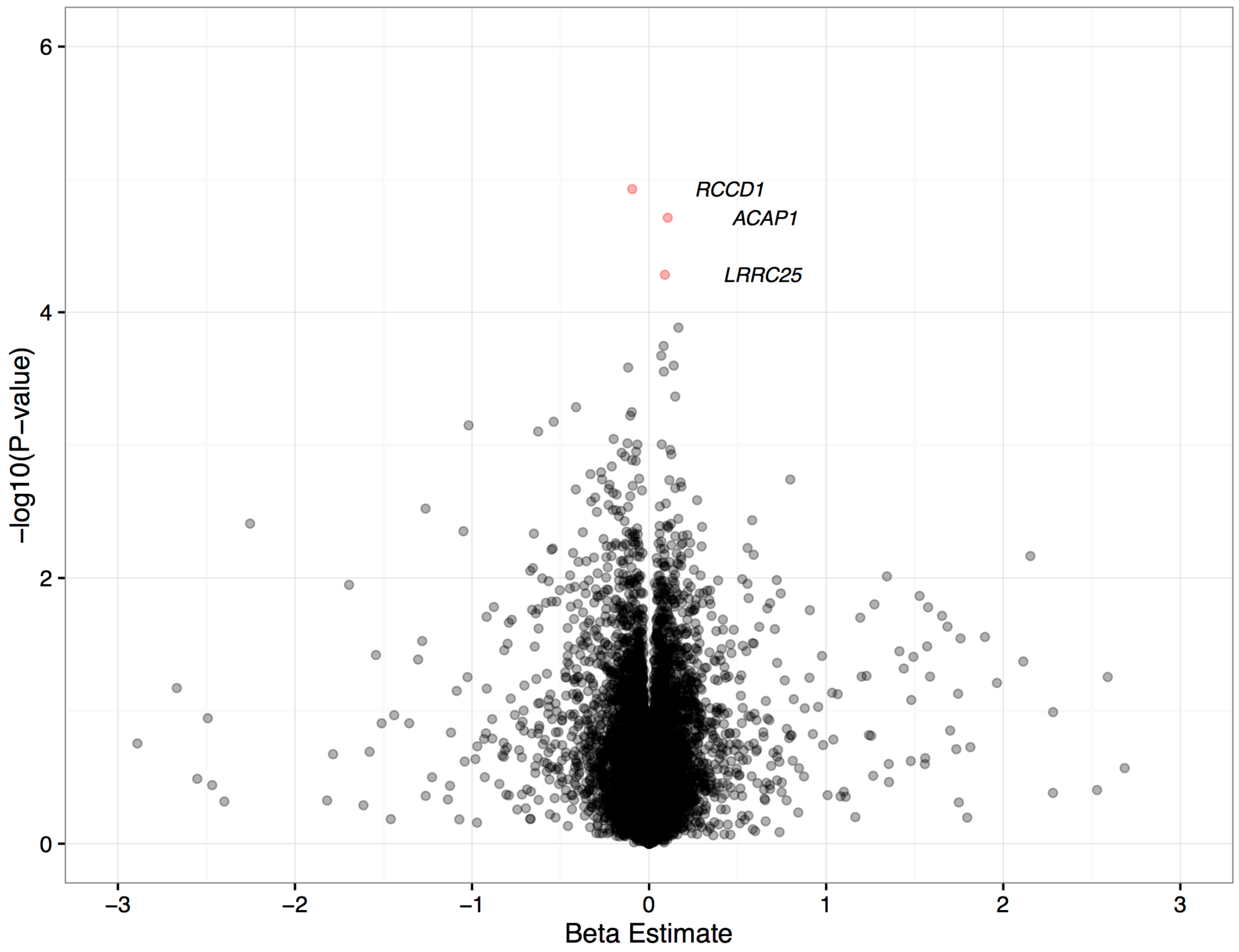

The x-axis displays the beta effect sizes for associations between gene expression and breast cancer risk. The y-axis displays -$\log_{10}$(*p*-values) as a measure of statistical significance.
